# Conformational Re‐engineering of Porphyrins as Receptors with Switchable N−H⋅⋅⋅X‐Type Binding Modes

**DOI:** 10.1002/anie.201907929

**Published:** 2019-09-24

**Authors:** Karolis Norvaiša, Keith J. Flanagan, Dáire Gibbons, Mathias O. Senge

**Affiliations:** ^1^ School of Chemistry SFI Tetrapyrrole Laboratory Trinity Biomedical Sciences Institute Trinity College Dublin The University of Dublin 152–160 Pearse Street Dublin 2 Ireland; ^2^ Institute for Advanced Study (TUM-IAS) Technische Universität München Lichtenberg-Str. 2a 85748 Garching Germany

**Keywords:** conformational design, nonplanar porphyrins, porphyrinoids, pyrophosphate, sensors

## Abstract

The selectivity and functional variability of porphyrin cofactors are typically based on substrate binding of metalloporphyrins wherein the pyrrole nitrogen units only serve to chelate the metal ions. Yet, using the porphyrin inner core system for other functions is possible through conformational engineering. As a first step towards porphyrin “enzyme‐like” active centers, a structural and spectroscopic study of substrate binding to the inner core porphyrin system shows that a highly saddle‐distorted porphyrin with peripheral amino receptor groups (**1**, 2,3,7,8,12,13,17,18‐octaethyl‐5,10,15,20‐tetrakis(2‐aminophenyl)porphyrin) coordinates analytes in a switchable manner dependent on the acidity of the solution. The supramolecular ensemble exhibits exceptionally high affinity to and selectivity for the pyrophosphate anion (2.26±0.021)×10^9^ m ^−1^. ^1^H NMR spectroscopic studies provided insight into the likely mode of binding and the characterization of atropisomers, all four of which were also studied by X‐ray crystallography.

## Graphical abstract

**An acid‐activated** porphyrin probe for small‐molecule detection is demonstrated, highlighting the colorimetric change of the binding event. The key feature is substrate coordination to the inner core system assisted via peripheral interactions.

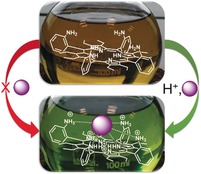

For the past few decades, the inner core system of conformationally designed nonplanar porphyrins has attracted scientists as an excellent host for various metals^1^ with highly tunable basicity.^2^ Such porphyrins recently found their first application as organocatalysts2a, 2b and promoters for dioxygen/hydrogen peroxide interconversion.^3^ This indicates that specific core N−H⋅⋅⋅substrate interactions can be achieved via macrocycle engineering.^4^ Since the beginning of porphyrin structural chemistry,5a weak interactions have been observed in crystals.^5^ Densely packed free base porphyrin systems that encapsulate substrates in their lattices are well known.^4^, 5b, 5c, 6 However, a limitation of X‐ray crystallography is the structural determination of monocrystalline solids, achieved by recrystallization from saturated solutions.^7^ Alternative spectroscopic detection of N−H⋅⋅⋅X‐type binding in porphyrin solutions is almost impossible without specially designing the binding pocket, as solvation and dilution drastically affect weak interactions by dispersing the binding agent to its surroundings. Planar porphyrins are more difficult to protonate than their nonplanar counterparts due to the penalty paid for extra distortion required upon protonation.^8^ Therefore, the respective symmetric porphyrin dications have yet to find use as either functional or selective anion receptors under normal laboratory conditions.^9^ Moreover, the porphyrin inner core imine and amine units of planar analogues are usually not involved in intermolecular interactions due to the “shielding” properties of the macrocycle system.^10^ Distortion can cause an increase in the degree of outwards orientation of the inner pyrrolic entities, making these positions more basic and accessible to substrates, thereby creating an “active center”.2a, 4

To activate the imine and amine moieties, we chose ring puckering by steric strain. Since sterically overcrowded porphyrins can have significantly saddle‐distorted 3D conformations,^1^, ^3^, 5c, 7, 11 a combination with additional coordinating sites on the periphery of porphyrins can yield porphyrinoid receptors with multiple binding sites to produce a situation comparable to the enzyme lock‐and‐key model^4^ at a molecular level. Therein, the interplay between all peripheral substituents (i.e., *peri* interactions and peripheral control via functional groups) would result in a well‐defined active site directing to the inner core system for substrate/analyte binding and recognition (Scheme 1).

**Scheme 1:**
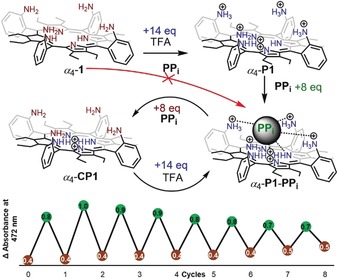
Graphical representation of the proposed switchable *α_4_*‐**P1**‐**PP_i_** complex system and repeated cycles (detected using absorbance intensity at 472 nm) obtained from a 5.56×10^−6^ m *α_4_*‐**1** solution and 8:14 equiv. (**PP_i_**:TFA) ratio in CHCl_3_.

A modified Lindsey condensation reaction of 3,4‐diethylpyrrole^12^ and 2‐nitrobenzaldehyde, using BF_3_⋅Et_2_O as a catalyst and the oxidant DDQ, yielded the intermediate product 2,3,7,8,12,13,17,18‐octaethyl‐5,10,15,20‐tetrakis(2‐nitrophenyl)porphyrin (H_2_OET_NO2_PP) in 29 % yield. This was subsequently reduced to the tetraaminoporphyrin **1** in 79 % yield using SnCl_2_ under acidic conditions (HCl) (Scheme S1). Further purification (column chromatography and recrystallization using liquid–liquid diffusion in CHCl_3_ and MeOH) yielded crystals of *α_4_*‐**1** confirming the (↑↑↑↑) atropisomer conformation of the most polar fraction isolated from column chromatography (Figures S42 and S44). The other atropisomeric entities could not yet be separated due to their similar polarities. Additionally, porphyrin [*α_4_*‐**P1** ^6+^][SO_4_^2−^][HSO_4_^−^]_4_ was crystallized from the acidic MeOH solution (in the presence of sulfuric acid) and the crystalline compound was analyzed by X‐ray crystallography, showing multiple hydrogen‐bonding events.

Many cofactors, enzyme substrates, and DNA are anionic in nature.^13^ Among anions of current interest,^14^ the pyrophosphate anion has attracted particular attention due to its biological relevance,^15^ and significant efforts have been made to develop more potent pyrophosphate sensors.^16^ To study the binding capabilities of the isolated tetraaminoporphyrin *α_4_*‐**1**, UV/Vis titrations were carried out with tris(tetrabutylammonium) hydrogen pyrophosphate (**PP_i_**) in the presence of TFA. The Soret band of the hexaprotonated porphyrin *α_4_*‐**P1** (488 nm) was found to be redshifted by 27 nm compared to the neutral porphyrin *α_4_*‐**1** (461 nm), while the new Soret band of *α_4_*‐**P1**‐**PP_i_** complex exhibited a bathochromic shift of 10 nm (472 nm) compared to *α_4_*‐**1** (Figure 1). Complex formation was accompanied by a strong reduction of the main Q‐band of the protonated porphyrin *α_4_*‐**P1** (at 696 nm) while two Q‐bands arose at new positions (616 and 673 nm), similar to what is observed for the metalloporphyrin complexes^17^ (higher symmetry in comparison to the porphyrins). Two isosbestic points were identified at 483 and 684 nm. This was followed by a rapid color change from yellow/brown to green. Most often, the variation of the porphyrin color indicates electronic or geometrical changes to the macrocyclic system9b, 18 which, in this instance, refers to the substrate–core interaction.

**Figure 1:**
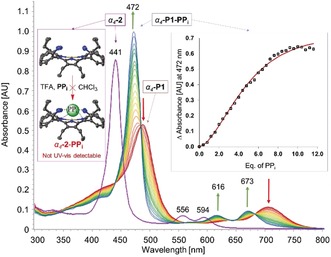
Comparison of the UV/Vis titration of *α_4_*‐**1** (5.56×10^−6^ m) and nickel(II) porphyrin *α_4_*‐**2** (5.56×10^−6^ m) performed in CHCl_3_ with pyrophosphate (0–12 equiv.) and TFA (100 equiv.).

To confirm and further investigate N−H⋅⋅⋅X‐type complexation, the inner core system was blocked from any potential pyrrole–substrate interactions by Ni^II^ insertion. The isolated *α_4_*‐Ni^II^OET_am_PP (*α_4_*‐**2**) (metalated analogue of *α_4_*‐**1**) showed no observable spectroscopic changes in UV/Vis titration studies (Figure 1). Furthermore, titration of *α_4_*‐**1** with TFA in the presence of **PP_i_** revealed the formation of core diprotonated porphyrin *α_4_*‐**CP1** before transitioning to the *α_4_*‐**P1**‐**PP_i_** system (Figure S46). This, therefore, indicates that: 1) the inner core system plays an essential role in complex formation; 2) protonation of the core imine nitrogens takes place first, followed by the peripheral amines; 3) the charge‐carrying peripheral ammonium groups are necessary for the complex formation in order to stabilize the corresponding substrate via the combination of electrostatic and hydrogen‐bonding interactions.^19^ It should be noted that the nonplanar analogue without any peripheral coordinating groups (H_4_OETPP^2+^) was incapable of forming any subsequent adducts (Figure S47).

Given that the protonation of the peripheral amines forms an active probe for **PP_i_** detection, a molecular switch between *α_4_*‐**P1**‐**PP_i_** and the substrate‐free *α_4_*‐**CP1** form was developed. Following the use of 14 equiv. of TFA, the formation of the hexaprotonated porphyrin *α_4_*‐**P1** was observed. Addition of 8 equiv. of **PP_i_** promptly formed *α_4_*‐**P1**‐**PP_i_**, while another 8 equiv. immediately regenerated the substrate‐free form (*α_4_*‐**CP1**), a result of the basicity introduced with the **PP_i_** salt. Therefore, a lack of peripheral charge via deprotonation with excess **PP_i_** leads to a destabilized complex. In order to reform the *α_4_*‐**P1**‐**PP_i_**, acidity must be restored with an additional 14 equiv. of TFA. These cycles could be repeated at least eight times (in an 8:14 equiv. (**PP_i_**:TFA) ratio) (Scheme 1).^20^ The molecular switch between active and inactive forms highlights the reversibility and reusability of the current system.

The anion‐recognition properties of *α_4_*‐**P1** were studied in CHCl_3_ with various anions in the form of TBA salts (Figures S48 and S49) and different acids (Figure S50), using UV/Vis spectroscopy. In addition to **PP_i_**, four substrates—tetrabutylammonium bisulfate (**BS**), tetrabutylammonium phosphate monobasic (**MP**), methanesulfonic acid (**MSA**), and benzenesulfonic acid (**BSA**)—were pinpointed as complexing substrates. Similar UV/Vis spectral profiles were observed for all complexes; however, the main Soret bands (depending on its position) can be assigned to the anionic moiety present within the analyte (sulfonic at ≈464 nm and phosphonic at ≈471 nm)(Figure 2). This finding—while preliminary—suggests that the geometrical and electronic properties of phosphoric and sulfonic moieties influence the porphyrin–analyte complex formation, similar to the proposed “lock‐and‐key” concept.^4^

**Figure 2:**
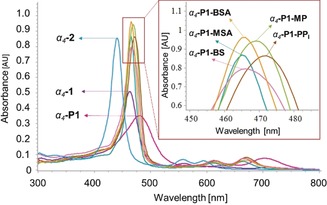
Overlay of the UV/Vis spectra (5.56×10^−6^ m) of *α_4_*‐**1**, *α_4_*‐**P1**, *α_4_*‐**2**, and complex systems (*α_4_*‐**P1**‐**MSA**, *α_4_*‐**P1**‐**BSA**, *α_4_*‐**P1**‐**BS**, *α_4_*‐**P1**‐**PP_i_**, *α_4_*‐**P1**‐**MP**) in CHCl_3_.

In operational terms, the affinities for anions were found to decrease according to the following sequence: **PP_i_** > **BS** > **MP** as observed from UV/Vis displacement studies (Figures S51 and S54). The affinities of **MSA** and **BSA** were not investigated due to their poor solubility in CHCl_3_. Stoichiometry for *α_4_*‐**P1** was determined using Job's plots (Figure S52). **BS** was found to interact with *α_4_*‐**P1** in a 1:2 (host:guest) ratio and **MP** and **PP_i_** in a 1:1 ratio (Figure S54). The binding constants were calculated using ReactLab software^21^ and the corresponding binding parameters are presented in Table 1. UV/Vis titration plots with **MP** show a well‐fitted pattern [sum of squares (ssq)=0.11 and virtual displacement (*δ*_r_)=2.54×10^−3^] correlating to the 1:1 binding mode. Presumably, *α_4_*‐**P1** cannot accommodate two **MP** units, thus leading to only one entity binding in the system. Complexation with **BS** displayed a best fitting pattern fixed into the 1:2 binding mode (ssq=0.11 and *δ*_r_=2.33×10^−3)^. Interestingly, **PP_i_** showed a relatively poor fit (ssq=3.05 and *δ*_r_=0.0132) when fixed into the 1:1 binding mode with *α_4_*‐**P1**. It can be postulated that **PP_i_** can act as a bidentate‐type substrate in complex formation with *α_4_*‐**P1**. Thereby, complexation with **PP_i_** had to be treated as a 1:2 binding mode during calculations despite the 1:1 ratiometric binding suggested by the Job's plot (Figure S54). After the new fitting (*K*_1_=0 and 1:2 binding mode), the *K*_2_ value was calculated with a sixfold lower ssq value (0.51) and improved *δ*_r_ value (5.4×10^−3^), leading to a much better fit (Table 1). In terms of association constants, the previously performed displacement studies *K*_**MP**_ < *K*_**BS**_ < *K*_**PPi**_ corresponded well with calculated values [(1.04±0.014)×10^5^ < (3.44±0.389)×10^6^ < (2.26±0.021)×10^9^, respectively]. Not only does this show a very high affinity towards pyrophosphate in contrast to other tetrapyrrole systems,16b, 16f, 16g, 16k retaining all the porphyrin functionality as a chromophore (aromaticity of the macrocycle), but also a remarkably high selectivity and tolerance in the presence of the most common interfering anions (Figure S55).

**Table 1:** Binding constants and binding data of *α_4_*‐**P1** porphyrin with different anionic analytes (**MP**, **BS**, **PP_i_**) determined in CHCl_3_.

Anion^[a]^	Binding mode^[b]^	*K*_1_^[c]^	*K*_2_^[c]^	*δ_r_* ^[d]^ (×10^−3^)	ssq^[e]^
**MP**	1:1	(1.04±0.014)×10^5^	–	2.54	0.11
**BS**	1:2	(3.44±0.389)×10^6^	(5.25±0.011)×10^5^	2.33	0.11
**PP_i_**	1:2	–	(2.26±0.021)×10^9^	5.40	0.51
**PP_i_**	1:1	(4.45±0.071)×10^4^	–	13.2	3.05

[a] Analyte. [b] Guest‐to‐host interaction. [c] Calculated association constants with estimated uncertainties. [d] Virtual displacement. [e] Sum of squares.

To further investigate the N−H⋅⋅⋅X‐type interactions in *α_4_*‐**P1** complex systems, we carried out ^1^H NMR studies in CD_3_CN. It should be noted that aggregation and precipitation in a concentrated solution of CHCl_3_ prevented any further ^1^H NMR analysis in CDCl_3_. Moreover, the low sensitivity of *α_4_*‐**P1** as a receptor for analytes in CH_3_CN was detected;^22^ thus, excess amounts of substrates were used in the following studies. ^1^H NMR spectra in CD_3_CN were recorded with isomerically pure *α_4_*‐**1** in the presence of TFA. In contrast to the *α_4_*‐**P1** spectra, the addition of **MSA**, **BSA**, and **BS** resulted in new resonances in the aromatic and aliphatic regions (Figure S56). Complex formation of *α_4_*‐**P1**‐**BS**, *α_4_*‐**P1**‐**MSA**, and *α_4_*‐**P1**‐**BSA** was suggested by the emergence of two sharp inner core proton signals. Presumably, substrate interactions with the inner core system restrict rapid dynamic exchange by blocking the macrocyclic cavity, resulting in the emergence of two differently shifted proton signals. The ability of the substrates to accept hydrogen bonds from the donor cavity determines the blocking properties by affecting the exchange rates (Figure 3).

**Figure 3:**
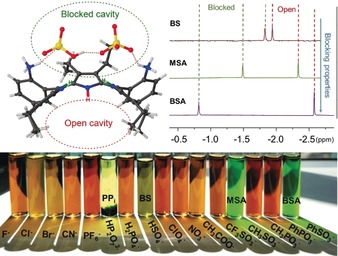
Top: Schematic representation of the “blocked” and “open” cavities observed in the crystal structure and ^1^H NMR spectra of *α_4_*‐**P1**‐**BS**, *α_4_*‐**P1**‐**MSA**, *α_4_*‐**P1**‐**BSA** inner core systems in CD_3_CN. Bottom: visual representation of *α_4_*‐**P1** interacting with different analytes in CH_3_CN.

As mentioned above, the isolation of all individual atropisomers was not yet accomplished for the free‐base porphyrin **1** form. However, the introduction of Ni^II^ to the macrocycle system eliminated any possible inner N−H tautomerism,^23^ leading to an increase in structural symmetry.^18^ This was achieved by stirring the atropisomeric mixture **1** in boiling toluene (120 °C) and in the presence of 5 equiv. of nickel(II) acetylacetonate for 4 hours, yielding the nickel(II) porphyrin **2** in 93 % yield as a mixture of atropisomers. Thin‐layer chromatography (SiO_2_) showed excellent separation in DCM and, subsequently, allowed isolation of the individual atropisomers. The relative amounts of individual components (*α*,*β*,*α*,*β*‐**2** 13 %, *α_2_*,*β_2_*‐**2** 23 %, *α_3_*,*β*‐**2** 49 %, and *α_4_*‐**2** 15 %) correlated well with previously reported planar free‐base analogues (four atropisomers obtained in 1:2:4:1 statistical abundance ratio) with great stability towards isomerization.^19^, ^24^ All of the isolated atropisomers were recrystallized using liquid–liquid diffusion (CHCl_3_/MeOH) and confirmed via X‐ray crystallography (Figures S42 and S44).^25^ To our knowledge, this is the first example of porphyrin atropisomerism where all four isomers have been structurally characterized.

To investigate N−H⋅⋅⋅X‐type interactions within the different atropisomers of **2**, ^1^H NMR studies were carried out in CD_3_CN with the addition of **MSA** (as a demetalating and complexing agent) (Figure S57). The distribution of the inner N−H signals of the individual atropisomers was very different (Figure 4). *α,β,α*,*β*‐**P1**‐**MSA** (Figure 4 a) showed only one N−H signal due to the symmetrical binding motif on both sides of the plane. In the case of the *α_2_*,*β_2_*‐**P1**‐**MSA** configuration (Figure 4 b), there are potentially three substrates which interact with the N−H groups: two identically, and one on the other side of the plane leaving one N−H proton “inactive”. In *α*,*β_3_*‐**P1**‐**MSA** (Figure 4 c), four different proton signals were observed due to the highly unsymmetrical system. As previously described, *α_4_*‐**P1**‐**MSA** (Figure 4 d) complexation is followed on one side of the plane making the “blocked” and “open” cavities, resulting in two differently shifted proton signals. Overall, ^1^H NMR spectroscopy was successfully exploited as an instrument for the detection of porphyrin–analyte complexes and could be employed as a tool for the determination of the corresponding conformations.

**Figure 4:**
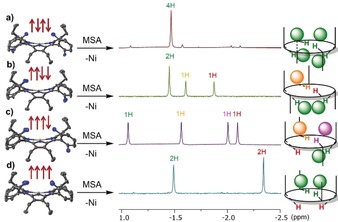
Crystal structures of atropisomers of **2** and ^1^H NMR spectra (inner core region) of *α*,*β*,*α*,*β*‐**P1**‐**MSA** (a), *α_2_*,*β_2_*‐**P1**‐**MSA** (b), *α_3_*,*β*‐**P1**‐**MSA** (c), and *α_4_*‐**P1**‐**MSA** (d) recorded in CD_3_CN along with illustrations of the proposed intramolecular interactions of MSA with different **P1** atropisomers.

In this study, we have detailed our insights into the acid‐activated nonplanar porphyrin *α_4_*‐**P1** N−H⋅⋅⋅X‐type binding motifs observed in solution. High selectivity towards substrates containing phosphonic or sulfonic moieties was spectrophotometrically detected. This was accompanied by a distinct color transition unlocking the potential of superstructured free‐base porphyrins for colorimetric anion recognition. The highest affinity identified for pyrophosphate is rationalized in terms of its ability to form a stronger supramolecular complex with *α_4_*‐**P1** compared to other anions tested. Presumably, this reflects the combined benefit of several favorable interactions, including electrostatic and hydrogen bonding. ^1^H NMR analyses of various complexes revealed highly different inner core proton signals, suggesting a combination of “blocked” and “open” cavities due to the binding event. The proper tuning of various weak interactions combined with a “turned‐on” approach, as delivered by the system's protonation, may provide a general strategy for the development of metal‐free porphyrin‐based probes for a wide range of analytes and hints at a potential path towards artificial porphyrin‐based enzyme‐like catalysts.

## Conflict of interest

The authors declare no conflict of interest.

## Supporting information

As a service to our authors and readers, this journal provides supporting information supplied by the authors. Such materials are peer reviewed and may be re‐organized for online delivery, but are not copy‐edited or typeset. Technical support issues arising from supporting information (other than missing files) should be addressed to the authors.

Supplementary
