# Supplementary material for: Conformational Re‐engineering of Porphyrins as Receptors with Switchable N−H⋅⋅⋅X‐Type Binding Modes
Source: Angew Chem Int Ed Engl. 2019 Sep 24;58(46):16553–7. doi: 10.1002/anie.201907929 (PMC6899560; doi:10.1002/anie.201907929)
Supplement: Supplementary file 1 — Supplementary [file ANIE-58-16553-s001.pdf]

Angewandte Chemie
Eine Zeitschrift der Gesellschaft Deutscher Chemiker

Supporting Information

# Conformational Re-engineering of Porphyrins as Receptors with Switchable N–H···X-Type Binding Modes

*Karolis Norvaiša, Keith J. Flanagan, Dáire Gibbons, and Mathias O. Senge**

anie_201907929_sm_miscellaneous_information.pdf

SUPPORTING INFORMATION

# Table of Contents

Experimental Procedures ........................................................................................2
General Materials and Methods ........................................................................................2
Synthesis and Characterization of Compounds ........................................................................................3
*Synthesis and Characterization of* ***$H_2OET_{NO_2}PP$*** ........................................................................................*3*
*Synthesis and Characterization of $\alpha_4$-**1*** ........................................................................................*7*
*Synthesis and Characterization of α,β,α,β-**2*** ........................................................................................*11*
*Synthesis and Characterization of $\alpha_2,\beta_2$-**2*** ........................................................................................*15*
*Synthesis and Characterization of $\alpha_3,\beta$-**2*** ........................................................................................*19*
*Synthesis and Characterization of $\alpha_4$-**2*** ........................................................................................*23*
Results and Discussion ........................................................................................27
Structural Determination of Receptor-Substrate Complexes and Atropisomers ........................................................................................27
UV-Vis Spectrophotometry Titration Studies ........................................................................................32
Binding and Competitive Studies ........................................................................................35
$^1$H NMR Studies of Porphyrin-Substrate Complexes ........................................................................................37
References ........................................................................................39

SUPPORTING INFORMATION

## Experimental Procedures

### General Materials and Methods

All chemicals were supplied by Sigma Aldrich, Acros Organics, Fluka, Frontier Scientific, and Fischer and handled without further purification unless otherwise stated. Anhydrous DCM used in large scale reactions, obtained *via* drying with phosphorus pentoxide followed by distillation, while smaller amounts of EtOH and toluene were used as commercially available HPLC grade solvents. Chloroform used in titration studies was neutralized with potassium carbonate and filtered over celite before carrying out any measurements.

**Analytical thin layer chromatography** was performed using silica gel 60 (fluorescence indicator F254, precoated sheets, 0.2 mm thick, 20 cm × 20 cm; Merck) or aluminum oxide 60 (neutral, F254; Merck) plates and visualized by UV irradiation ($\lambda$ = 254 nm).

**Column chromatography** was carried out using Fluka Silica Gel 60 (230–400 mesh; Merck) or aluminum oxide (neutral, activated with 6% $H_2O$, Brockman Grade III). Mobile phases are given as (*v/v*).

**Mass spectrometry** was performed with a Q-Tof Premier Waters MALDI quadrupole time-of-flight (Q-TOF) mass spectrometer equipped with Z-spray electrospray ionization (ESI) and matrix-assisted laser desorption ionization (MALDI) sources in positive mode with DCTB *trans*-2-[3-(4-*tert*-butylphenyl)-2-methyl-2-propenylidene]malononitrile (DCTB) as the matrix. ESI mass spectra were acquired in positive modes as required, using a Micromass time-of-flight mass spectrometer (TOF) interfaced to a Waters 2960 HPLC or a Bruker microTOF-Q III spectrometer interfaced to a Dionex UltiMate 3000 LC. Atmospheric pressure chemical ionization (APCI) experiments were performed on a Bruker microTOF-Q III spectrometer interfaced to a Dionex UltiMate 3000 LC.

**Melting points** are uncorrected and were measured with a Stuart SP-10 melting point apparatus.

**NMR spectra** were recorded on a Bruker Advance III 400 MHz, a Bruker DPX400 400 MHz and an Agilent 400 spectrometer for $^1H$ (400.13 MHz) and $^{13}C$ (100.61 MHz) NMR spectra. A Bruker Ultrashield 600 spectrometer was employed for $^1H$ (600.13 MHz) and $^{13}C$ (150.90 MHz) NMR spectra. All NMR experiments were performed at 25 ºC. Resonances $\delta$ are given in ppm units and referenced to the deuterium peak in the NMR solvents, $d_3$-acetonitrile ($\delta_H$ = 1.94 ppm, $\delta_C$ = 1.32, 118.26 ppm). $CDCl_3$ ($\delta_H$ = 7.26 ppm, $\delta_C$ = 77.2 ppm). Signal multiplicities are abbreviated as follows: singlet = s, doublet = d, triplet = t, multiplet = m.

**Single crystal X-ray crystallography:** Diffraction data for all compounds were collected on a Bruker APEX 2 DUO CCD diffractometer using graphite-monochromated Mo-$K_\alpha$ ($\lambda$ = 0.71073 Å) and Incoatec IµS Cu-$K_\alpha$ ($\lambda$ = 1.54178 Å) radiation. Crystals were mounted on a MiTeGen MicroMount and collected at 100(2) K using an Oxford Cryosystems Cobra low-temperature device. Data were collected using omega and phi scans and were corrected for Lorentz and polarization effects using the APEX software suite.[1] Data were corrected for absorption effects using the multi-scan method (SADABS).[2]

**UV-Vis absorption measurements** were performed using a Shimadzu MultiSpec-1501. The photophysical measurements were carried out in chloroform as a solvent. Cuvettes used in the corresponding studies was Quartz Glass 10mm 6030-UV.

**IR measurements** were done on a PerkinElmer Spectrum 100 FT-IR.

**Shelnutt's NSD** (normal structural decomposition) method was used to delineate, quantify and illustrate the various distortions modes present in the tetrapyrrole macrocycles.[3] NSD calculations were performed with the NSD GUI version of the program.[4]

## Synthesis and Characterization of Compounds

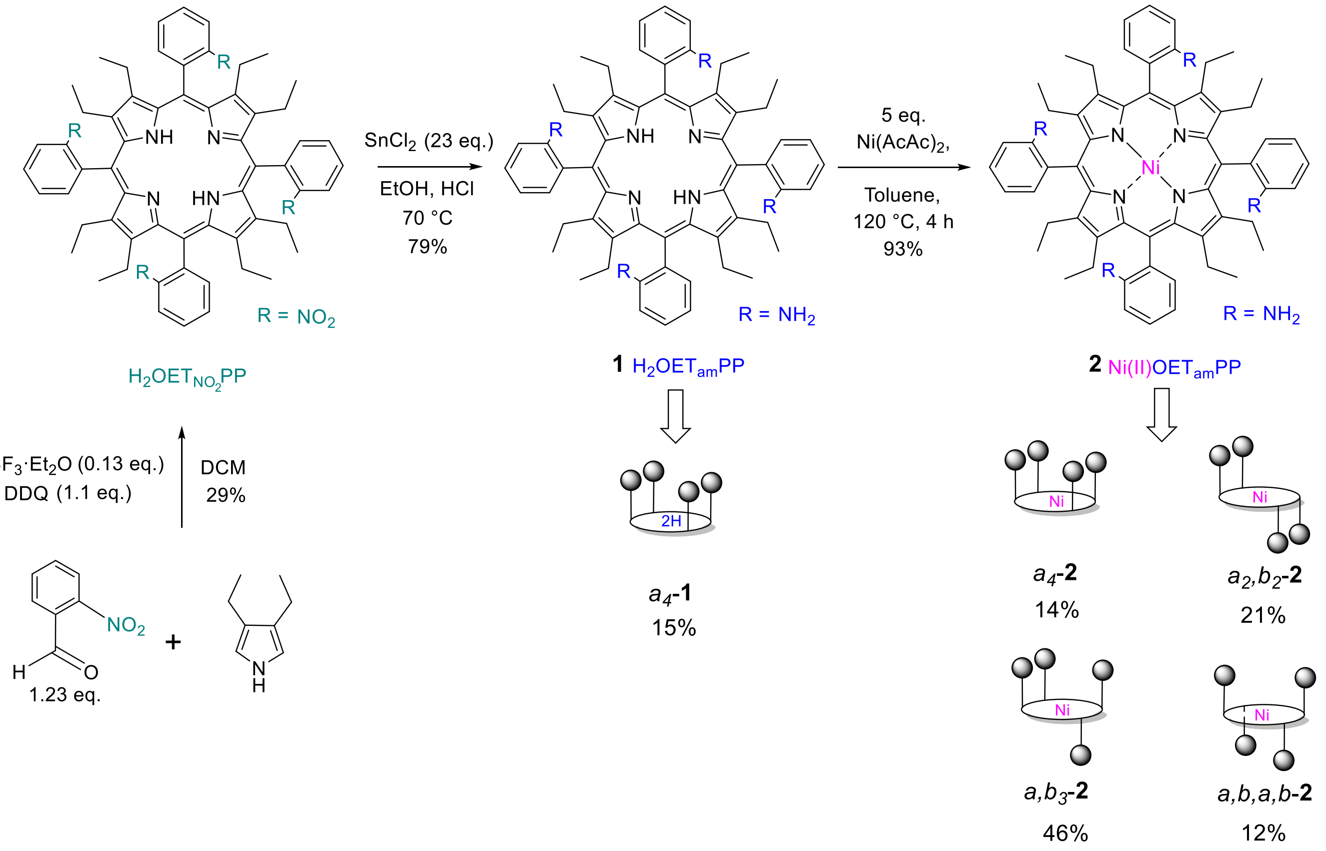


**Scheme S1.** Synthetic scheme for the preparation of *α₄*-**1**, *α,β,α,β*-**2**, *α₂,β₂*-**2**, *α₃,β*-**2**, *α₄*-**2**

*Synthesis and characterization of* ***$H_2OET_{NO_2}PP$***

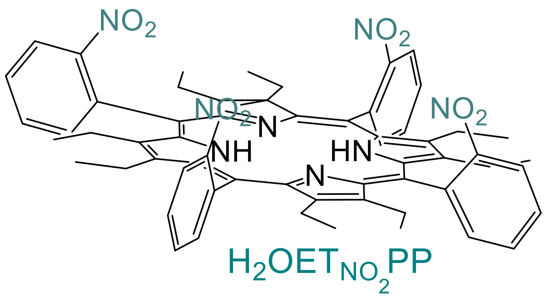


A 2 L Schlenk flask fitted with argon inlet port was filled with 1 L freshly distilled dichloromethane, 2-nitrobenzaldehyde (1.66 g; 11 mmol; 1.23 eq.) and 3,4-diethyl-pyrrole (1.1 g; 8.9 mmol; 1.0 eq.) were added. The solution was stirred at 25 °C under a slow steady stream of argon. After 20 min $BF_3{\cdot}Et_2O$ (150 mg; 1.15 mmol; 0.13 eq.) was added, the reaction vessel was shielded from ambient light with tin foil. After stirring the mixture for 18 h at 25 °C, DDQ (2.3 g; 10.1 mmol; 1.1 eq.) was added into the reaction mixture and stirred for another 1 h. Later, $BF_3{\cdot}Et_2O$ was quenched with TEA (0.14 ml) and the solution was concentrated *via* removing the majority of the solvent at reduced pressure; the residue was transferred directly onto silica gel for column chromatography ($SiO_2$, hexane:ethyl acetate = 3:1, *v/v*). After elution of less polar side products, a mixture of dichloromethane with triethylamine (9:1) was used to elute the major fraction of product **$H_2OET_{NO_2}PP$** (2,3,7,8,12,13,17,18-Octaethyl-5,10,15,20-tetrakis(2-nitrophenyl)porphyrin). Removal of the solvent gave the product as a green solid [0.647 g; 1.27 mmol; 29%]; M.p. >300 °C; $R_f$ = 0.48 ($SiO_2$, ethyl acetate); $^1H$ NMR (600 MHz, *d*-chloroform, 25 °C): $\delta$ = 8.52 (d, $J$ = 6.9 Hz, 4H, Ar–*H*), 8.39 (d, $J$ = 7.5 Hz, 4H, Ar–*H*), 7.96 – 7.86 (m, 8H, Ar–*H*), 2.70 – 1.74 (m, 16H, $CH_2$), 0.59 (t, $J$ = 7.4 Hz, 12H, $CH_3$), 0.48 ppm (t, $J$ = 7.4 Hz, 12H, $CH_3$); $^{13}C$ NMR (100 MHz, *d*-chloroform, 25 °C): $\delta$ =152.95, 138.70, 134.94, 131.33, 129.86, 124.85, 113.19, 20.02, 19.05, 16.55, 15.19 ppm; UV-vis (chloroform): $\lambda_{max}$ (log $\varepsilon$) = 413 (4.44), 468 (4.81), 568 (3.82), 623 (3.78), 719 nm (3.25); MS (MALDI) *m/z* (%) 1018 (100) [$M^+$], 945 (86) [$M^+ - C_5H_{13}$], 945 (86) [$M^+ - C_5H_{13}$], 867 (41) [$M^+ - C_8H_9NO_2$], 254 (33) [$M^+ - C_{45}H_{44}N_6O_6$]; HRMS (MALDI) *m/z* calcd. for $C_{60}H_{60}N_8O_8$ [M]$^+$: 1019.4466, found 1019.4456; IR (ATR): $\tilde{\nu}$ = *2969.0, 2931.0, 2871.5, 1604.1, 1523.8, 1339.0, 1053.12, 749.0, 733.1* cm$^{-1}$.

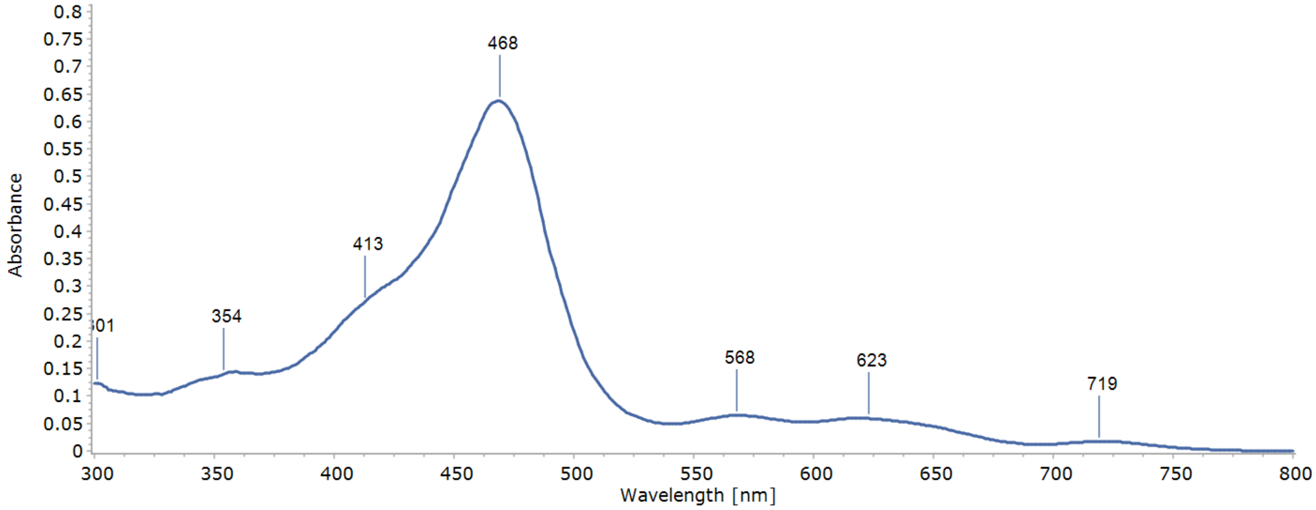


**Figure S1.** UV-vis spectrum of **$H_2OET_{NO_2}PP$.**

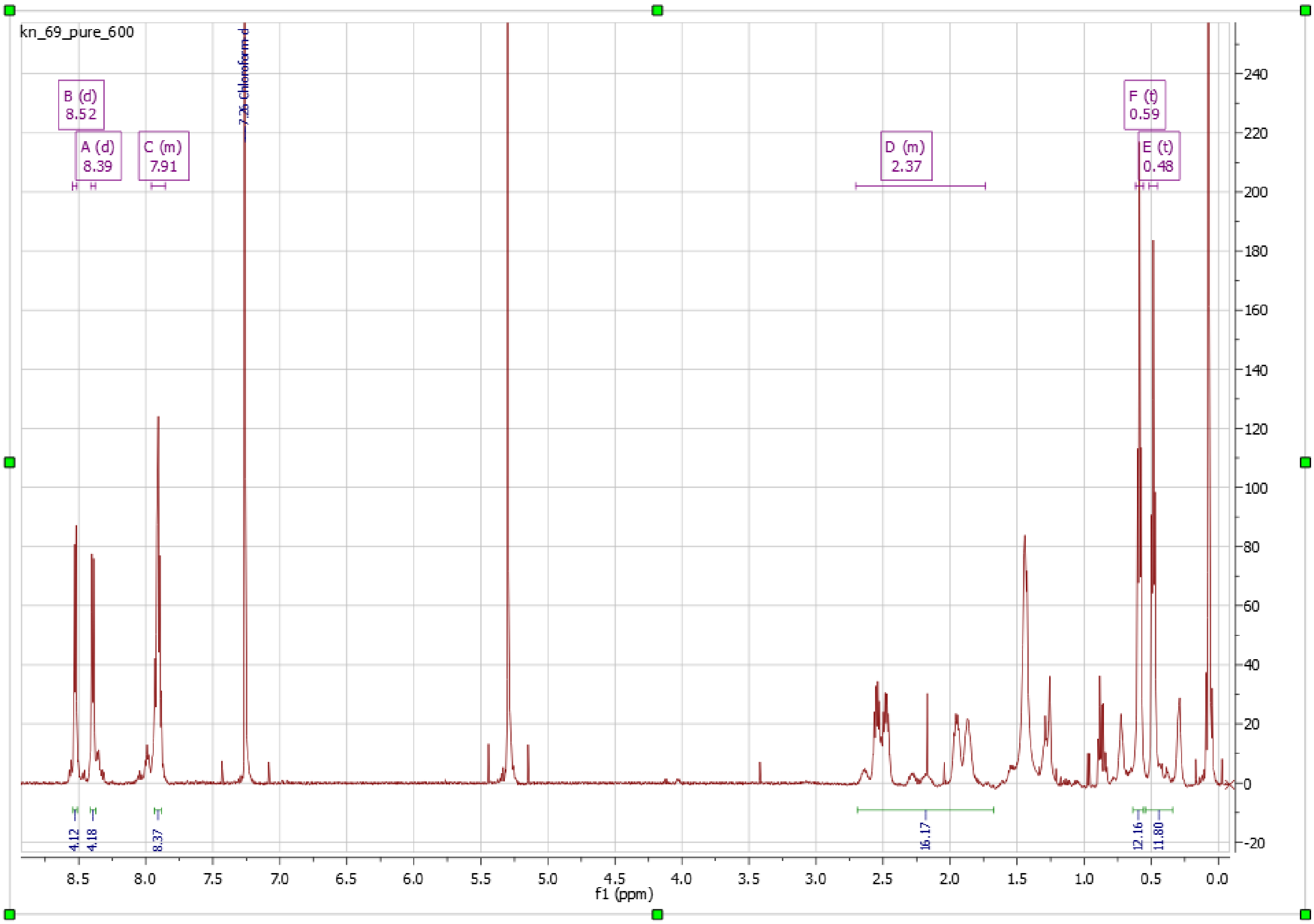


**Figure S2.** $^{1}$H NMR spectrum of **$H_2OET_{NO_2}PP$** (600 MHz, *d*-chloroform, 25 °C).

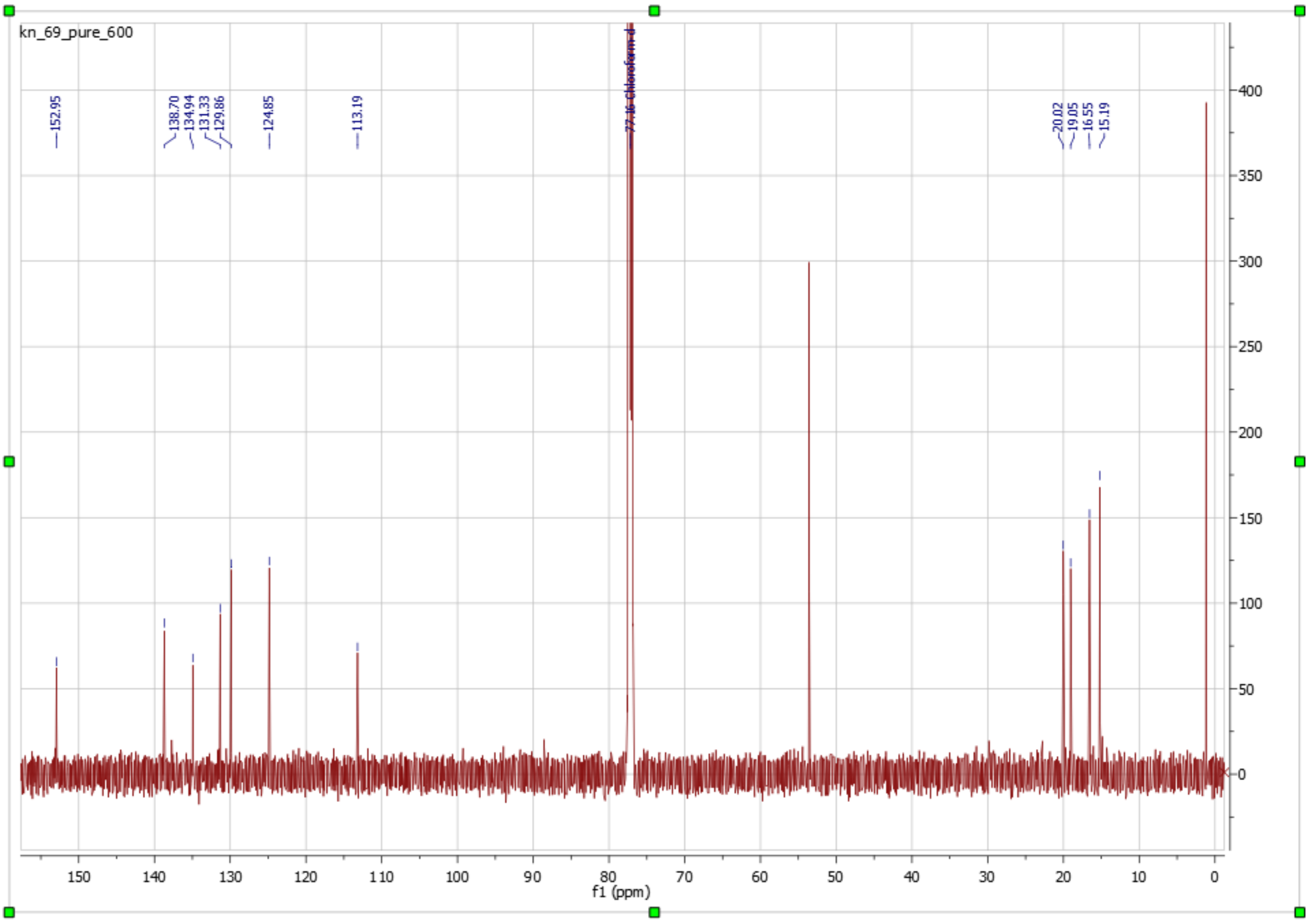


**Figure S3.** $^{13}$C NMR spectrum of **H2OET$_{NO_2}$PP** (100 MHz, *d*-chloroform, 25 °C).

## Elemental Composition Report

Page 1

### Single Mass Analysis

Tolerance = 50.0 PPM / DBE: min = -1.5, max = 1000.0
Element prediction: Off
Number of isotope peaks used for i-FIT = 5

Monoisotopic Mass, Odd and Even Electron Ions
61 formula(e) evaluated with 1 results within limits (up to 10 closest results for each mass)
Elements Used:
C: 0-60 H: 0-60 N: 0-8 O: 0-8
Karolis Norvaisa (MSe), KN_69_2
Q-TOF20171115MF019 26 (0.482) AM (Cen,4, 80.00, Ht,10000.0,1570.68,0.70); Sm (SG, 2x3.00); Sb (15,10.00 ); Cm (9:87)

TOF MS LD+
1.39e+003

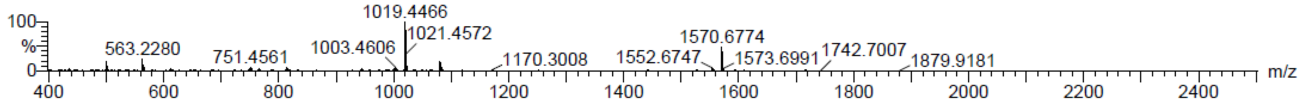


| | | | | | | | |
|---|---|---|---|---|---|---|---|
| Minimum: | | | | -1.5 | | | |
| Maximum: | | 5.0 | 50.0 | 1000.0 | | | |
| Mass | Calc. Mass | mDa | PPM | DBE | i-FIT | i-FIT (Norm) | Formula |
| 1019.4466 | 1019.4456 | 1.0 | 1.0 | 35.5 | 52.4 | 0.0 | C60 H59 N8 O8 |

**Figure S4.** HRMS of **H2OET$_{NO_2}$PP.**

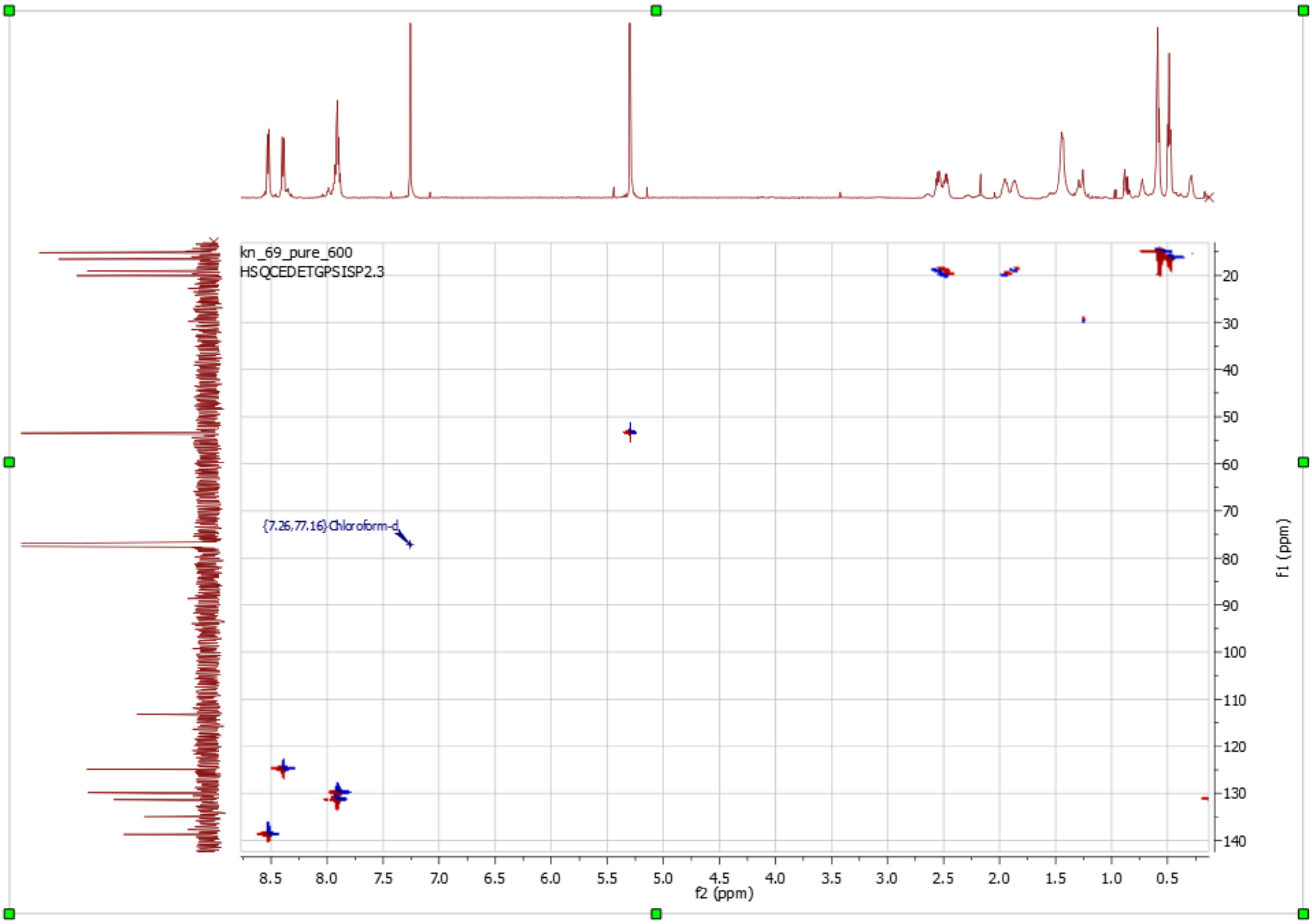


**Figure S5.** $^{1}H$-$^{13}C$ HSQC of **H2OET$_{NO_2}$PP** (600 MHz, *d*-chloroform, 25 °C).

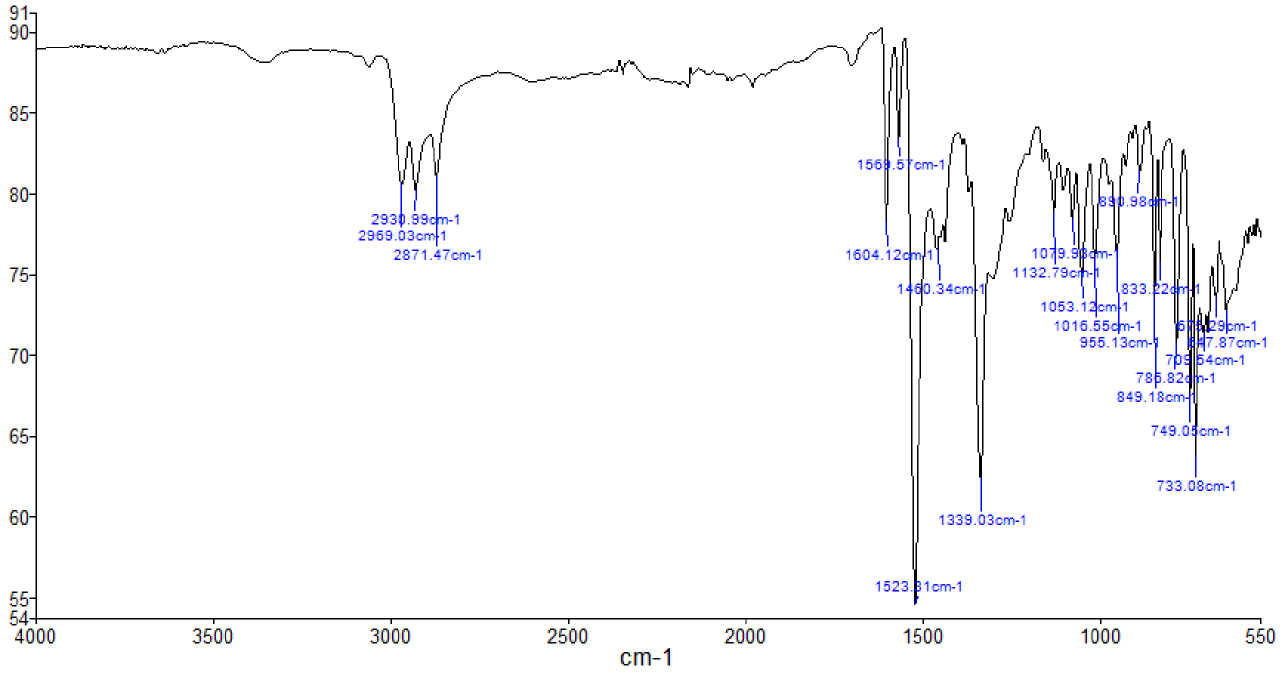


**Figure S6.** FTIR spectrum of **H2OET$_{NO_2}$PP.**

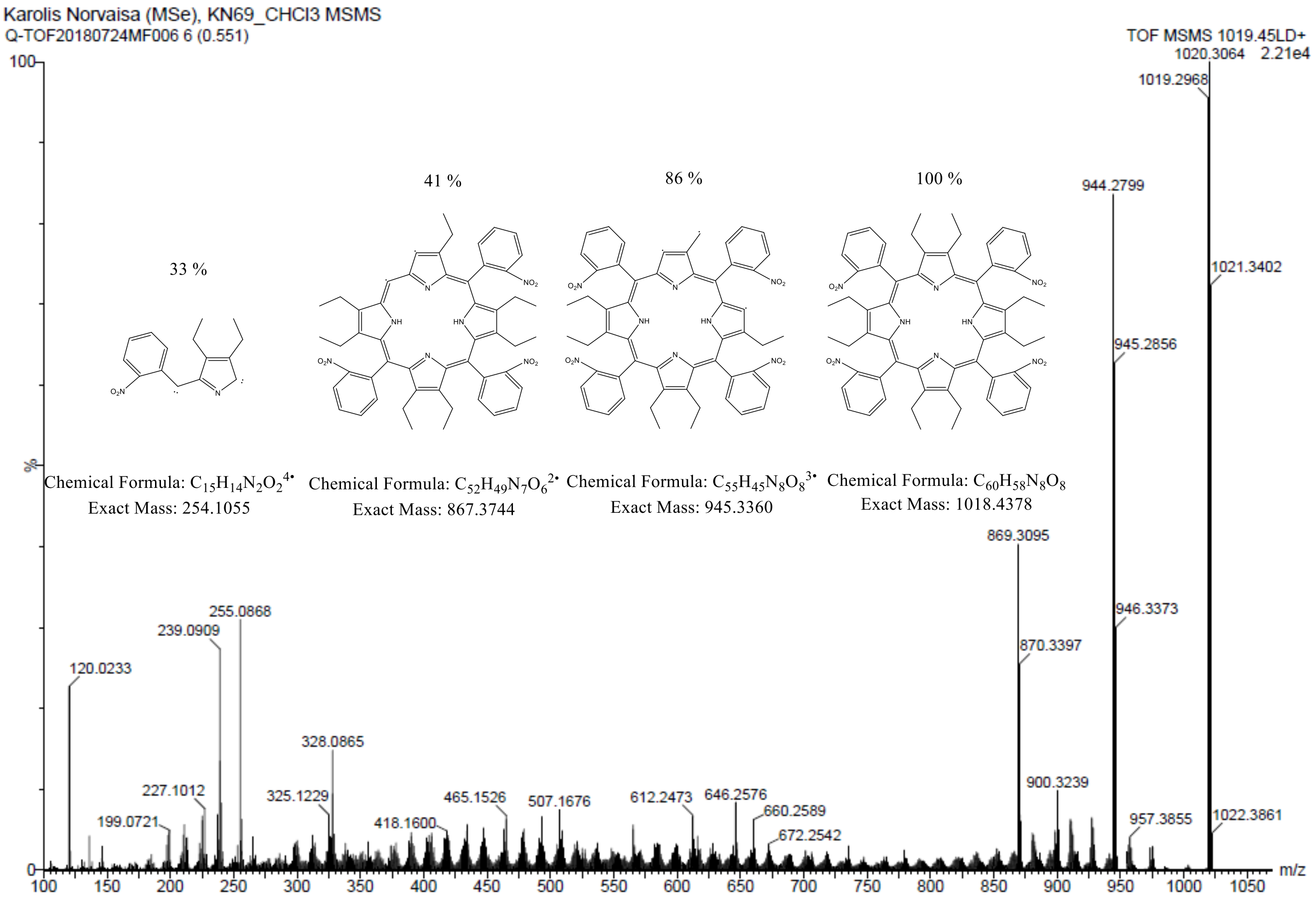


**Figure S7.** MSMS of **H$_2$OET$_{NO_2}$PP.**

*Synthesis and characterization of α$_4$-**1***

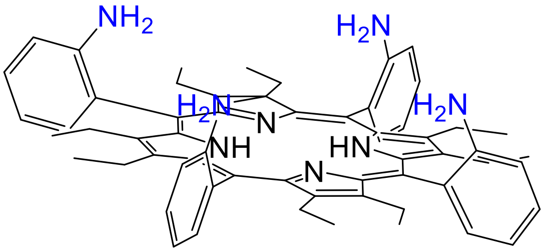


*α*$_4$-**1**

In a 250 mL round bottom flask equipped with water condenser, 2,3,7,8,12,13,17,18-octaethyl-5,10,15,20-tetrakis(2-nitrophenyl)porphyrin (**H$_2$OET$_{NO_2}$PP**, 590 mg; 0.579 mmol; 1 eq.), was dissolved in 15 mL of ethanol at room temperature, followed by addition of excess $SnCl_2$ (2.5 g; 13.18 mmol; 23 eq.) and concentrated hydrochloric acid (25 mL). The resulting green mixture was quickly heated to 80 °C for 1 h, then cautiously quenched with concentrated aqueous ammonia. Chloroform (20 mL) was added to the hot suspension and the mixture was stirred for 1 h. The organic layer was separated and the aqueous layer extracted 3 times with 100 mL batches of dichloromethane. All the organic phases were combined, dried over anhydrous magnesium sulfate, and filtrated. Upon removal of the solvent brown solid as crude atropisomeric mixture of **1** [400.0 mg; 0.444 mmol; 75 %] was isolated. The mixture was dissolved in dichloromethane and purified by column chromatography ($SiO_2$, diethyl ether to DCM:TEA, 100:1, v/v). Last brown band was collected and recrystallized using slow diffusion (dichloromethane:methanol) yielding a dark green solid as product *α$_4$*-**1** (α,α,α,α-5,10,15,20-Tetrakis(2-aminophenyl)-2,3,7,8,12,13,17,18-octaethyl-porphyrin) [76.0 mg; 0.085 mmol; 15 %]. M.p.: >300 °C; *Rf* = 0.2 ($SiO_2$, ethyl acetate); $^1$H NMR (400 MHz, *d*-chloroform, 25 °C): $\delta$ = 8.07 (d, $J$ = 6.9 Hz, 4H, Ar–*H*); 7.52 (t, $J$ = 7.2 Hz, 4H, Ar–*H*), 7.11 (t, $J$ = 7.3 Hz, 4H, Ar–*H*), 6.98 (d, $J$ = 8.0 Hz, 4H, Ar–*H*), 3.96 (s, 8H, N–*H*), 2.88 – 2.73 (m, 4H,-C*H$_2$*), 2.68 – 2.49 (m, 8H,-C*H$_2$*), 2.26 – 2.14 (m, 4H,-C*H$_2$*), 0.72 (t, $J$ = 7.4 Hz, 12H, -*CH$_3$*), 0.50 ppm (t, $J$ = 7.3 Hz, 12H, -*CH$_3$*); $^{13}$C NMR (100 MHz, *d*-chloroform, 25 °C): $\delta$ = 148.84, 142.59, 135.89, 130.33, 125.55, 117.71, 115.29, 113.06, 19.82, 19.03, 17.12, 16.16 ppm; UV/Vis (chloroform): $\lambda_{max}$ (log $\varepsilon$) = 462 (5.12), 559 (4.05), 613 (3.72), 647 (3.61), 711 nm (3.68); MS (MALDI) *m/z* (%) 899 (100) [M$^+$], 884 (82) [M$^+$ – $CH_3$], 870 (49) [M$^+$ – $C_2H_5$], 777 (9) [M$^+$ – $C_8H_{11}N$], 673 (29) [M$^+$ – $C_{15}H_{17}N_2$], 449 (39) [M$^+$ – $C_{30}H_{33}N_4$], 225 (42) [M$^+$ – $C_{45}H_{49}N_6$]; HRMS (MALDI) *m/z* calcd. for $C_{60}H_{67}N_8$ [M]$^+$: 899.5489, found 899.5463; IR (ATR): $\tilde{\nu}$ = *3472.3, 3371.6, 2965.2, 2928.8, 2869.9, 1610.4, 1491.4, 1449.3, 1299.0, 1255.4, 1156.2, 1016.4, 955.4, 745.5, 724.1* cm$^{-1}$.

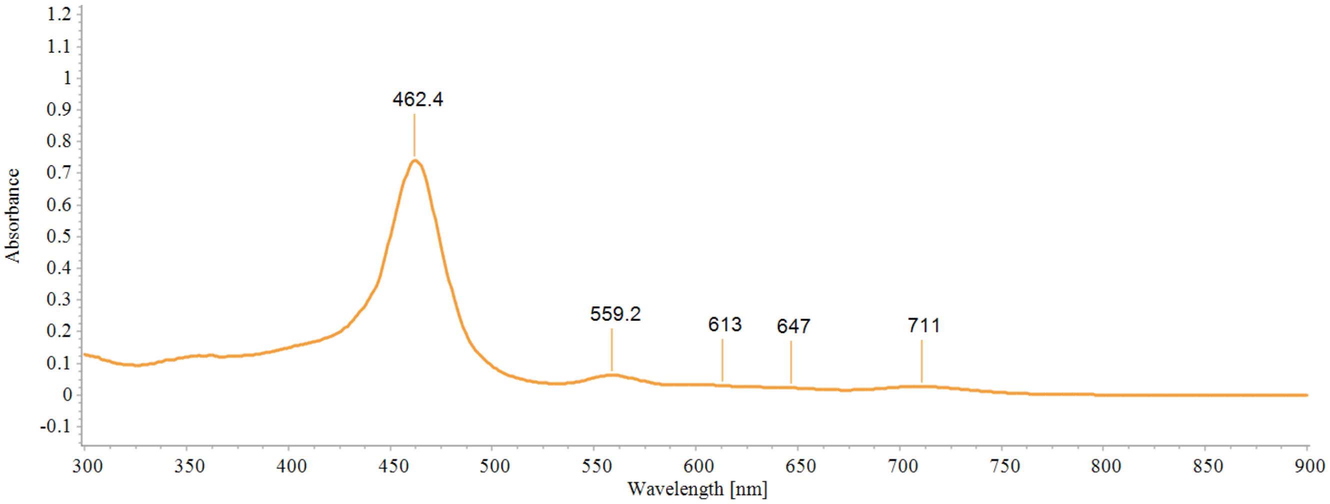


**Figure S8.** UV-vis spectrum of *α₄*-**1** in chloroform.

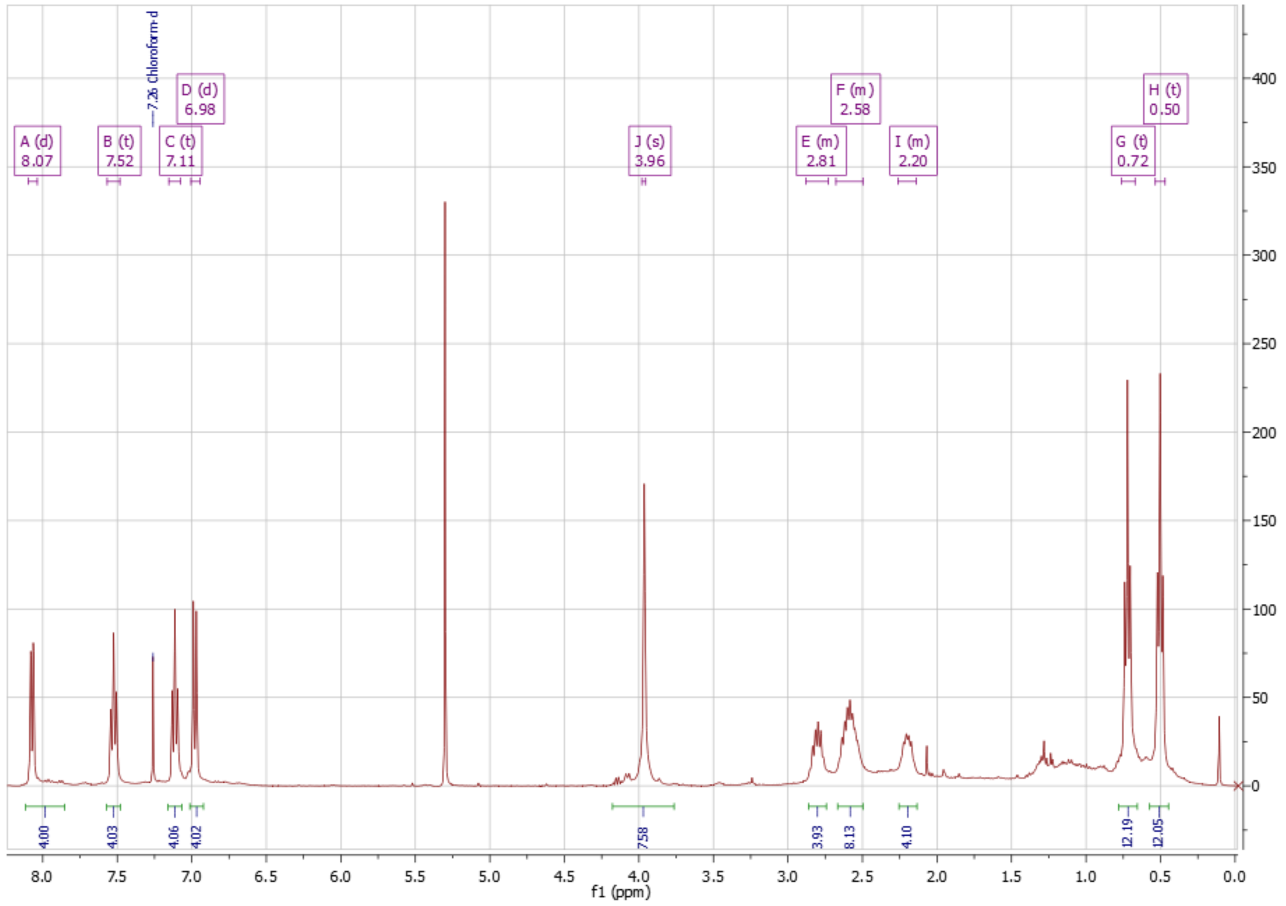


**Figure S9.** $^{1}$H NMR spectrum of *α₄*-**1** (400 MHz, *d*-chloroform, 25 °C).

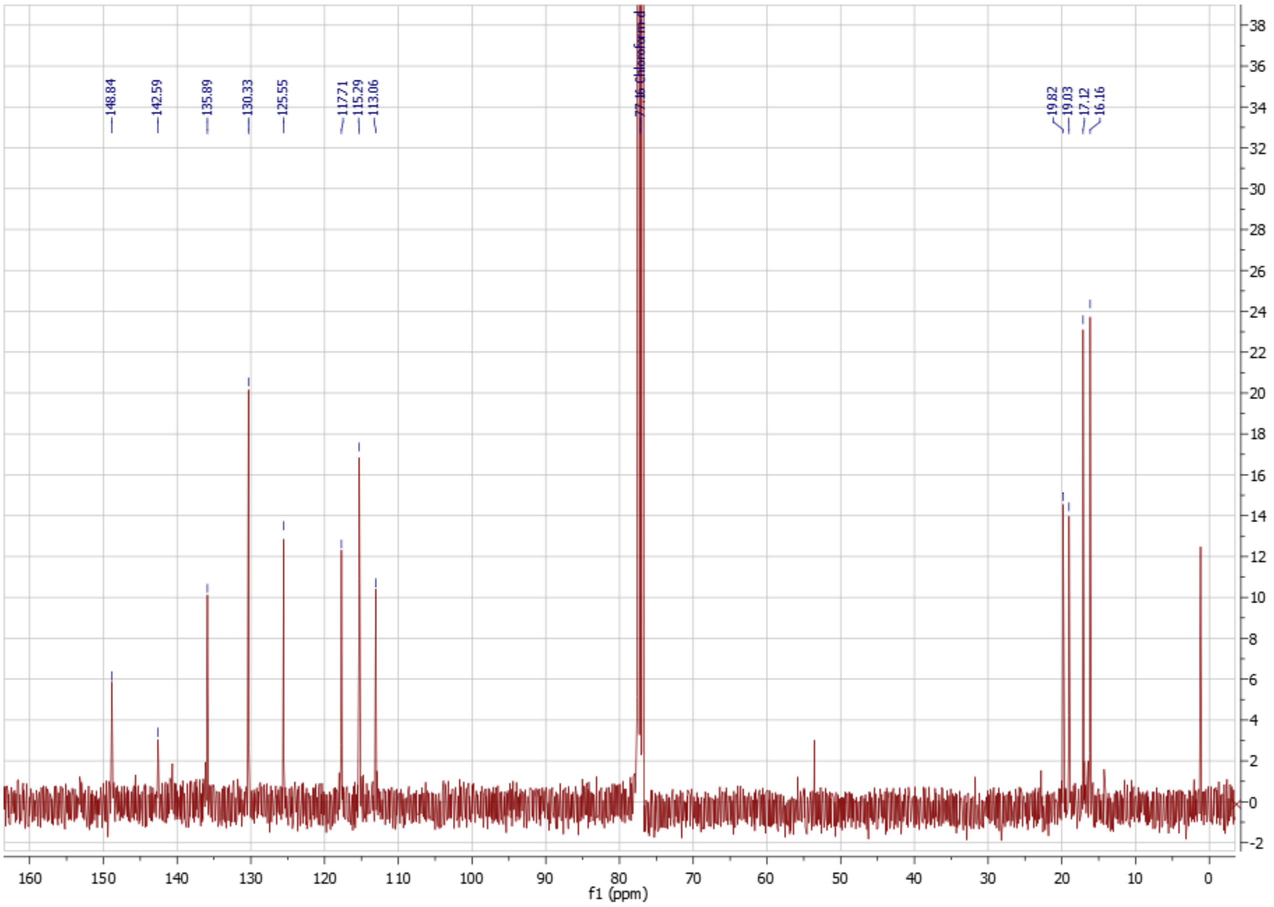


**Figure S10.** $^{13}$C NMR spectrum of *α₄*-**1** (100 MHz, *d*-chloroform, 25 °C).

## Elemental Composition Report

Page 1

### Single Mass Analysis

Tolerance = 50.0 PPM / DBE: min = -1.5, max = 400.0
Element prediction: Off
Number of isotope peaks used for i-FIT = 5

Monoisotopic Mass, Odd and Even Electron Ions
5 formula(e) evaluated with 1 results within limits (up to 10 best isotopic matches for each mass)
Elements Used:
C: 0-60 H: 0-67 N: 0-8
Karolis Norvaisa (MSe), KN70_CH3Cl3
Q-TOF20180719MF014 47 (1.053) AM (Cen,6, 80.00, Ht,10000.0,1570.68,0.70); Sm (SG, 2x3.00); Sb (15,10.00 ); Cm (7:77-46:48) TOF MS LD+ 1.83e+003

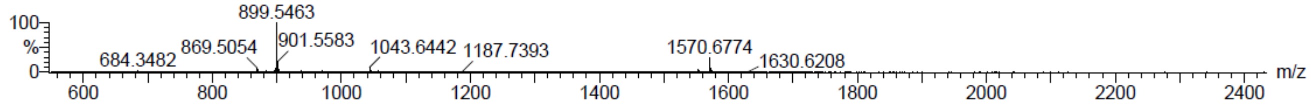


| Mass | Calc. Mass | mDa | PPM | DBE | i-FIT | i-FIT (Norm) | Formula |
|---|---|---|---|---|---|---|---|
| Minimum: | | | | -1.5 | | | |
| Maximum: | | 5.0 | 50.0 | 400.0 | | | |
| 899.5463 | 899.5489 | -2.6 | -2.9 | 31.5 | 74.8 | 0.0 | C60 H67 N8 |

**Figure S11.** HRMS of *α₄*-**1**

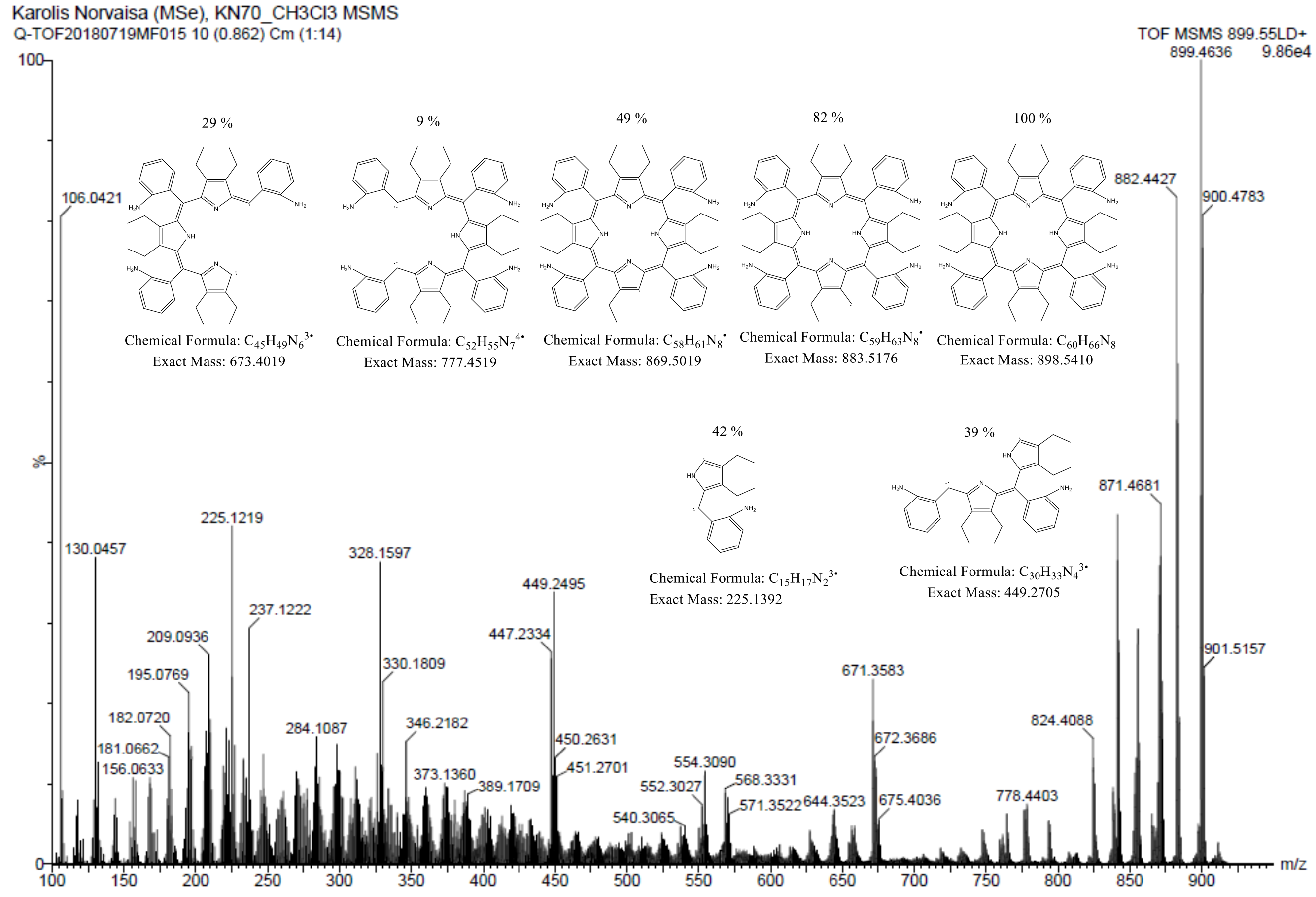


**Figure S12.** MSMS spectrum of *α4*-**1.**

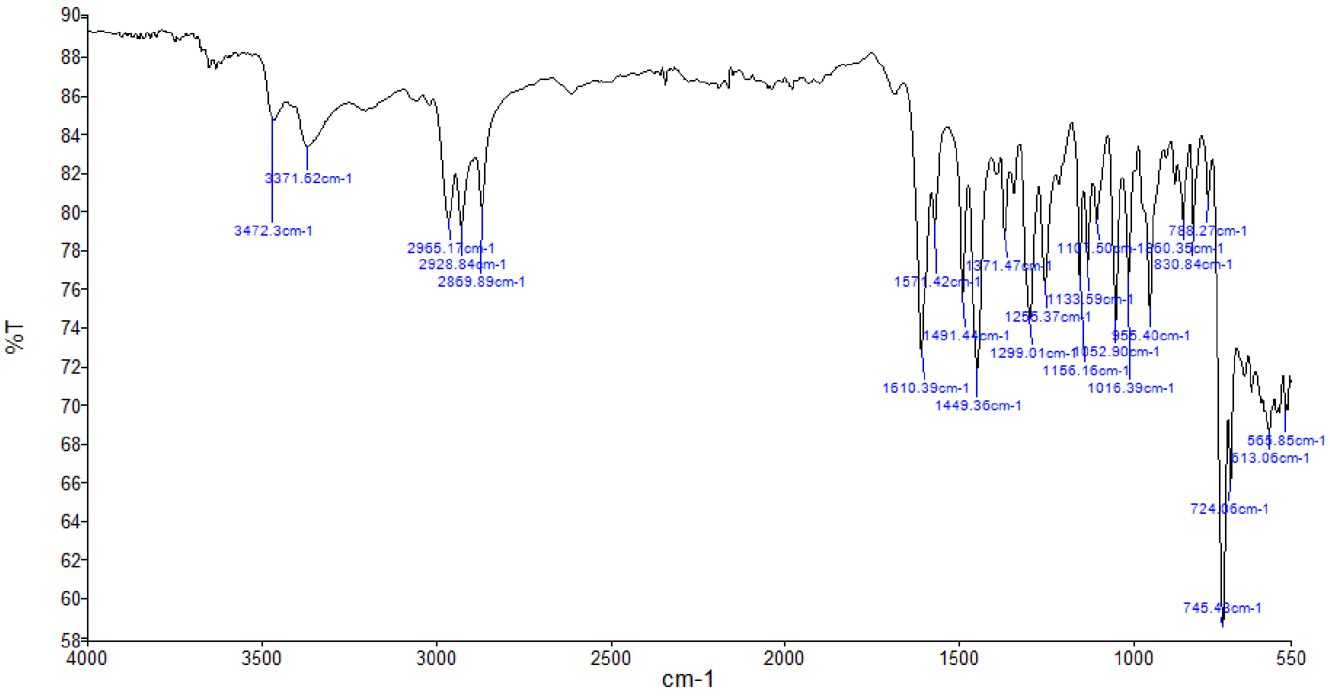


**Figure S13.** FTIR spectrum of *α4*-**1.**

# SUPPORTING INFORMATION

*Synthesis and characterization of α,β,α,β-**2***

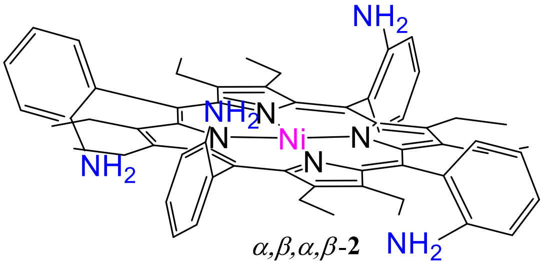


*α,β,α,β*-**2**

In a 500 mL round bottom flask equipped with water condenser the atropisomeric mixture of 5,10,15,20-tetrakis(2-aminophenyl)-2,3,7,8,12,13,17,18-octaethylporphyrin (**1**, 685 mg; 0.762 mmol; 1 eq.), was dissolved in 120 mL of toluene at room temperature, followed by addition of excess nickel(II) acetylacetonate (0.891 g; 3.84 mmol; 5 eq.). The resulting brown mixture was quickly heated to 120 °C for 4 h. Upon removal of the solvent under reduced pressure, the purple solid was dissolved in dichloromethane and transferred to silica gel for column chromatography ($SiO_2$, dichloromethane). First brown/red band was collected and recrystallized using slow diffusion (chloroform:methanol) giving *α,β,α,β*-**2** ([α,β,α,β-5,10,15,20-Tetrakis(2-aminophenyl)-2,3,7,8,12,13,17,18-octaethylporphyrinato]nickel(II)) as purple platy habit crystals [88.0 mg; 0.092 mmol; 12 %]. M.p.: >300 °C; $R_f$ = 0.96 ($SiO_2$, dichloromethane); $^1$H NMR (400 MHz, *d*-chloroform, 25 °C): $\delta$ = 7.58 (d, $J$ = 7.4 Hz, 4H, Ar–*H*), 7.45 (t, $J$ = 7.7 Hz, 4H, Ar–*H*), 7.00 – 6.92 (m, 8H, Ar–*H*), 3.92 (s, 8H, N–*H*), 2.62 – 2.37 (m, 16H,-C*H*2), 0.63 (t, $J$ = 7.3 Hz, 24H, -C*H3*); $^{13}$C NMR (100 MHz, *d*-chloroform, 25 °C): $\delta$ = 147.29, 145.58, 144.72, 135.39, 130.10, 124.85, 118.05, 114.73, 112.40, 19.60, 16.72 ppm; UV/Vis (chloroform): $\lambda_{max}$ (log $\varepsilon$) = 439 (5.08), 559 (3.99), 596 nm (3.67); MS (MALDI) *m/z* (%) 955 (100) [M$^+$], 899 (55) [M$^+$ – Ni], 777 (8) [M$^+$ – $NiC_8H_{13}N$], 762 (10) [M$^+$ – $NiC_9H_{14}N$], 673 (5) [M$^+$ – $NiC_{15}H_{17}N_2$]; HRMS (MALDI) *m/z* calc. for $C_{60}H_{67}N_8$ [M]$^+$: 954.4607, found 954.4585; IR (ATR): $\tilde{\nu}$ = *3472.9, 3380.7, 2959.2, 2925.1, 2868.3, 1609.1, 1449.1, 1296.7, 1256.1, 1156.7, 1021.2, 991.2, 747.0, 727.1* cm$^{-1}$.

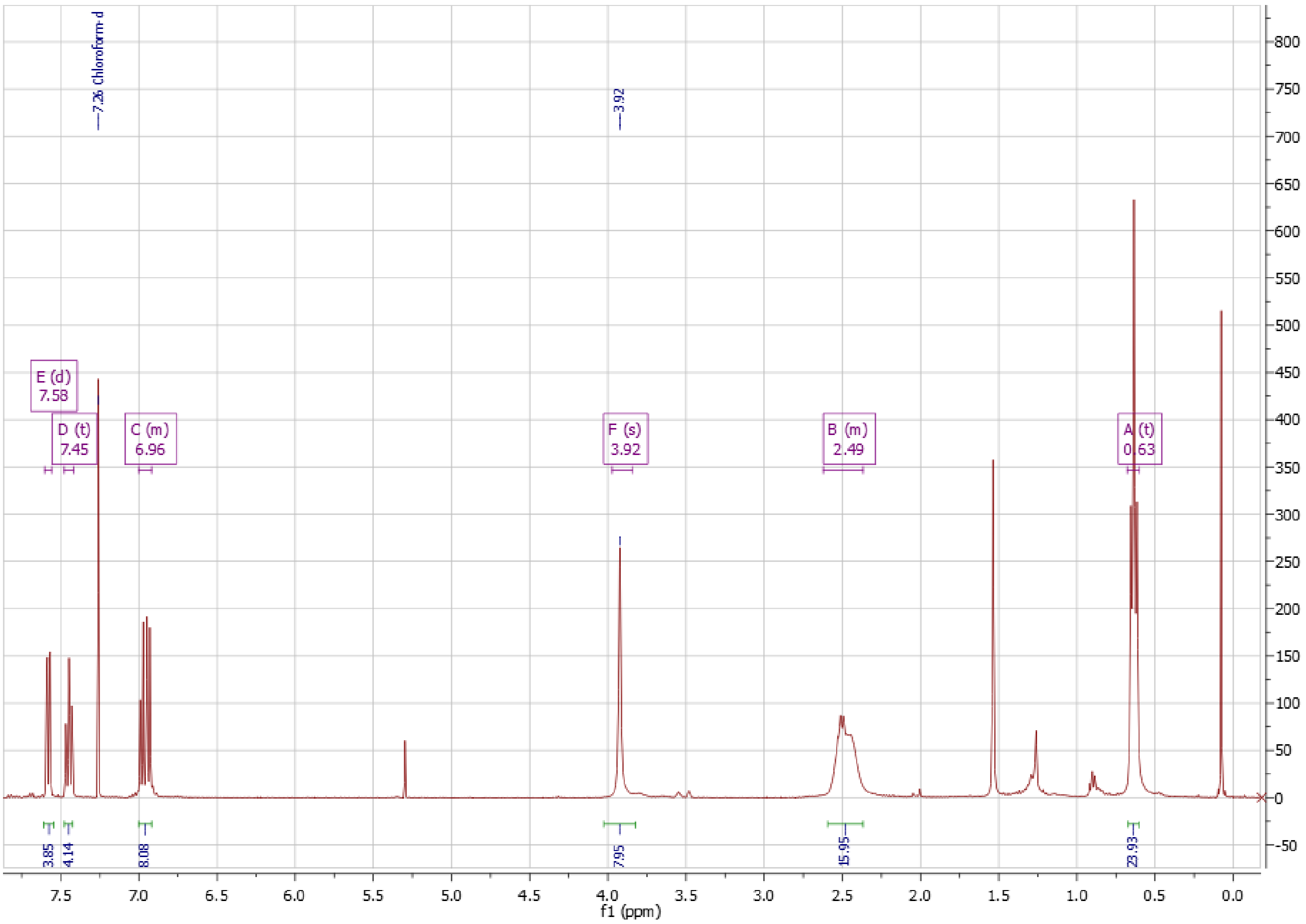


**Figure S14.** $^1$H NMR spectrum of *α,β,α,β*-**2** (400 MHz, *d*-chloroform, 25 °C).

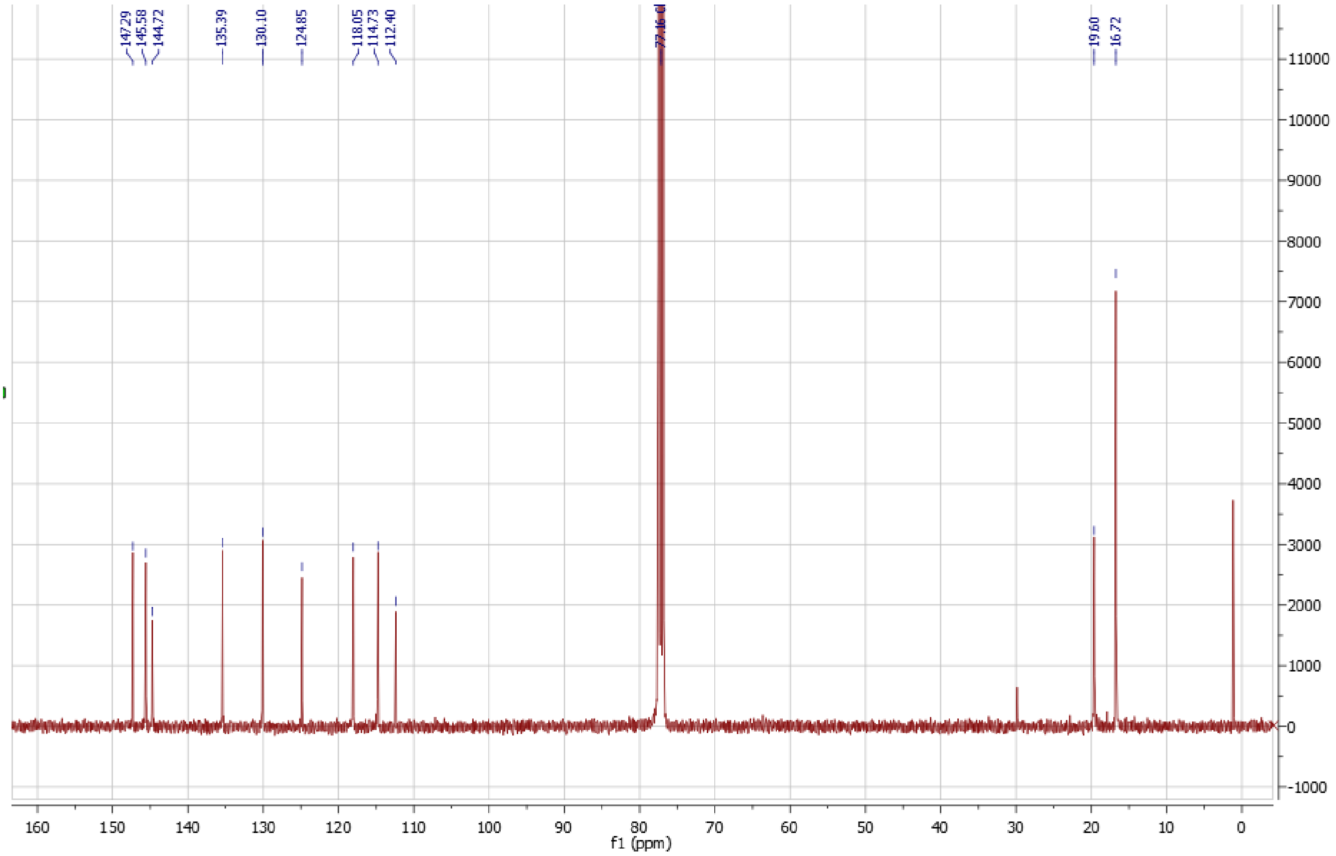


**Figure S15.** $^{13}C$ NMR spectrum of *α,β,α,β*-**2** (100 MHz, *d*-chloroform, 25 °C).

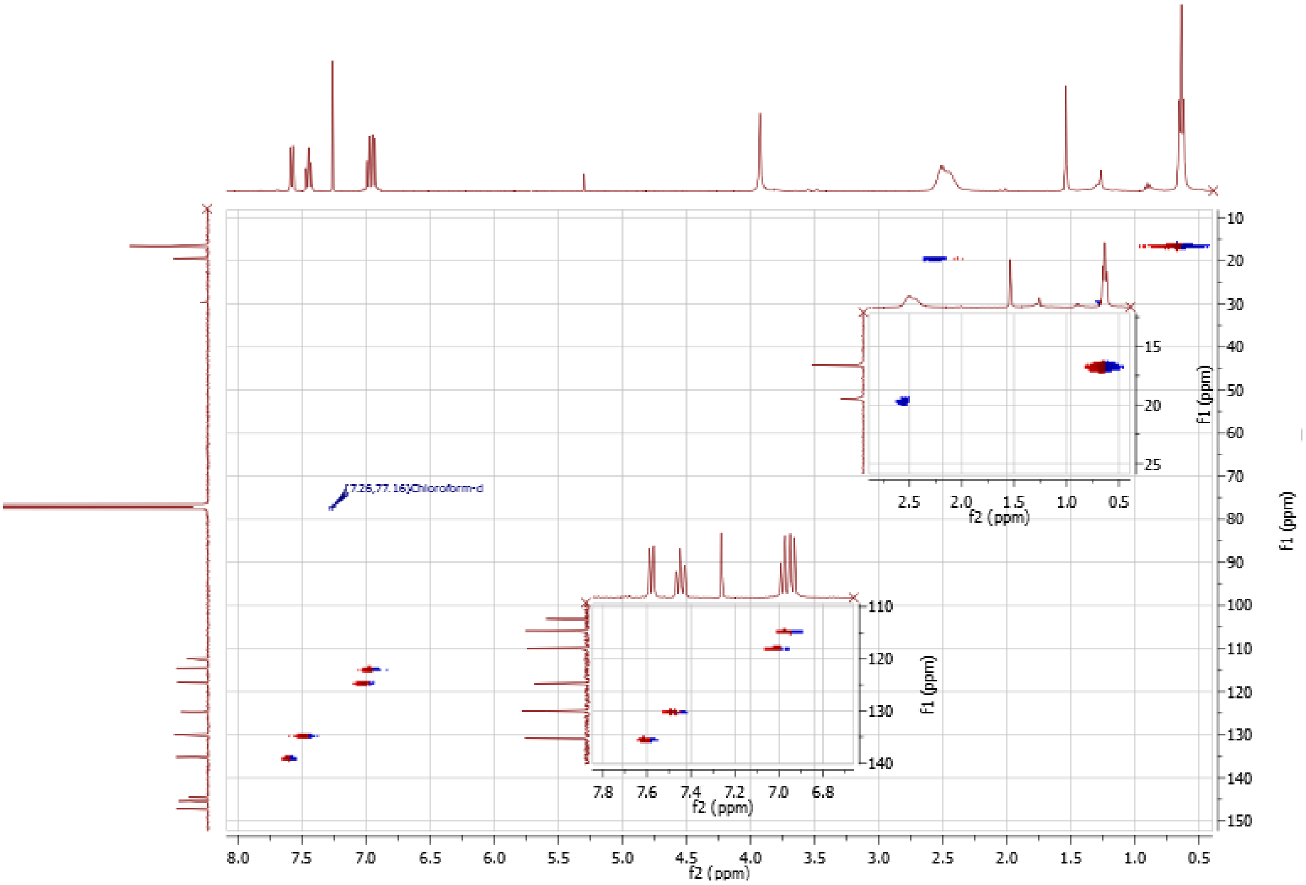


**Figure S16.** $^{1}H$-$^{13}C$ HSQC of *α,β,α,β*-**2** (400 MHz, *d*-chloroform, 25 °C).

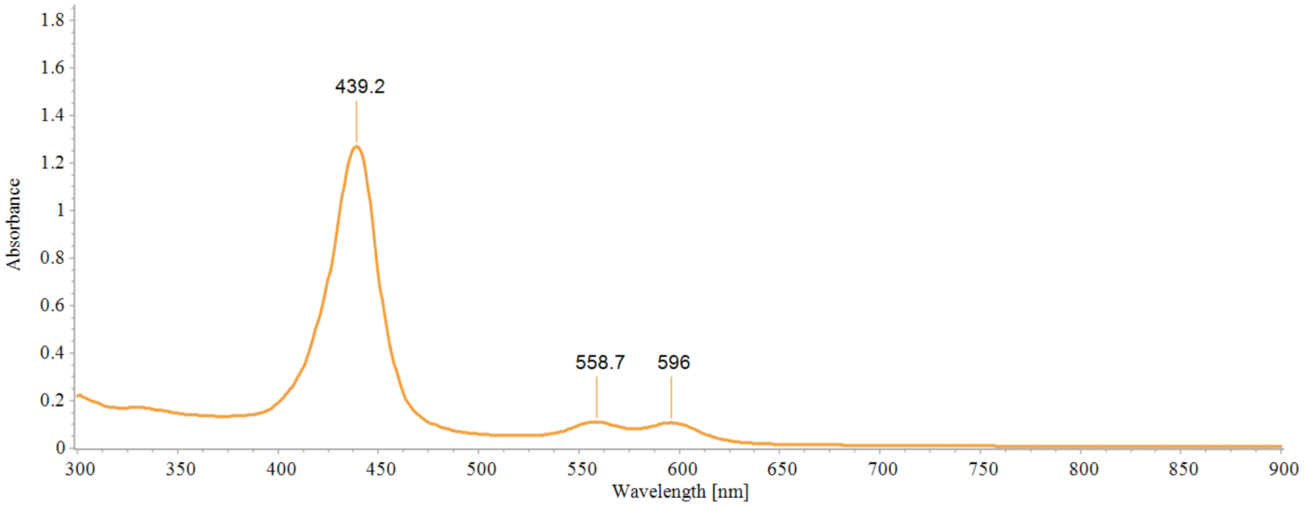


**Figure S17.** UV-vis spectrum of *α,β,α,β*-**2** in chloroform.

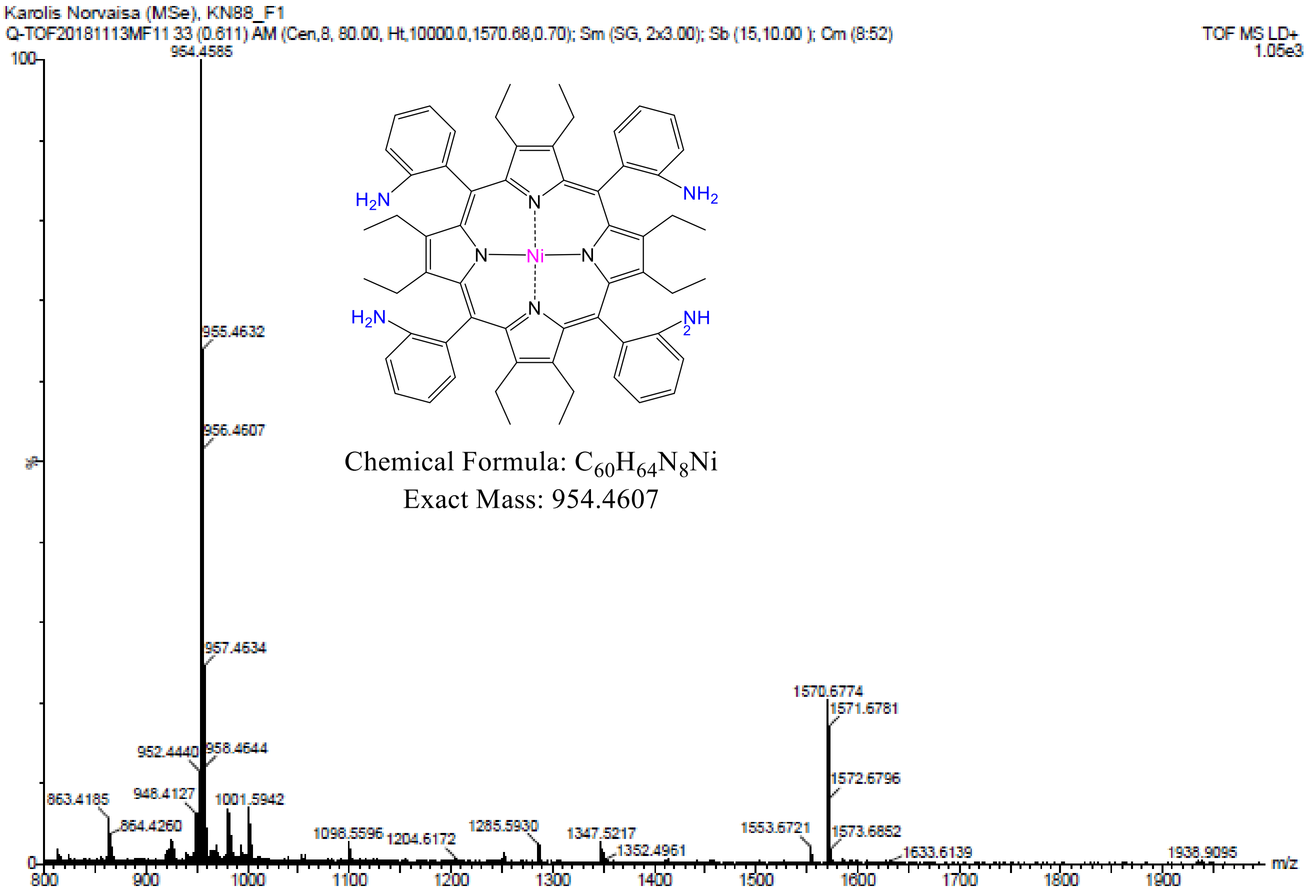


**Figure S18.** HRMS of *α,β,α,β*-**2**.

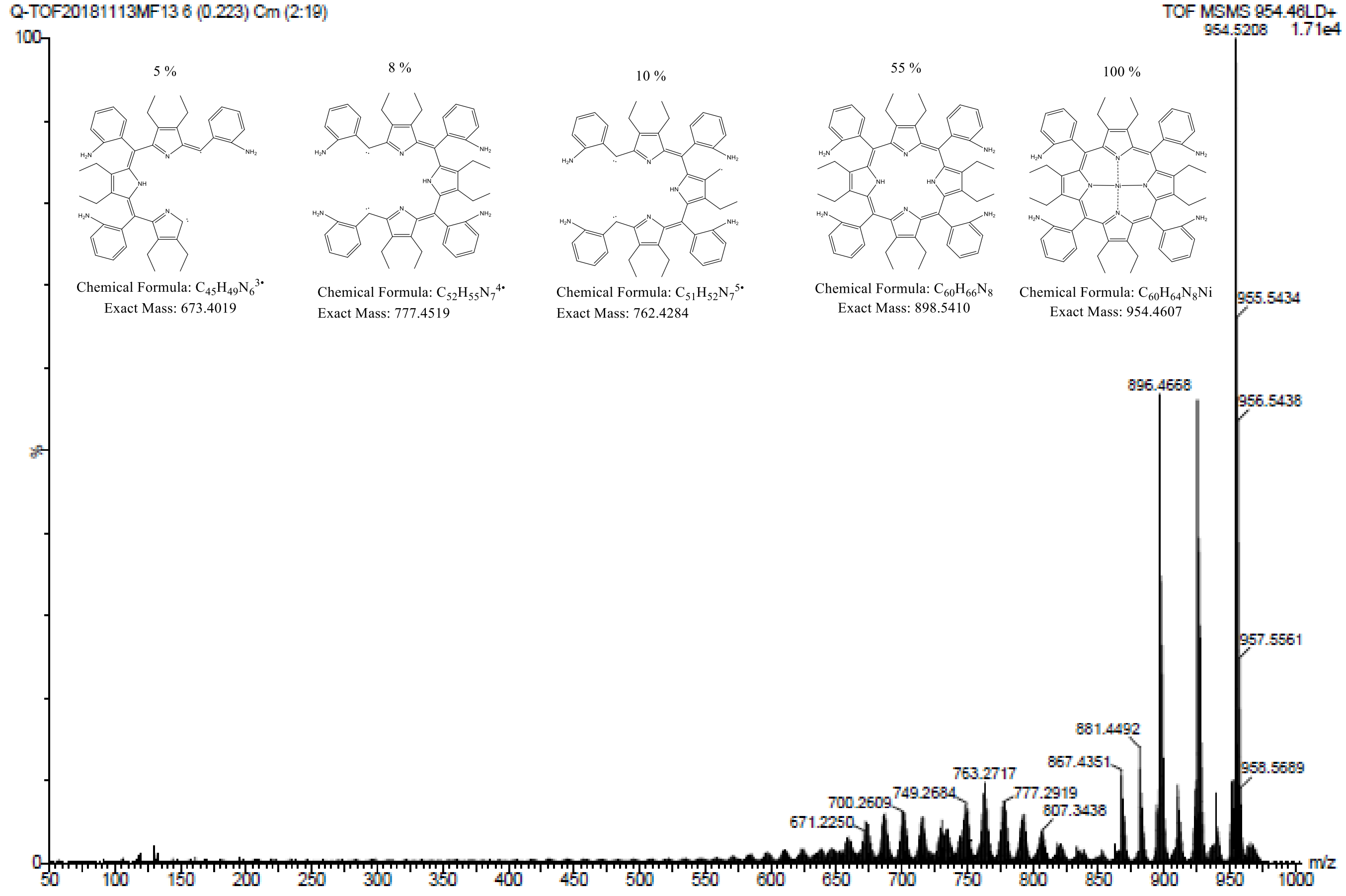


**Figure S19.** MSMS of *α,β,α,β*-**2**.

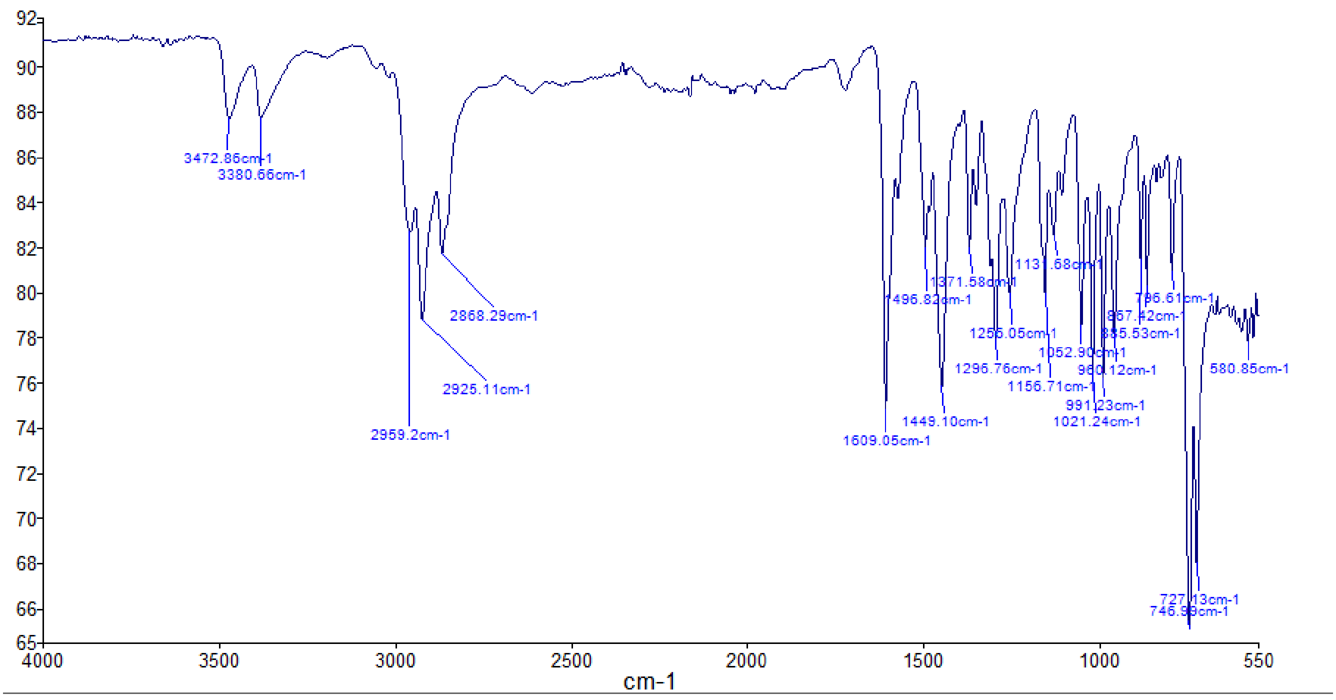


**Figure S20.** FTIR spectrum of *α,β,α,β*-**2**.

# SUPPORTING INFORMATION

*Synthesis and characterization of α2,β2-**2***

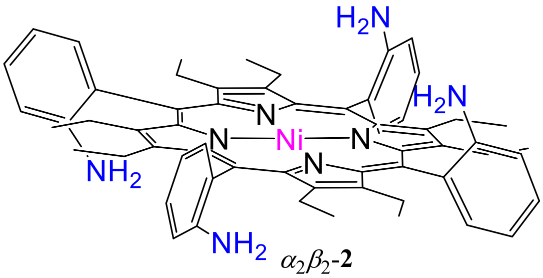

$\alpha_2\beta_2$-**2**

In a 500 mL round bottom flask equipped with water condenser the atropisomeric mixture of 5,10,15,20-tetrakis(2-aminophenyl)-2,3,7,8,12,13,17,18-octaethylporphyrin (**1**, 685 mg; 0.762 mmol; 1 eq.), was dissolved in 120 mL of toluene at room temperature, followed by addition of excess nickel(II) acetylacetonate (0.891 g; 3.84 mmol; 5 eq.). The resulting brown mixture was quickly heated to 120 °C for 4 h. Upon removal of the solvent under reduced pressure, the purple solid was dissolved in dichloromethane and transferred to silica gel for column chromatography ($SiO_2$, dichloromethane). Second brown/red band was collected and recrystallized using slow diffusion (chloroform:methanol) giving *α2,β2*-**2** ([α,α,β,β-5,10,15,20-Tetrakis(2-aminophenyl)-2,3,7,8,12,13,17,18-octaethylporphyrinato]nickel(II)) as purple platy habit crystals [156.0 mg; 0.163 mmol; 21 %]. M.p.: >300 °C; $R_f$ = 0.84 ($SiO_2$, dichloromethane); $^1$H NMR (400 MHz, *d*-chloroform, 25 °C): *δ* = 7.72 (d, *J* = 7.4 Hz, 4H, Ar–*H*), 7.45 (t, *J* = 7.7 Hz, 4H, Ar–*H*), 7.01 (t, *J* = 7.4 Hz, 4H, Ar–*H*), 6.91 (d, *J* = 8.0 Hz, 4H, Ar–*H*), 3.75 (s, 8H, N–*H*), 2.71 – 2.24 (m, 16H,-C*H2*), 0.63 (t, *J* = 7.3 Hz, 24H -C*H3*); $^{13}$C NMR (100 MHz, *d*-chloroform, 25 °C): *δ* = 147.36, 145.57, 144.73, 135.36, 130.09, 124.84, 118.09, 114.88, 112.35, 19.64, 16.76 ppm; UV/Vis (chloroform): $\lambda_{max}$ (log *ε*) = 439 (5.28), 560 (4.16), 599 nm (4.20); MS (MALDI) *m/z* (%) 955 (100) [M$^+$], 899 (50) [M$^+$ – Ni], 777 (8) [M$^+$ – $NiC_8H_{13}N$], 762 (10) [M$^+$ – $NiC_9H_{14}N$], 673 (5) [M$^+$ – $NiC_{15}H_{17}N_2$]; HRMS (MALDI) *m/z* calc. for $C_{60}H_{67}N_8$ [M]$^+$: 954.4607, found 954.4601; IR (ATR): $\tilde{\nu}$ = *3464.7, 3372.0, 2968.1, 2927.2, 2869.2, 1609.0, 1448.7, 1296.4, 1256.1, 1157.5, 1053.0, 1222.0, 990.3, 749.3, 727.3* cm$^{-1}$.

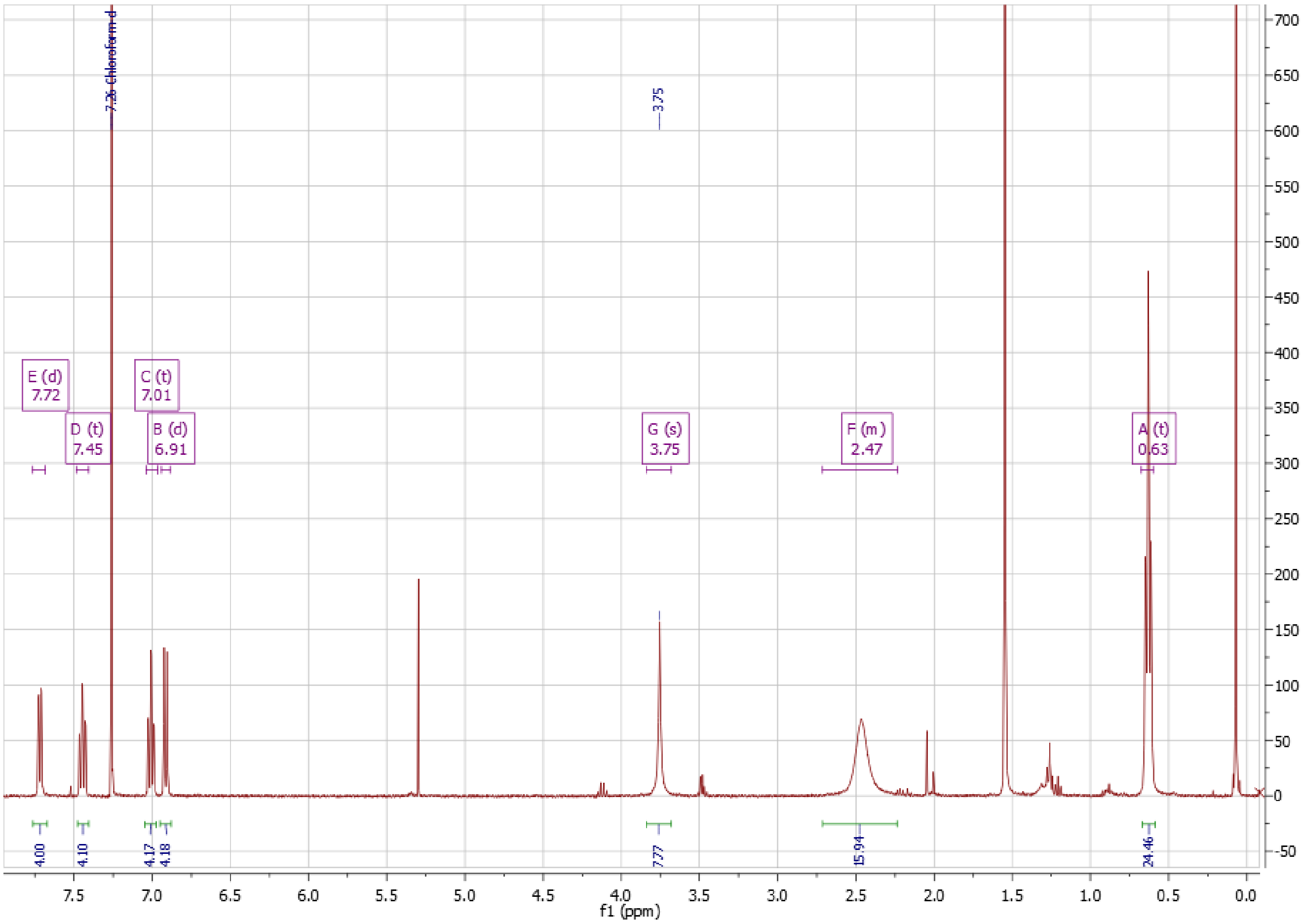


**Figure S21.** $^1$H NMR spectrum of *α2,β2*-**2** (400 MHz, *d*-chloroform, 25 °C).

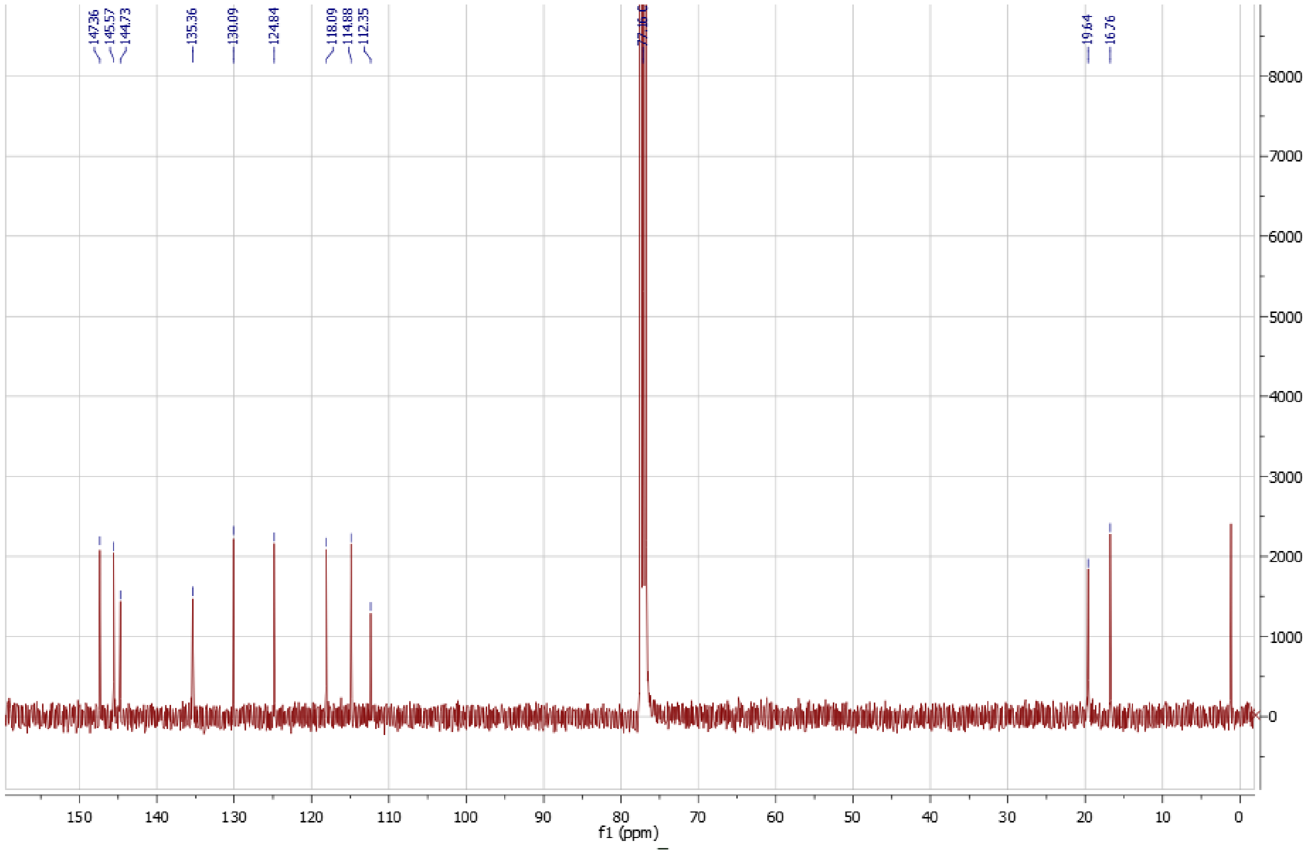

**Figure S22.** $^{13}$C NMR spectrum of $\alpha_2,\beta_2$-**2** (100 MHz, *d*-chloroform, 25 °C).

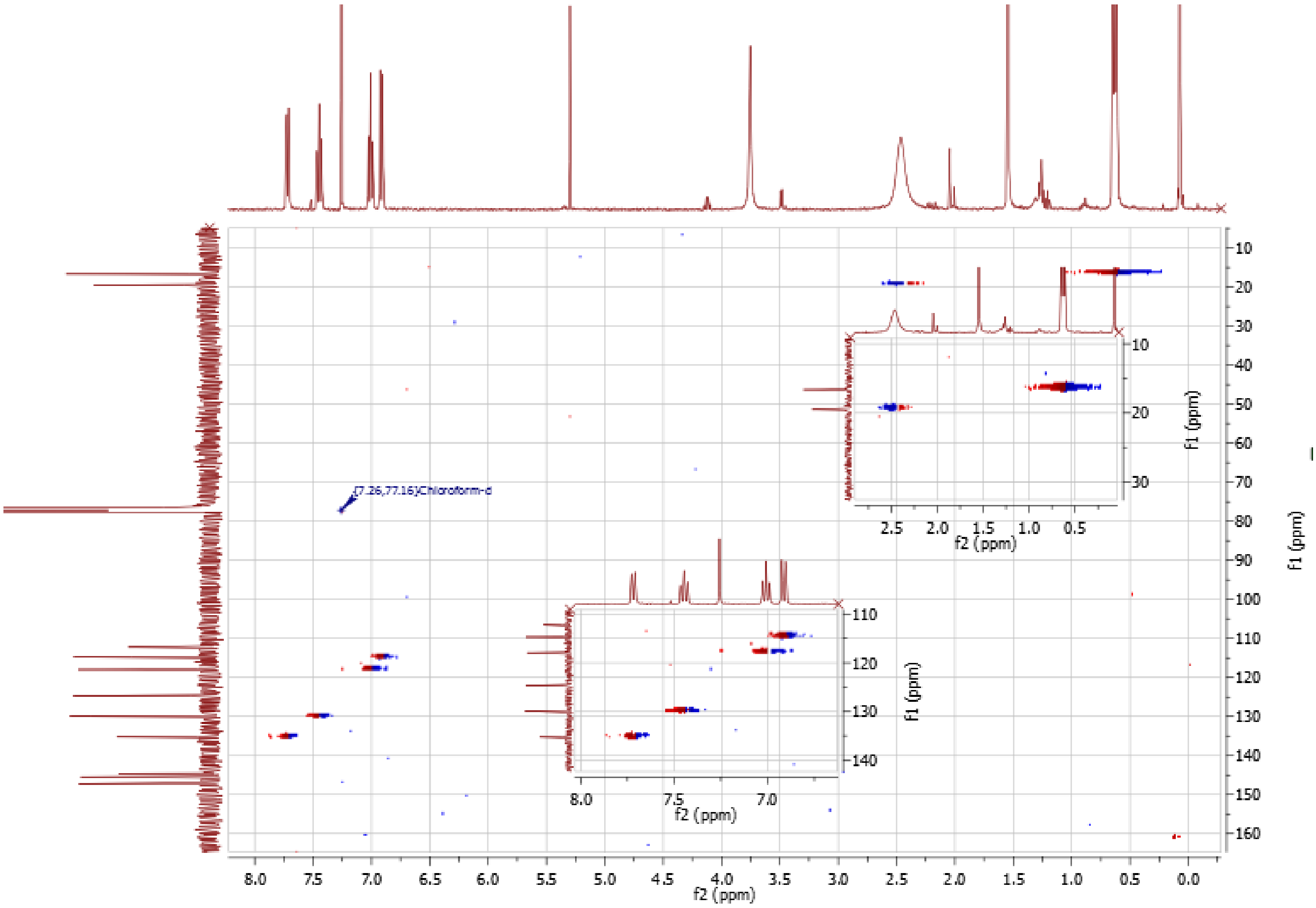

**Figure S23.** $^{1}$H-$^{13}$C HSQC of $\alpha_2,\beta_2$-**2** (400 MHz, *d*-chloroform, 25 °C).

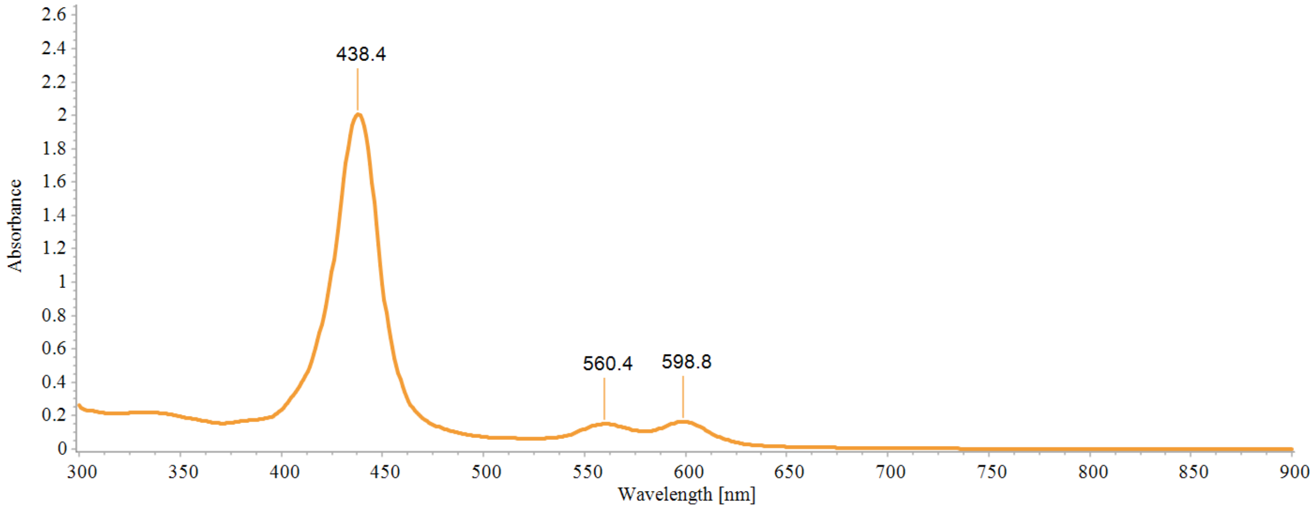


**Figure S24.** UV-vis spectrum of *α*$_2$,*β*$_2$-**2** in chloroform.

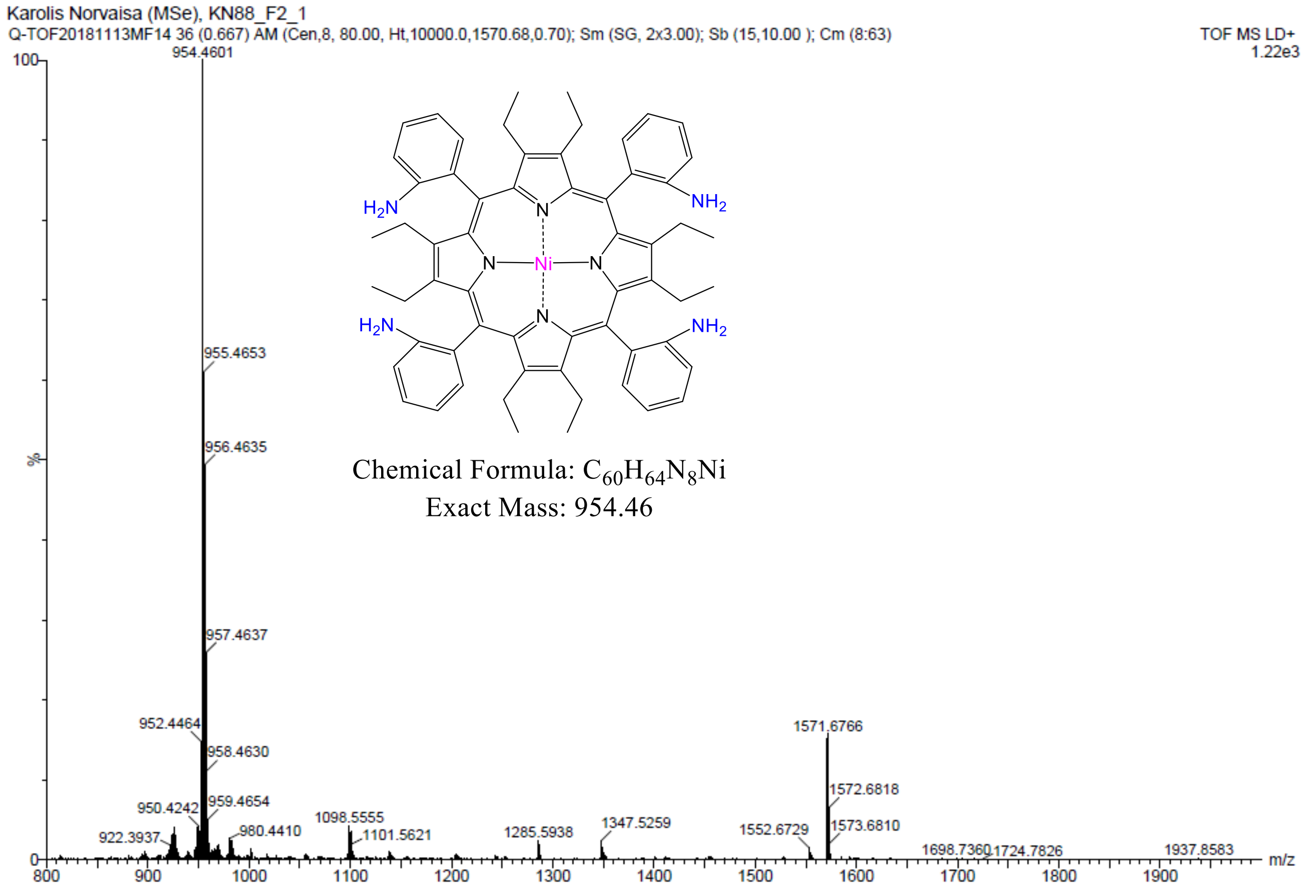


**Figure S25.** HRMS of *α*$_2$,*β*$_2$-**2**.

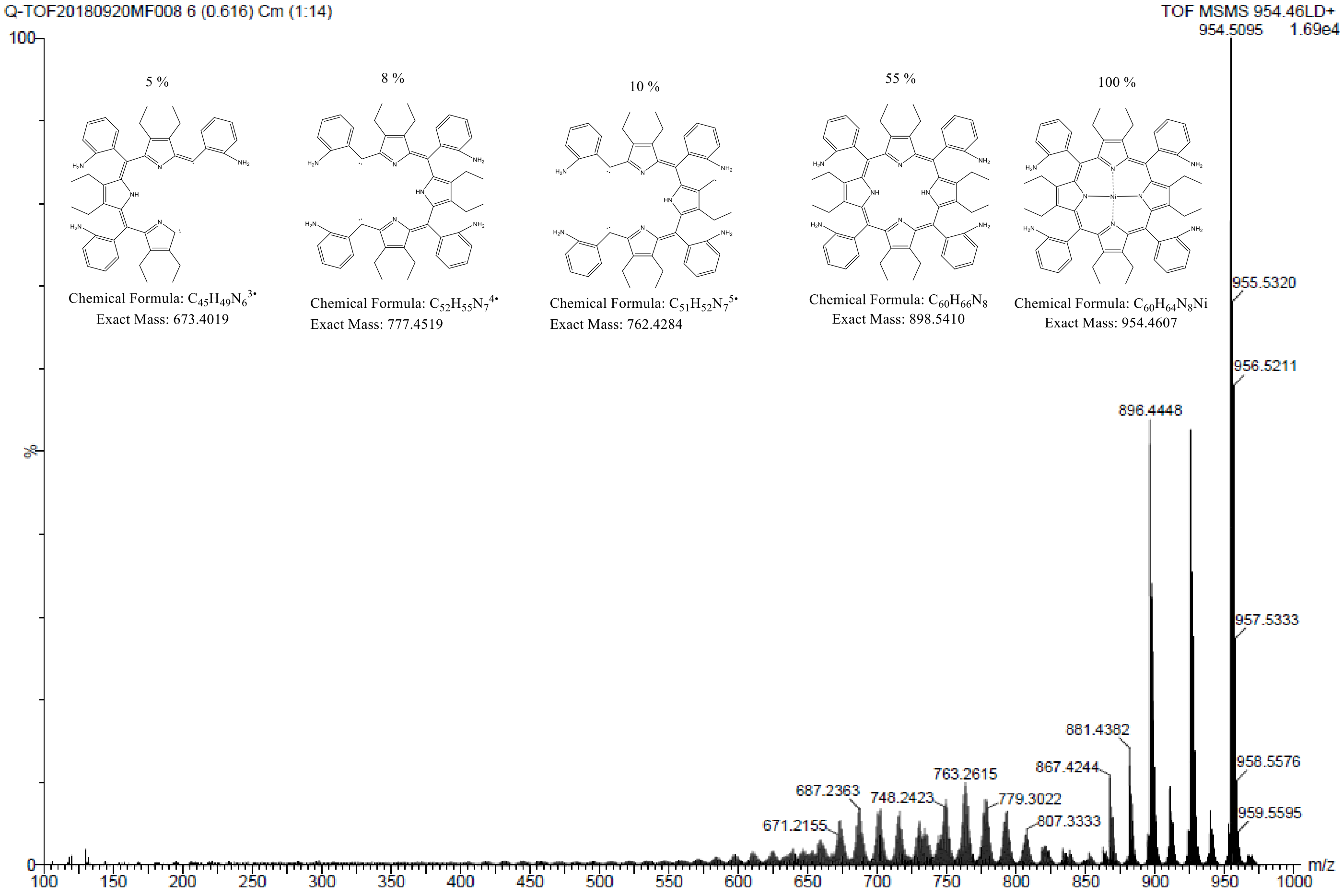


**Figure S26.** MSMS of *α*2,*β*2-**2**.

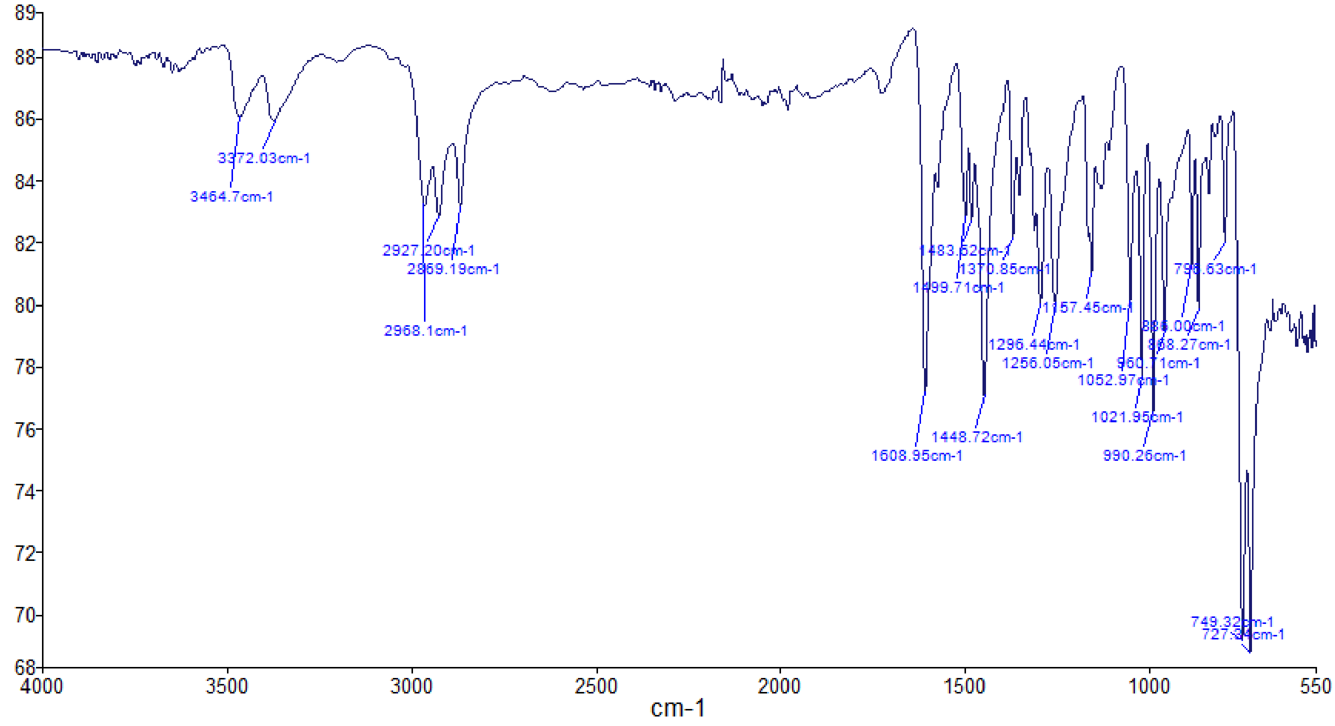


**Figure S27.** FTIR spectum of *α*2,*β*2-**2**.

# SUPPORTING INFORMATION

*Synthesis and characterization of $\alpha_3,\beta$-2.*

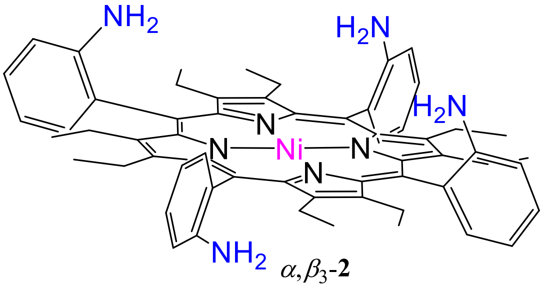


$\alpha,\beta_3$-**2**

In a 500 mL round bottom flask equipped with water condenser the atropisomeric mixture of 5,10,15,20-tetrakis(2-aminophenyl)-2,3,7,8,12,13,17,18-octaethylporphyrin (**1**, 685 mg; 0.762 mmol; 1 eq.), was dissolved in 120 mL of toluene at room temperature, followed by addition of excess nickel(II) acetylacetonate (0.891 g; 3.84 mmol; 5 eq.). The resulting brown mixture was quickly heated to 120 °C for 4 h. Upon removal of the solvent under reduced pressure, the purple solid was dissolved in dichloromethane and transferred to silica gel for column chromatography ($SiO_2$, dichloromethane:ethyl acetate = 100:1, v/v). Third brown/red band was collected and recrystallized using slow diffusion (chloroform:methanol) giving corresponding product $\alpha_3,\beta$-**2** ([α,β,β,β-5,10,15,20-Tetrakis(2-aminophenyl)-2,3,7,8,12,13,17,18-octaethylporphyrinato]nickel(II)) as purple platy habit crystals [332.0 mg; 0.347 mmol; 46 %]. M.p.: >300 °C; $R_f$ = 0.76 ($SiO_2$, dichloromethane); $^{1}$H NMR (400 MHz, *d*-chloroform, 25 °C): δ = 7.83 (d, *J* = 6.4 Hz, 1H, Ar–*H*), 7.68 (d, *J* = 7.1 Hz, 2H, Ar–*H*), 7.61 (d, *J* = 6.3 Hz, 1H, Ar–*H*), 7.45 (t, *J* = 7.5 Hz, 4H, Ar–*H*), 7.07 – 6.95 (m, 4H, Ar–*H*), 6.95 – 6.88 (m, 4H, Ar–*H*), 3.88 (s, 2H, N–*H*), 3.80 (s, 4H, N–*H*), 3.63 (s, 2H N–*H*), 2.64 – 2.27 (m, 16H,-C$H_2$), 0.71 – 0.54 (m, 24H, -C$H_3$). $^{13}$C NMR (101 MHz, *d*-chloroform, 25 °C): *δ* = 147.40, 147.34, 147.31, 145.62, 145.54, 145.53, 144.80, 144.74, 135.39, 135.34, 135.28, 124.89, 124.83, 118.17, 118.13, 118.02, 115.03, 114.88, 114.75, 112.38, 112.34, 112.30, 19.63, 16.73 ppm; UV/Vis (chloroform): $\lambda_{max}$ (log *ε*) = 439 (5.15), 559 (4.00), 596 nm (4.05); MS (MALDI) *m/z* (%) 955 (100) [$M^+$], 899 (50) [$M^+$ – Ni], 777 (8) [$M^+$ – $NiC_8H_{13}N$], 762 (10) [$M^+$ – $NiC_9H_{14}N$], 673 (5) [$M^+$ – $NiC_{15}H_{17}N_2$]; HRMS (MALDI) *m/z* calc. for $C_{60}H_{67}N_8$ [M]$^+$: 954.4607, found 954.4614; IR (ATR): $\tilde{\nu}$ = *3471.4, 3373.9, 2966.0, 2926.9, 2868.3, 1725.6, 1609.4, 1449.1, 1296.5, 1258.4, 1157.0 1052.7, 1021.5, 990.0, 749.0, 727.2* $cm^{-1}$.

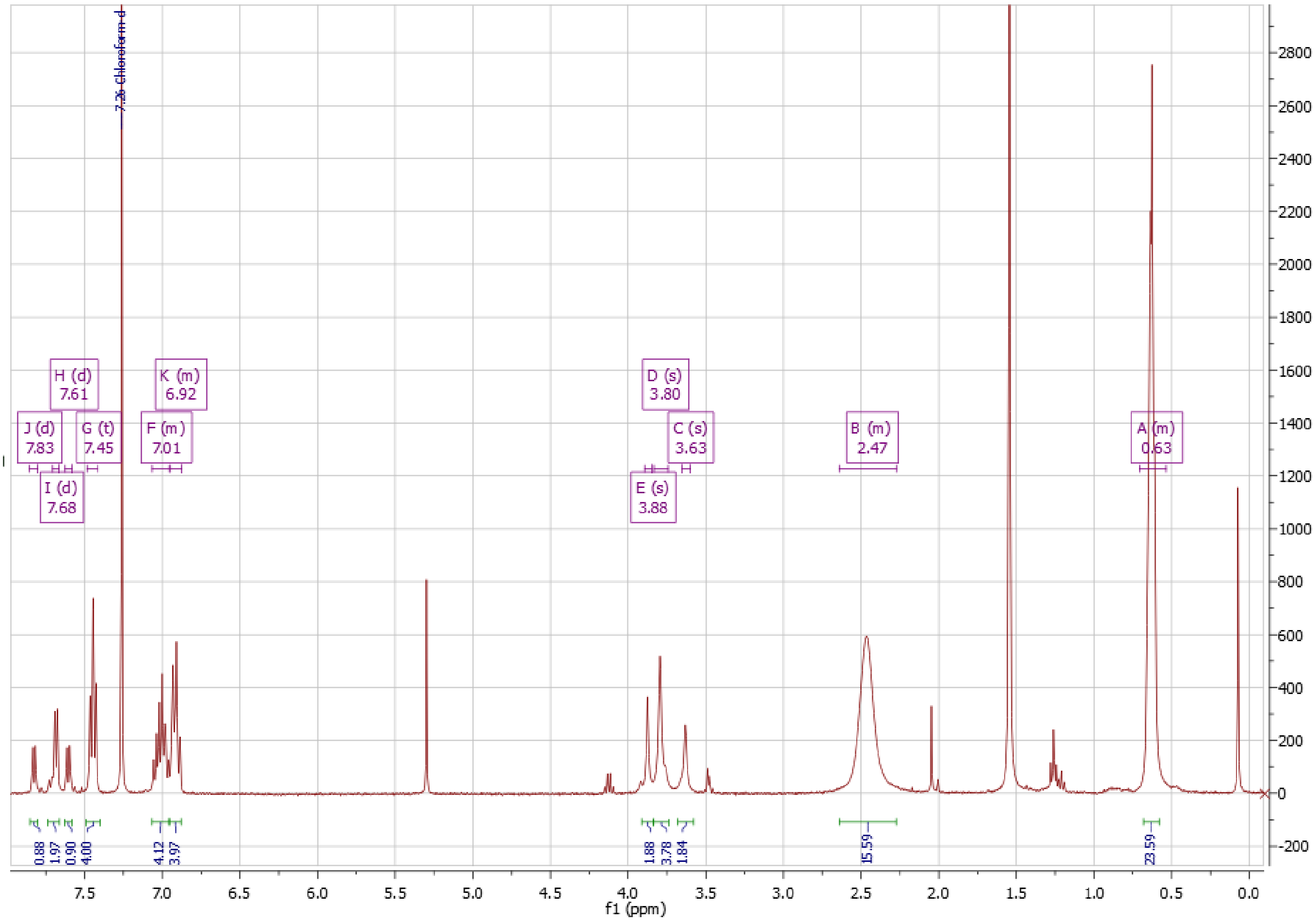


**Figure S28.** $^{1}$H NMR spectrum of $\alpha,\beta_3$-**2** (400 MHz, *d*-chloroform, 25 °C).

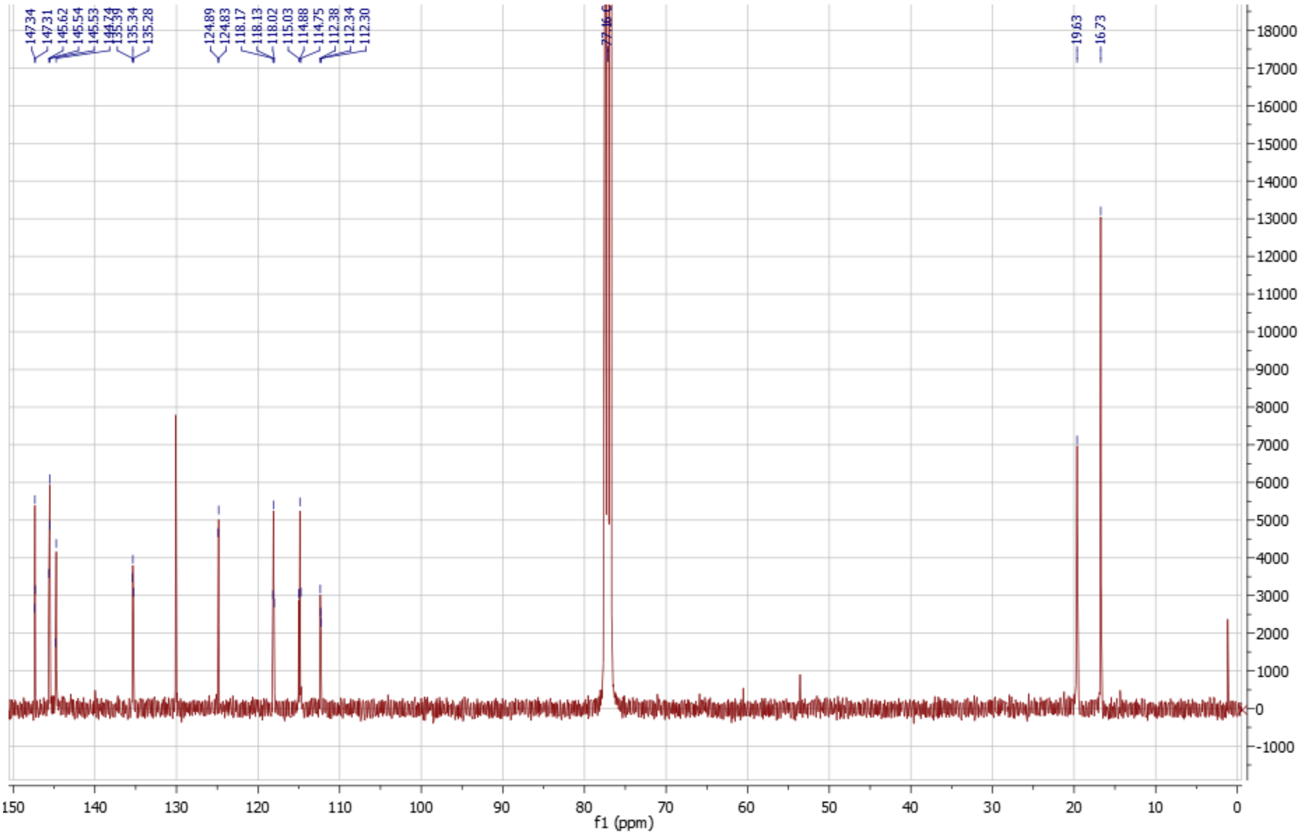


**Figure S29.** $^{13}$C NMR spectrum of *α*,$β_3$-**2** (101 MHz, *d*-chloroform, 25 °C).

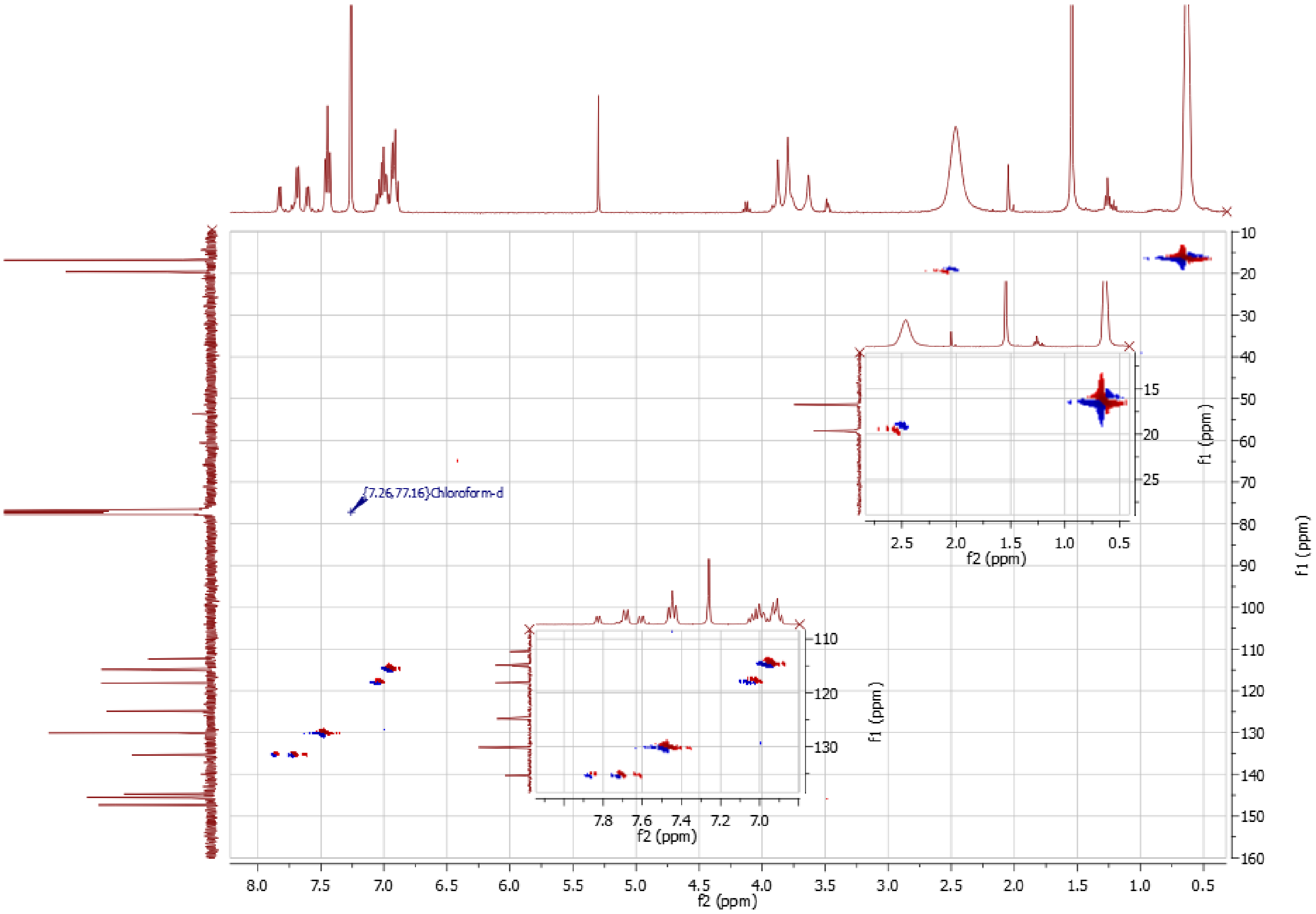


**Figure S30.** $^{1}$H-$^{13}$C HSQC of *α*,$β_3$-**2** (400 MHz, *d*-chloroform, 25 °C).

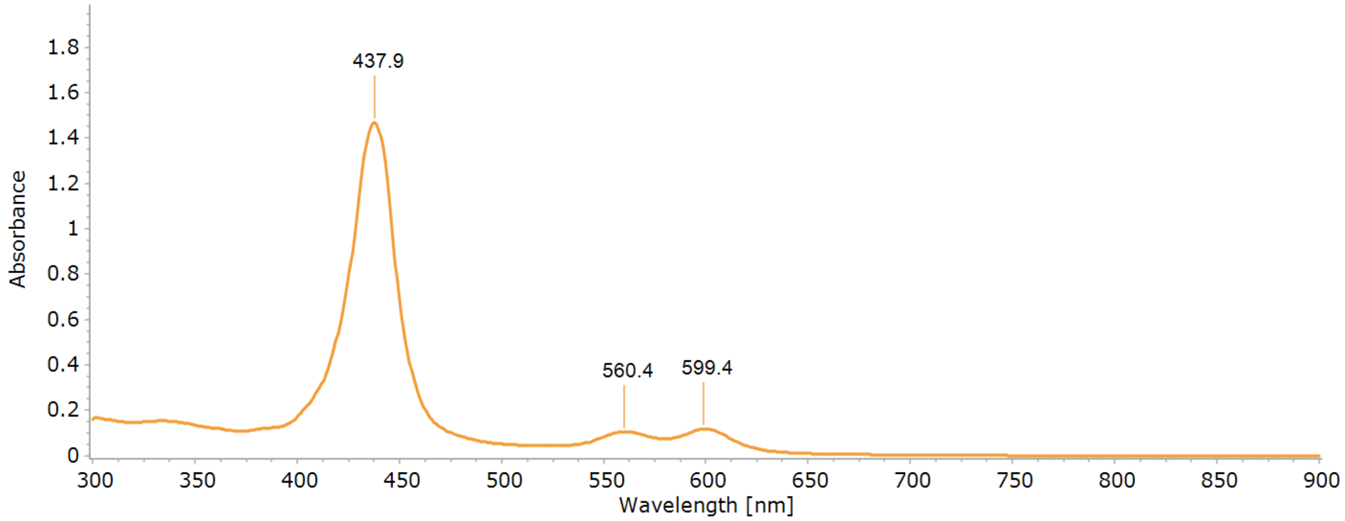


**Figure S31.** UV-vis spectrum of *α*,$\beta_3$-**2** in chloroform.

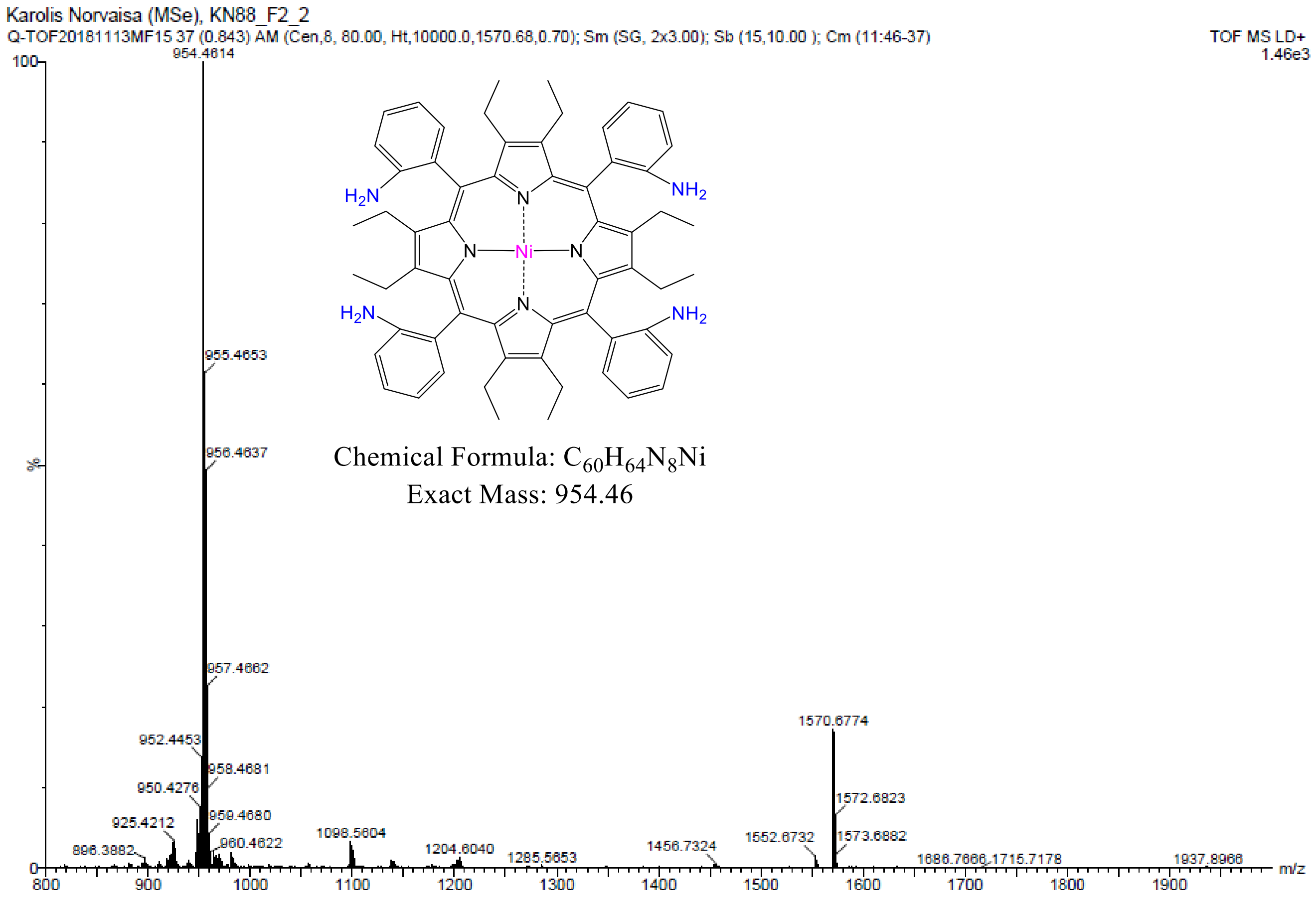


**Figure S32.** HRMS of *α*,$\beta_3$-**2**.

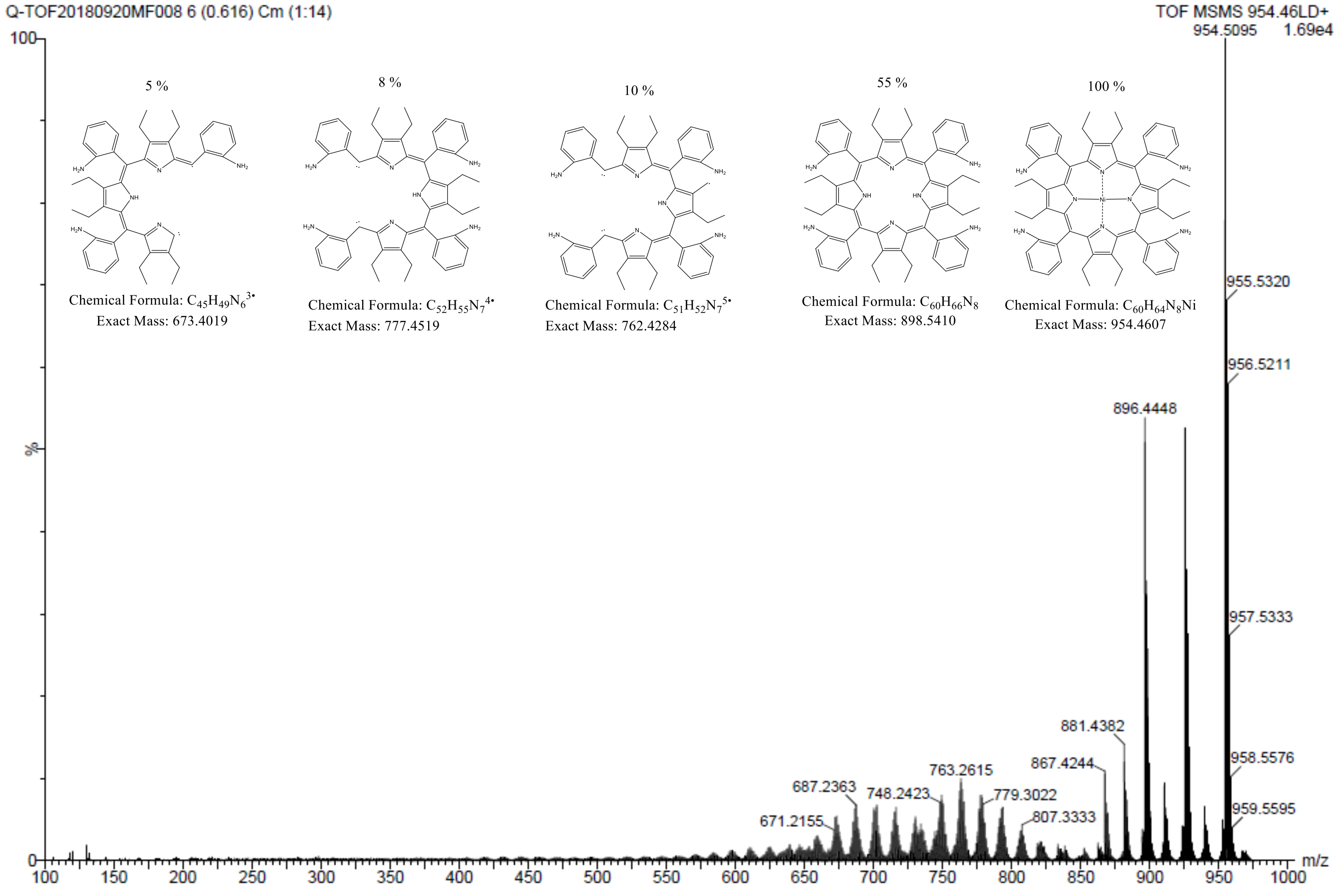


**Figure S32.** MSMS of $\alpha,\beta_3$-**2**.

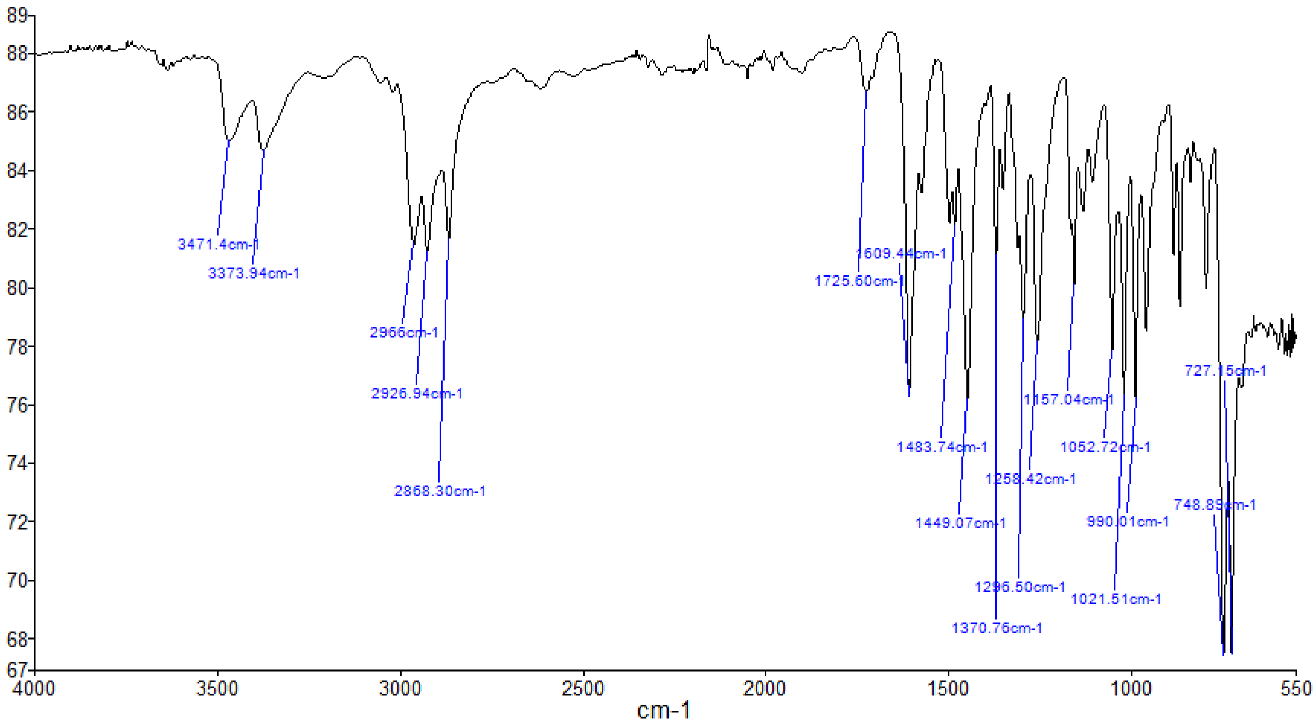


**Figure S33.** FTIR spectrum of $\alpha,\beta_3$-**2**.

# SUPPORTING INFORMATION

*Synthesis and characterization of α4-2*

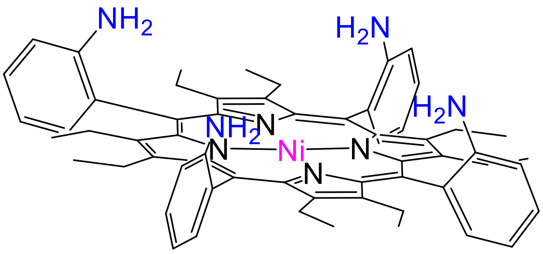


*α4*-**2**

In a 500 mL round bottom flask equipped with water condenser the atropisomeric mixture of 5,10,15,20-tetrakis(2-aminophenyl)-2,3,7,8,12,13,17,18-octaethyl-porphyrin (**1**, 685 mg; 0.762 mmol; 1 eq.), was dissolved in 120 mL of toluene at room temperature, followed by addition of excess nickel(II) acetylacetonate (0.891 g; 3.84 mmol; 5 eq.). The resulting brown mixture was quickly heated to 120 °C for 4 h. Upon removal of the solvent under reduced pressure, the purple solid was dissolved in dichloromethane and transferred to silica gel for column chromatography ($SiO_2$, dichloromethane:ethyl acetate = 3:1, v/v). Fourth brown/red band was collected and recrystallized using slow diffusion (chloroform:methanol) giving corresponding product *α4*-**2** ([α,α,α,α-5,10,15,20-Tetrakis(2-aminophenyl)-2,3,7,8,12,13,17,18-octaethylporphyrinato]nickel(II)) as purple platy habit crystals [102.0 mg; 0.107 mmol; 14 %]. M.p.: >300 °C; $R_f$ = 0.40 ($SiO_2$, dichloromethane); $^1$H NMR (400 MHz, d-chloroform, 25 °C): δ = 7.79 (d, *J* = 7.5 Hz, 4H, Ar–*H*), 7.45 (t, *J* = 7.7 Hz, 4H, Ar–*H*), 7.03 (t, *J* = 7.4 Hz, 4H, Ar–*H*), 6.90 (d, *J* = 8.0 Hz, 4H, Ar–*H*), 3.66 (s, 8H, N–*H*), 2.46 (s, 16H, -C*H2*), 0.63 (t, *J* = 7.4 Hz, 24H, -C*H3*); $^{13}$C NMR (101 MHz, d-chloroform, 25 °C): *δ* = 147.38, 145.61, 135.25, 130.08, 124.91, 118.21, 115.03, 112.29, 19.65, 16.70 ppm; UV/Vis (chloroform): $\lambda_{max}$ (log *ε*) = 437 (5.27), 559 (4.15), 596 nm (4.19); MS (MALDI) *m/z* (%) 955 (100) [M$^+$], 899 (50) [M$^+$ – Ni], 777 (8) [M$^+$ – $NiC_8H_{13}N$], 762 (10) [M$^+$ – $NiC_9H_{14}N$], 673 (5) [M$^+$ – $NiC_{15}H_{17}N_2$]; HRMS (MALDI) *m/z* calc. for $C_{60}H_{67}N_8$ [M]$^+$: 954.4607, found 954.4545; IR (ATR): $\tilde{\nu}$ = *3467.0, 3366.4, 2968.1, 2927.12, 2868.63, 1609.72, 1448.75, 1295.3, 1256.3, 1021.6, 990.8, 960.5, 747.7, 727.2* cm$^{-1}$

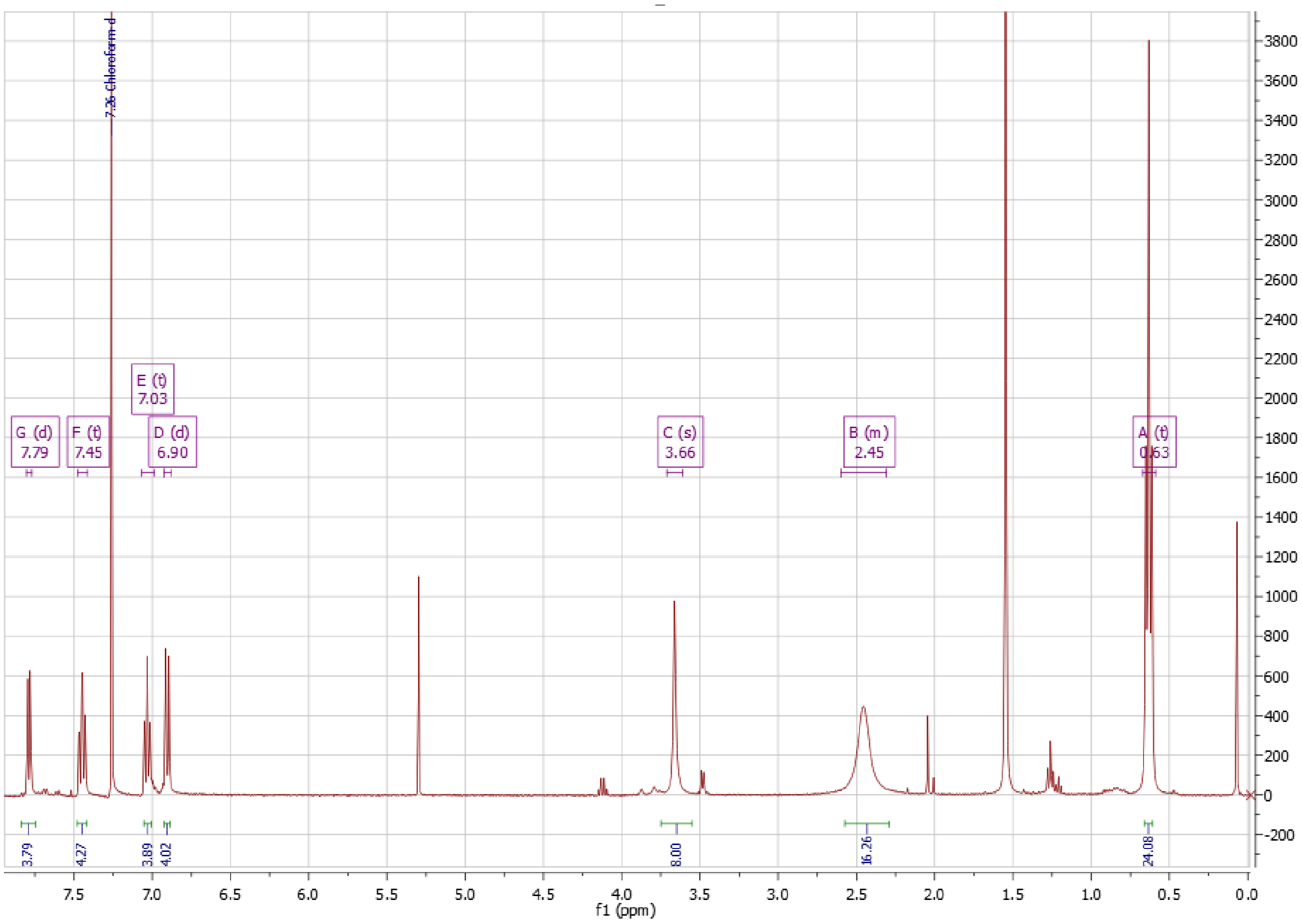


**Figure S34.** $^1$H NMR spectrum of *α4*-**2** (400 MHz, *d*-chloroform, 25 °C).

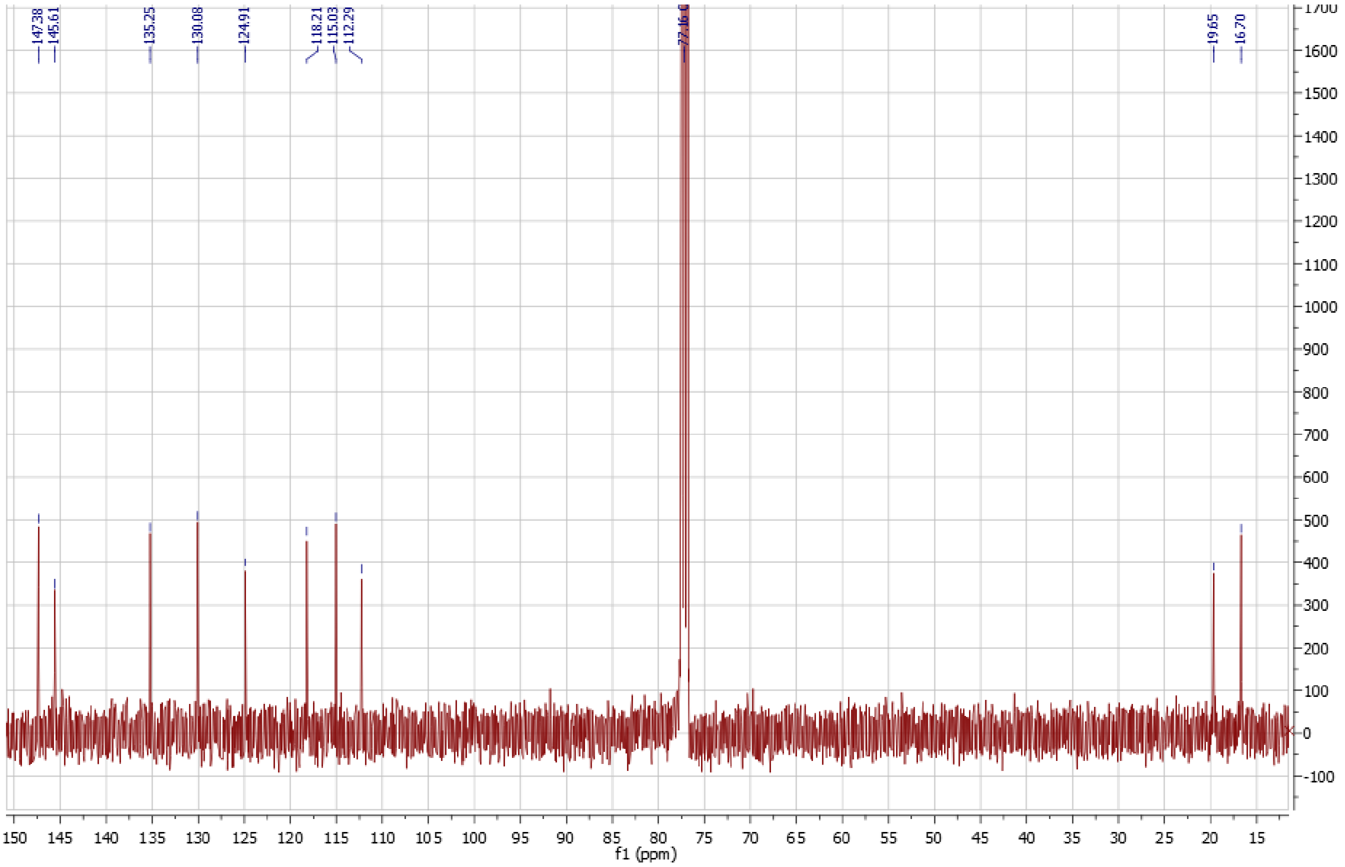


**Figure S35.** $^{13}C$ NMR spectrum of *α₄*-**2** (101 MHz, d-chloroform, 25 °C).

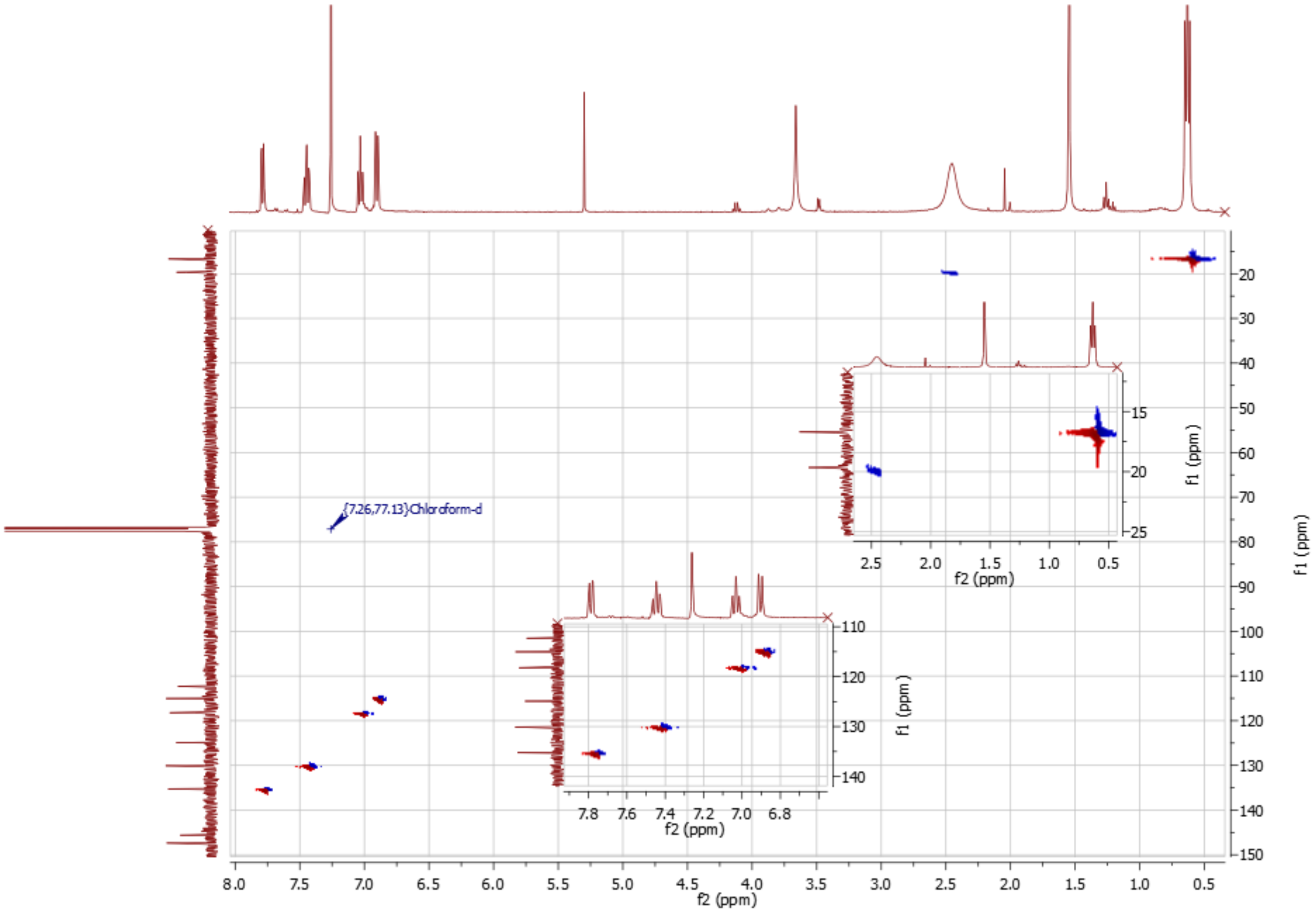


**Figure S36.** $^{1}H$-$^{13}C$ HSQC of *α₄*-**2** (400 MHz, *d*-chloroform, 25 °C).

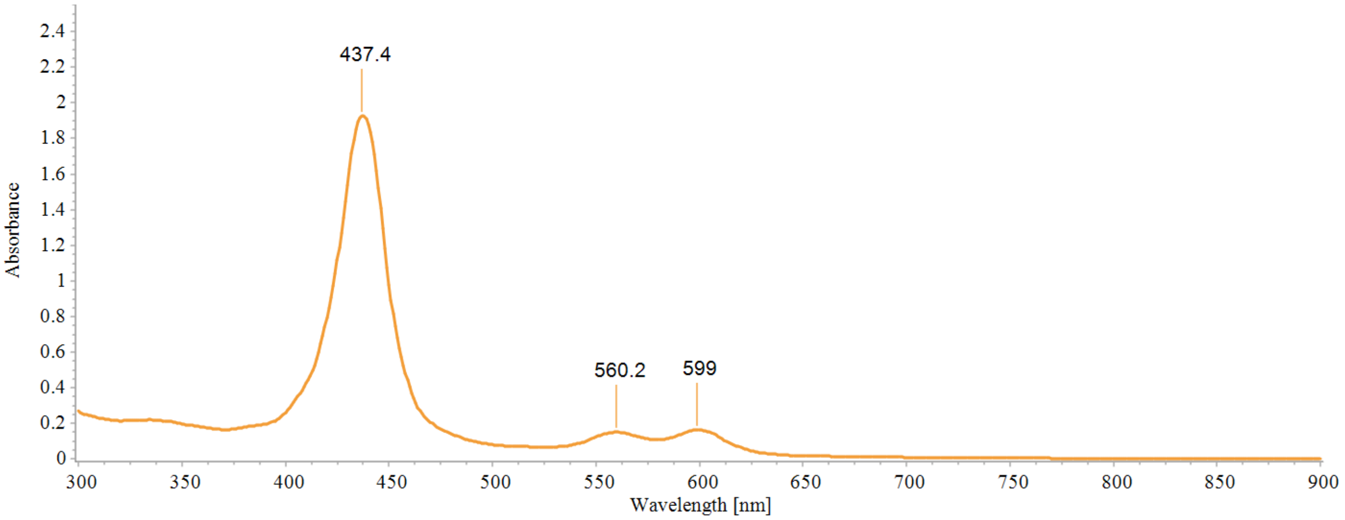


**Figure S37.** UV-vis spectrum of *α*₄-**2** in chloroform.

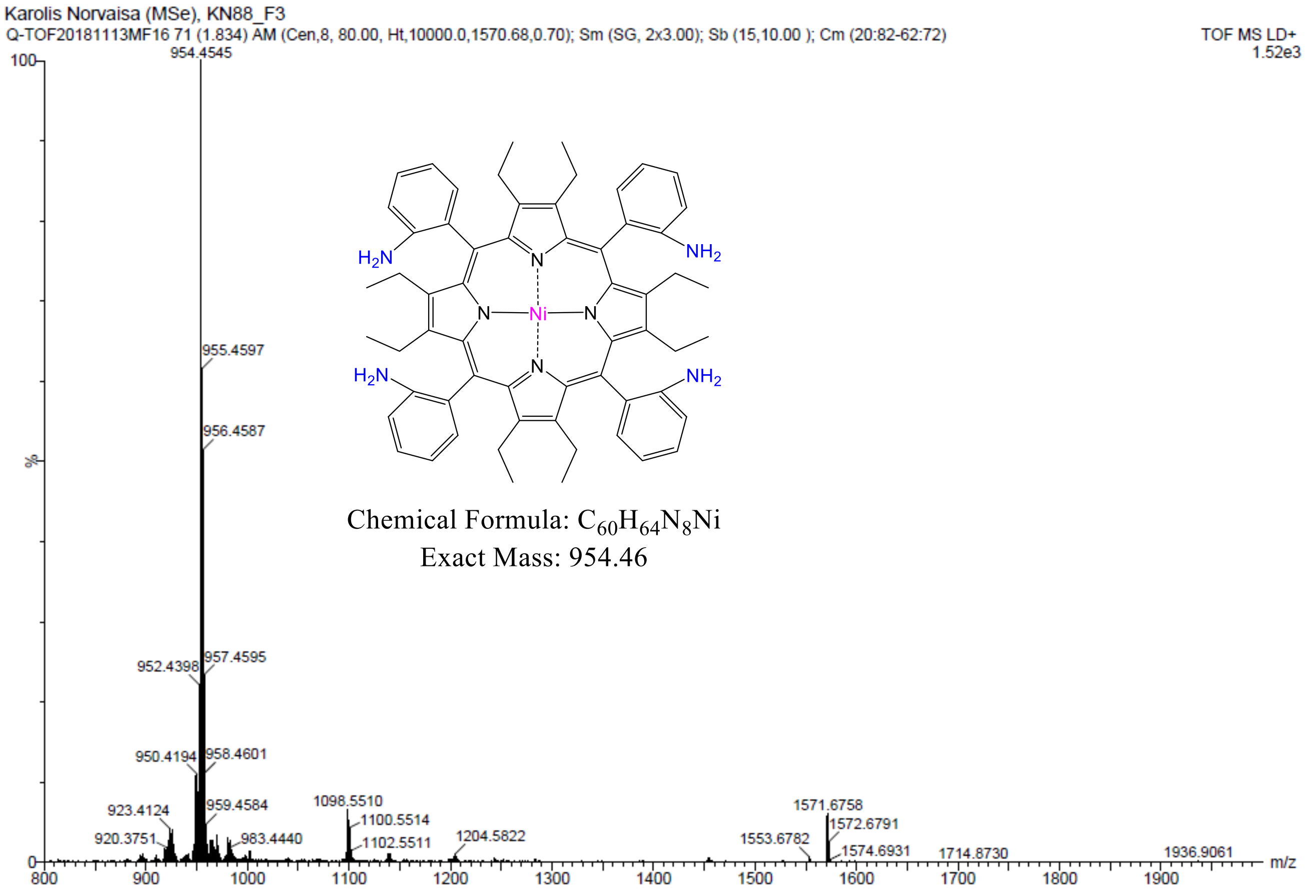


**Figure S38.** HRMS of *α*₄-**2**.

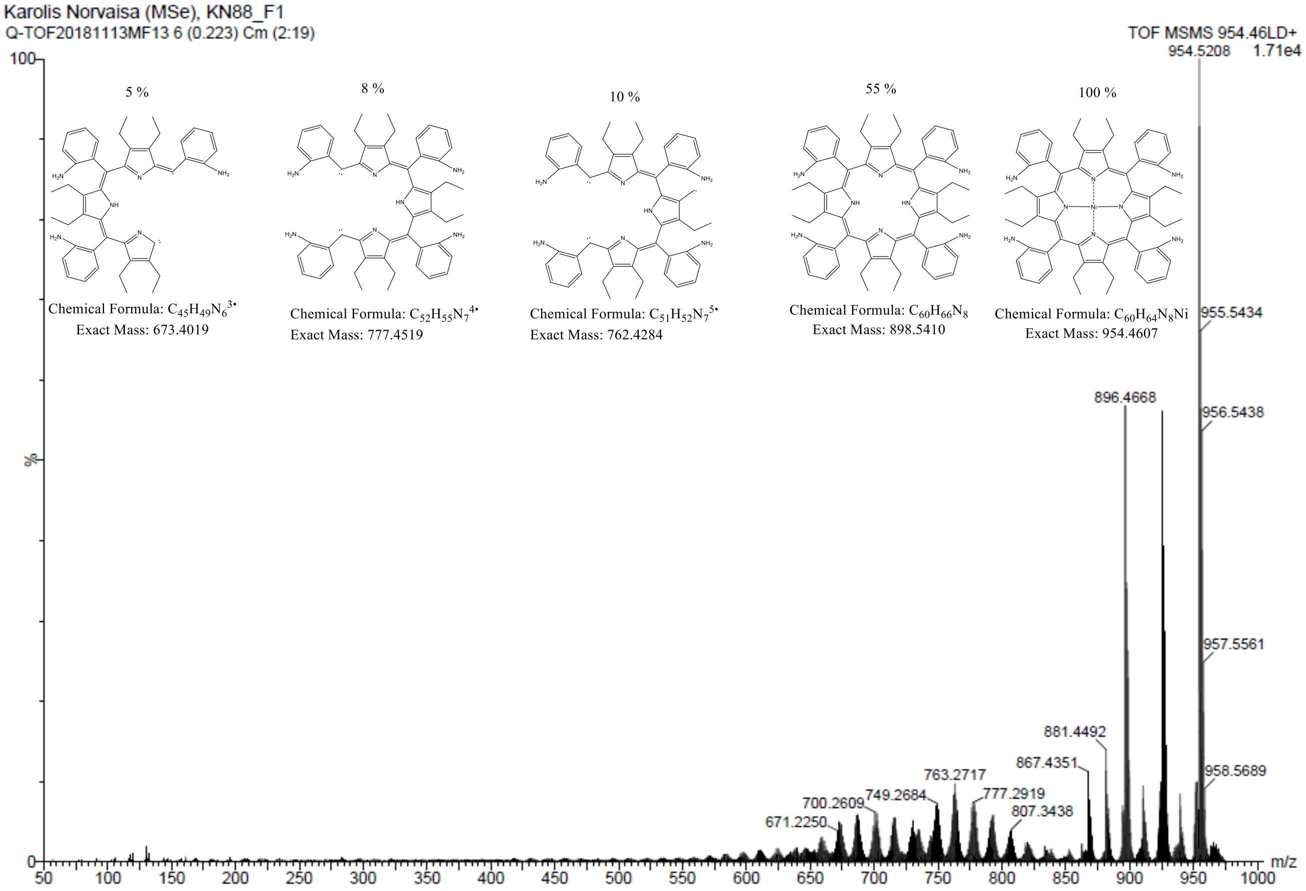


**Figure S39.** MSMS of $\alpha_4$-**2**.

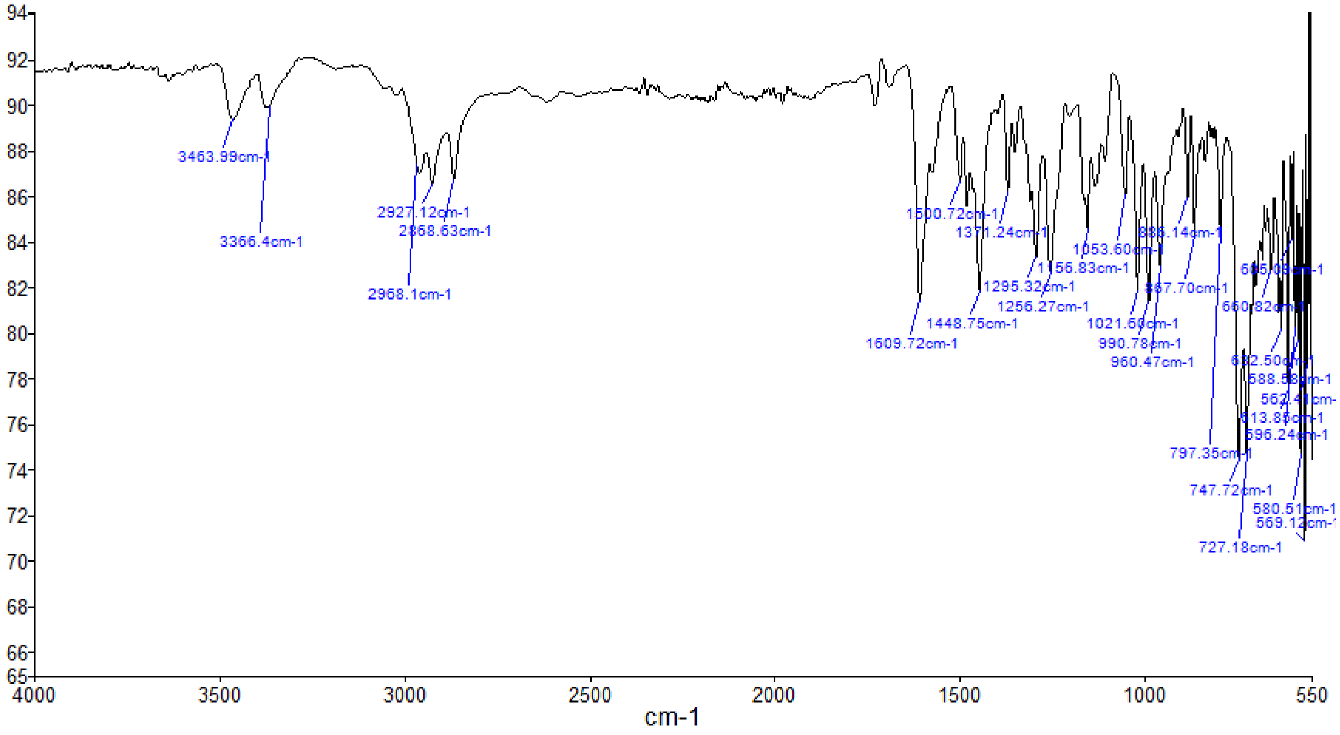


**Figure S40.** FTIR spectrum of $\alpha_4$-**2**.

## Results and Discussion

### Structural Determination of Receptor-Substrate Complexes and Atropisomers

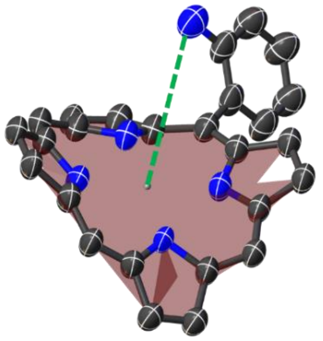

**Figure S41.** Distance (green dash line) from amine group to the least-squares-plane (maroon color surface).

The crystal structure of $\alpha_4$-**1** was obtained by X-ray crystallography after recrystallization of $\alpha_4$-**1** *via* slow diffusion technique (chloroform/methanol). Amine groups were found to be facing one side of the plane, thus, confirming the conformation of the most polar fraction isolated from the column chromatography as the ↑↑↑↑ atropisomer. The phenyl rings on the meso-positions are rotated at a 45° degree angle and the distance between the amine groups and the least-squares 24-atom plane of the porphyrin (Figure S41) is ~1.6 Å. The close proximity of amine groups to the macrocycle lead to a more compact fitting of the residues in comparison to one of the planar analogs of $\alpha_4$-**1**, *e.g.*, *α*,*β*,*α*,*β*-5,10,15,20-tetrakis(2-aminophenyl)-porphyrin.[5] The respective crystal structure, obtained from the Cambridge structural database,[6] shows phenyl ring rotation angles of ~70° and the distance between the amine groups and the least-squares-plane of the planar porphyrin was found to be 2.4 Å. As the amine groups offer multiple hydrogen-bond donating sites, further analysis was carried out using sulfuric acid as a hydrogen bond acceptor. The porphyrin $\alpha_4$-**P1**•$[SO_4^{2-}][HSO_4^{-}]_4$ was recrystallized from the acidic solution (presence of sulfuric acid) using liquid-liquid diffusion in dichloromethane and methanol. The crystalline compound was analyzed by X-ray crystallography (Figure S42). There is a high occurrence of hydrogen-bonding between the sulfate anions and $\alpha_4$-**1** ammonium. The inner core system was shown to be capable of accommodating two sulfuric acid entities, showing a metal-free porphyrin complexation pattern. This indicates that inner core interactions occur only with the corresponding counter anions in the current system. All of the isolated Ni(II)OET$_{am}$PP (**2**) atropisomers were recrystallized using liquid-liquid diffusion in chloroform and methanol. The samples analyzed by X-ray crystallography confirmed the corresponding conformations (Figure S41).

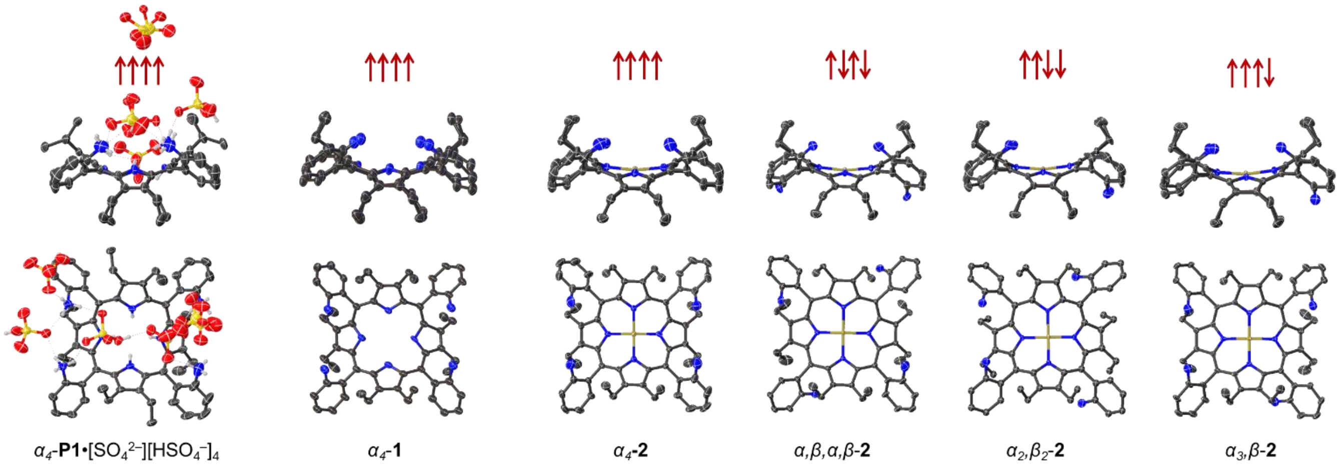


**Figure S42.** X-ray structures (single units) of $\alpha_4$-**P1**•$[SO_4^{2-}][HSO_4^{-}]_4$; $\alpha_4$-**1** and different Ni(II)OET$_{am}$PP (**2**) atropisomers (non-essential hydrogens omitted for clarity and thermal ellipsoids give 50% probability).

In the crystal packing, multiple hydrogen-bonds were observed in a $\alpha_4$-**P1**•$[SO_4^{2-}][HSO_4^{-}]_4$ crystal structure, showing intermolecular interactions of porphyrins and small solvent molecules (Figure S44). The corresponding stacking pattern exhibit solvent specific rearrangements, rising the distances between the two closest porphyrin 24-atom planes (Figure S43) from 7.748 Å ($\alpha_4$-**1**) to 12.384 Å ($\alpha_4$-**P1**•$[SO_4^{2-}][HSO_4^{-}]_4$). All of the isolated Ni(II)OET$_{am}$PP (**2**) atropisomer units and free base $\alpha_4$-**1** form arrays of parallel channels (Figure S44). The solvent accessible voids were found to be 242.4 Å$^3$ for the free base $\alpha_4$-**1** porphyrin and $\alpha_3$,*β*-**2** (321.6 Å) < $\alpha_2$,$\beta_2$-**2** (333.3 Å)< $\alpha_4$-**2** (340.2 Å) < *α*,*β*,*α*,*β*-**2** (343.7 Å) for the Ni(II) atropisomers. These molecular pores unlock the potential for application in the design of molecular porphyrin sponges.[7] The arrays of channels could serve as microporous material for absorption of small molecules.

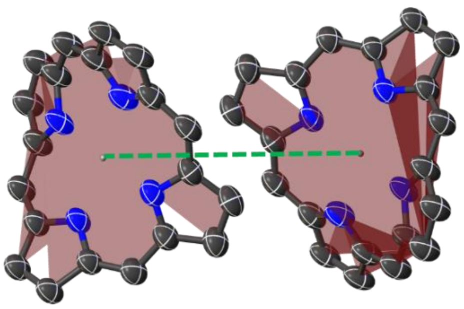

**Figure S43.** Distance (green dash line) between two least-squares-planes of porphyrins (maroon color surfaces).

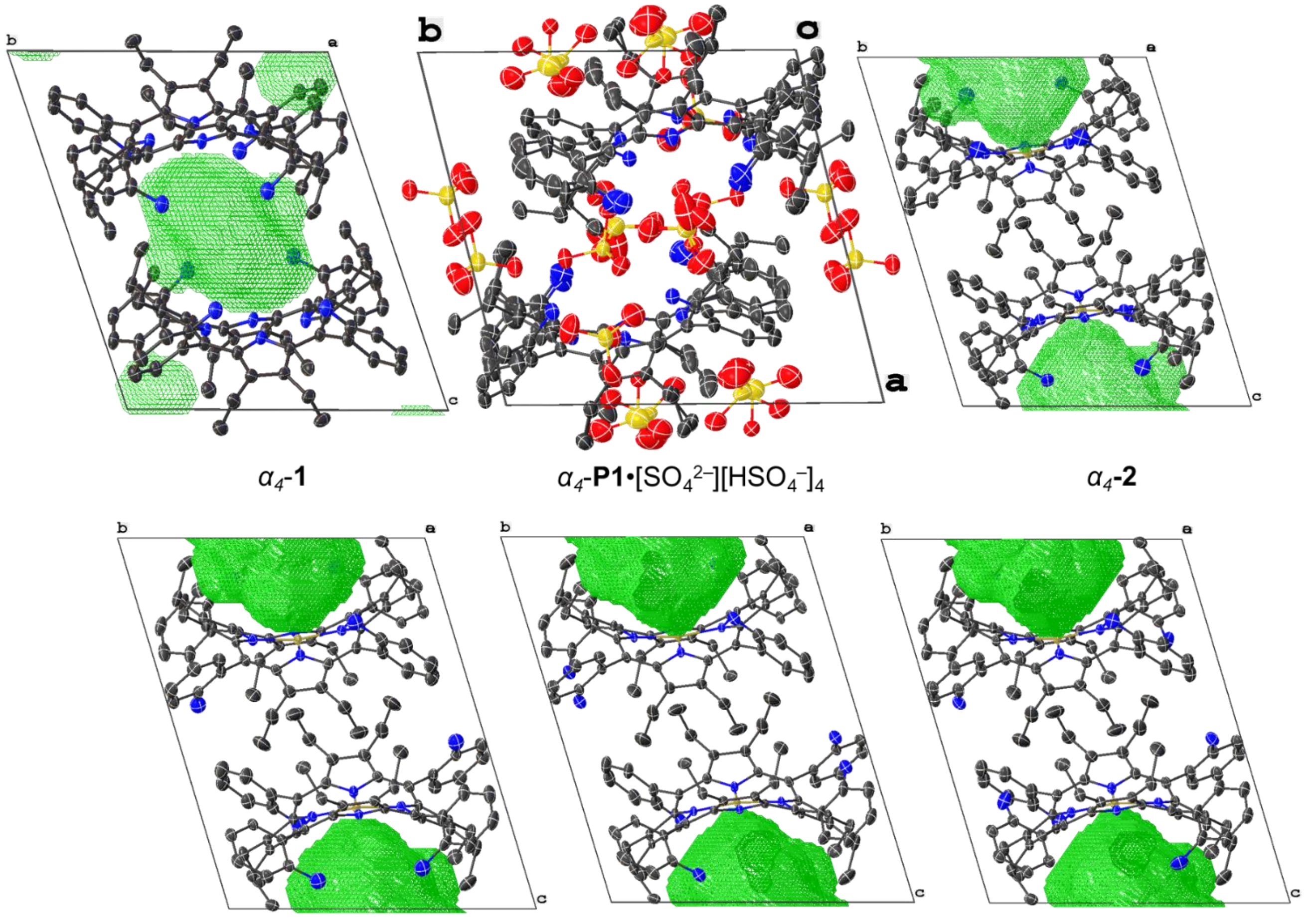

$\alpha_4$-**1** $\alpha_4$-**P1**•[$SO_4^{2-}$][$HSO_4^-$]$_4$ $\alpha_4$-**2**

$\alpha_3,\beta$-**2** $\alpha_2,\beta_2$-**2** $\alpha,\beta,\alpha,\beta$-**2**

**Figure S44.** View of the molecular structure of $\alpha_4$-**P1**•[$SO_4^{2-}$][$HSO_4^-$]$_4$; $\alpha_4$-**1** and different Ni(II)OET$_{am}$PP (**2**) atropisomers (with voids highlighted in green) in crystal packing systems (non-essential hydrogens omitted for clarity and thermal ellipsoids give 50% probability).

To compare the conformational distortion of isolated crystal structures, the normal-coordinate structural decomposition (NSD) analysis for out-of-plane (*oop*) and in-plane (*ip*) distortions was performed (Figure S45). Overall, the samples displayed high levels of *sad*dle distortion with minimal difference in total out-of-plane distortions (*Doop*). The lowest 24-atom plane alterations were observed for $\alpha_4$-**1**, while the highest value of *Doop* was detected in $\alpha_4$-**P1**•[$SO_4^{2-}$][$HSO_4^-$]$_4$ indicating a correlation of the distortion level with the substrate interactions. Regarding in-plane distortion of the free base porphyrins, strong *bre*ath deformations with a small difference ($\alpha_4$-**1** < $\alpha_4$-**P1**•[$SO_4^{2-}$][$HSO_4^-$]$_4$) in total in-plane distortions (*Dip*) were observed. In terms of comparing *Doop* in free base $\alpha_4$-**1** and the metalated (Ni(II)) analog ($\alpha_4$-**2**), almost no difference in distortion was detected. Thus, concluding that metalation in the corresponding highly substituted porphyrin core system does contribute significantly to the level of *oop* distortion. However, the in-plane *bre*ath deformation has increased by ~0.3 Å signifying that the porphyrin macrocycle contracts after nickel(II) insertion. In addition, the NSD analysis for out-of-plane and in-plane deformations was performed with the Ni(II)OET$_{am}$PP (**2**) atropisomers (Figure S45). All macrocycles displayed high levels of *sad*dle and *bre*ath distortions. Only minimal differences in overall *Doop* and *Dip* values were found for the individual atropisomers, thus, showing that conformation has little influence towards the overall distortion levels.

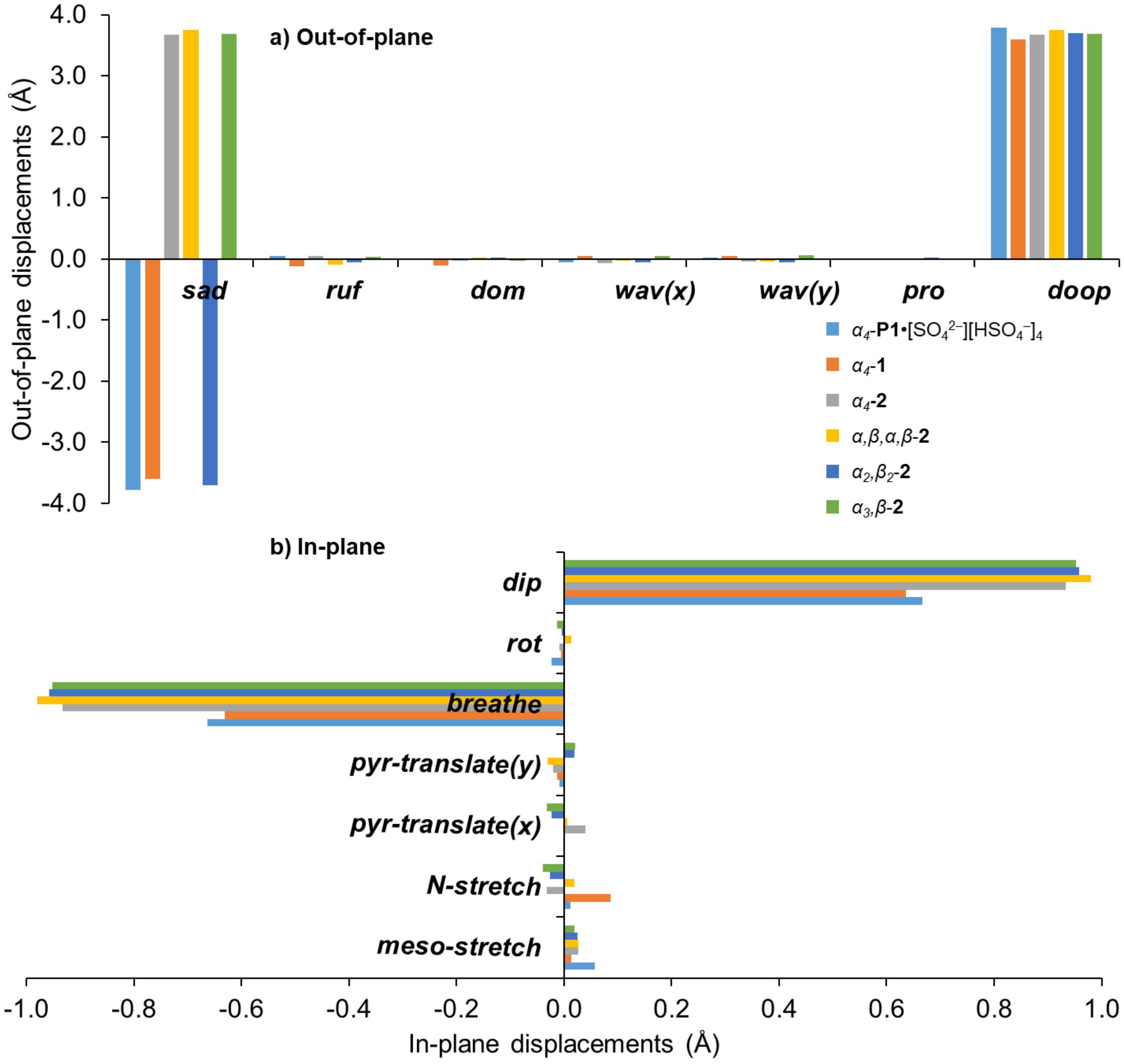


**Figure S45.** a) out-of-plane and b) in-plane normal-coordinate structural decomposition analysis of *α*$_4$-**P1**•[$SO_4^{2-}$][$HSO_4^-$]$_4$; *α*$_4$-**1** and different Ni(II)OET$_{am}$PP (**2**) atropisomers.

Crystals were grown following the protocol developed by Hope, liquid-liquid diffusion in $CHCl_3$ and methanol.[8] Using Olex2, the structure was solved with the XT structure solution program, using the intrinsic phasing solution method and refined against │$F^2$│ with XL using least squares minimization.[9] The C and N bound H atoms were placed in their expected calculated positions and refined as riding model: N–H = 0.88 Å, C–H = 0.95–0.98 Å, with $U_{iso}$ (H) = 1.5$U_{eq}$ (C) for methyl H atoms and 1.2$U_{eq}$ (C, N) for all other atoms other H atoms. Details of data refinements can be found in Table S1. All images were prepared by using Olex2.[9a]

In the structure of [*α₄*-**1**]$^{+6}$•[$SO_4^{2-}$][$HSO_4^-$]$_4$, one of the solvent $HSO_4^-$ molecule was modeled over two positions (S4, S2A) using rigid models and SIMU restraint in a 70:30 % occupancy. Moreover, one of the solvent $HSO_4^-$ molecule was modeled over three positions (S1, S1A, S1B) using rigid models and restraint SIMU in a 30:30:40 % occupancy. Two of the phenyl rings at C5 and C10 were modeled over two positions in a 70:30 % occupancy and fixed using command AFIX 66 and SIMU and SADI. In the structure there were solvent accessible voids that contained large amount of solvent molecules, however, due to high disorder these could not be modelled and were omitted using PLATON squeeze.

In the structure of $\alpha_4$-**1** the phenyl groups at C5, C15, and C20 were modelled over two positions using restraints (RIGU, SADI and AFIX 66) in a 75:25 %, 50:50 %, 60:40 % occupancies respectively. The ethyl groups at C2, C12, C13, C17, C18 were modeled over two positions using restraints (SADI, DFIX) in a 50:50 % occupancy. Two ethyl groups, pyrrole and phenyl rings between C5 and C11 were modelled over two positions using restraints (RIGU, SADI and AFIX 66) in a 50:50 % occupancy. In the structure there were solvent accessible voids that contained large amount of solvent molecules, however, due to high disorder these could not be modelled and were omitted using PLATON squeeze.

In the structure of $\alpha_4$-**2** the phenyl groups at C5, C10, C15, and C20 were modelled over two positions using restraints (AFIX 66, SADI, DFIX) in a 80:20 % occupancy. The ethyl groups at C2, C3 were modeled over two positions using restraints (SADI, SIMU) in a 50:50 % occupancy. In the structure there were solvent accessible voids that contained large amount of solvent molecules, however, due to high disorder these could not be modelled and were omitted using OLEX2 maps.

In the structure of *α*,*β*,*α*,*β*-**2** the phenyl moiety at C5 was modeled over two positions using restraints (SADI, SIMU) in a 60:40 % occupancy. Additionally, the phenyl moieties at C10, C15, and C20 were modelled over three positions using restraints (SADI, ISOR, SIMU, AFIX 66) in a 60:20:20 %, 60:30:10 %, 60:20:20 % occupancies respectively. The ethyl groups at C2, C3, C8, C12, C13, C17, C18 were modeled over two positions using restraints (SADI, SIMU) in a 60:40 %, 30:70 %, 50:50 %, 20:80 %, 20:80 %, 70:30 %, 70:30 % occupancies respectively. In the structure there were solvent accessible voids that contained large amount of solvent molecules, however, due to high disorder these could not be modelled and were omitted using OLEX2 maps.

In the structure of $\alpha_2$,$\beta_2$-**2** the phenyl moieties at C5 and C20 were modeled over two positions using restraints (SADI, SIMU, ISOR, AFIX 66) in a 60:40 % occupancy. Additionally, the phenyl moieties at C10 and C15 were modelled over three positions using restraints (SADI, ISOR, SIMU, AFIX 66) in a 60:30:10 % and 40:40:20 % occupancies respectively. The ethyl group at C2, was modeled over two positions using restraints (SADI, SIMU) in a 70:30 % occupancy. In the structure there were solvent accessible voids that contained large amount of solvent molecules, however, due to high disorder these could not be modelled and were omitted using PLATON squeeze.

In the structure of $\alpha_3$,*β*-**2** the phenyl moieties at C5, C10 and C15 were modeled over two positions using restraints (SADI, SIMU, ISOR, AFIX 66) in a 70:30 % occupancy. Additionally, the phenyl group at C20 was modelled over three positions using restraints (SADI, ISOR, SIMU, AFIX 66) in a 40:30:30 % occupancy respectively. The ethyl groups at C7, C8, C18 were modeled over two positions using restraints (SADI, SIMU) in a 30:70 %, 40:60 %, 50:50 % occupancies respectively. In the structure there were solvent accessible voids that contained large amount of solvent molecules, however, due to high disorder these could not be modelled and were omitted using PLATON squeeze.

**Table S1:** Details of XRD data refinement of *α*4-**1**, *α*4-**P1** and atropisomers of **2**

| ***Compound*** | *α*4-**1** | *α*4-**P1**•[$SO_4^{2-}$][$HSO_4^{-}$]$_4$ | *α*,*β*,*α*,*β*-**2** | *α*2,*β*2-**2** | *α*3,*β*-**2** | *α*4-**2** |
|---|---|---|---|---|---|---|
| *Empirical formula* | $C_{60}H_{66}N_8$ | $C_{60}H_{76}N_8O_{20}S_5$ | $C_{60}H_{64}N_8Ni$ | $C_{60}H_{64}N_8Ni$ | $C_{60}H_{64}N_8Ni$ | $C_{60}H_{64}N_8Ni$ |
| *Formula weight* | 899.20 | 1389.58 | 955.90 | 955.90 | 955.90 | 955.90 |
| *Temperature/K* | 100(2) | 100(2) | 100(2) | 100(2) | 100(2) | 100(2) |
| *Crystal system* | Triclinic | triclinic | triclinic | triclinic | triclinic | triclinic |
| *Space group* | $P\bar{1}$ | $P\bar{1}$ | $P\bar{1}$ | $P\bar{1}$ | $P\bar{1}$ | $P\bar{1}$ |
| *a/Å* | 13.7413(7) | 13.814(3) | 13.4248(5) | 13.3586(5) | 13.3804(10) | 13.4579(4) |
| *b/Å* | 13.7523(6) | 14.092(3) | 13.7473(5) | 13.7341(4) | 13.7211(9) | 13.6826(4) |
| *c/Å* | 15.6854(8) | 23.801(5) | 16.5290(6) | 16.5483(6) | 16.5334(12) | 16.5352(5) |
| *α/°* | 105.764(3) | 91.09(3) | 103.451(2) | 103.6760(10) | 103.534(2) | 103.419(2) |
| *β/°* | 97.115(4) | 100.61(3) | 96.666(2) | 96.8560(10) | 96.861(2) | 97.423(2) |
| *γ/°* | 106.228(3) | 103.15(3) | 108.5110(10) | 108.1340(10) | 108.216(2) | 108.245(2) |
| *Volume/Å$^3$* | 2674.2(2) | 4425.2(17) | 2753.02(18) | 2741.59(16) | 2741.6(3) | 2744.79(15) |
| *Z* | 2 | 2 | 2 | 2 | 2 | 2 |
| *$D_{calc}$ g/cm$^3$* | 1.117 | 1.043 | 1.153 | 1.158 | 1.158 | 1.157 |
| *μ/mm$^{-1}$* | 0.510 | 1.706 | 0.396 | 0.398 | 0.398 | 0.844 |
| *F(000)* | 964 | 1464.0 | 1016.0 | 1016.0 | 1016.0 | 1016.0 |
| *Crystal size/mm$^3$* | 0.233 x 0.033 x 0.019 | 0.024 × 0.023 × 0.003 | 0.5 × 0.3 × 0.2 | 0.35 × 0.35 × 0.3 | 0.15 × 0.08 × 0.08 | 0.2 × 0.17 × 0.05 |
| *Radiation* | $CuK_α$ | $CuK_α$ | $MoK_α$ | $MoK_α$ | $MoK_α$ | $CuK_α$ |
| *Wavelength/Å* | λ = 1.54178 | λ = 1.54178 | λ = 0.71073 | λ = 0.71073 | λ = 0.71073 | λ = 1.54178 |
| *2θ/°* | 2.998–67.094 | 6.70–136.69 | 3.27–61.20 | 3.26–61.31 | 3.276–52.83 | 5.63–133.51 |
| *Reflections collected* | 28255 | 88502 | 326701 | 112076 | 123704 | 54689 |
| *Independent reflections* | 9455 | 16030 | 16880 | 16874 | 11228 | 9656 |
| *$R_{int}$* | 0.0627 | 0.0369 | 0.0383 | 0.0351 | 0.0685 | 0.0671 |
| *$R_{sigma}$* | 0.0801 | 0.0242 | 0.0158 | 0.0251 | 0.0384 | 0.0440 |
| *Restraints* | 1906 | 651 | 1694 | 1096 | 1184 | 1312 |
| *Parameters* | 1008 | 1140 | 1119 | 953 | 872 | 832 |
| *GooF* | 1.043 | 1.032 | 1.119 | 1.032 | 1.027 | 1.072 |
| *$R_1$ [I> 2σ (I)]* | 0.0784 | 0.0674 | 0.0469 | 0.0415 | 0.0461 | 0.0580 |
| *$wR_2$ [I> 2σ (I)]* | 0.2275 | 0.1903 | 0.1182 | 0.1048 | 0.1088 | 0.1554 |
| *$R_1$ [all data]* | 0.1284 | 0.0759 | 0.0554 | 0.0553 | 0.0701 | 0.0704 |
| *$wR_2$ [all data]* | 0.2712 | 0.2003 | 0.1229 | 0.1122 | 0.1197 | 0.1653 |
| *Largest peak/e Å$^{-3}$* | 0.32 | 1.09 | 0.74 | 0.69 | 0.77 | 0.60 |
| *Deepest hole/e Å$^{-3}$* | -0.30 | -0.48 | -0.48 | -0.47 | -0.63 | -0.58 |

### UV-Vis Spectrophotometry Titration Studies

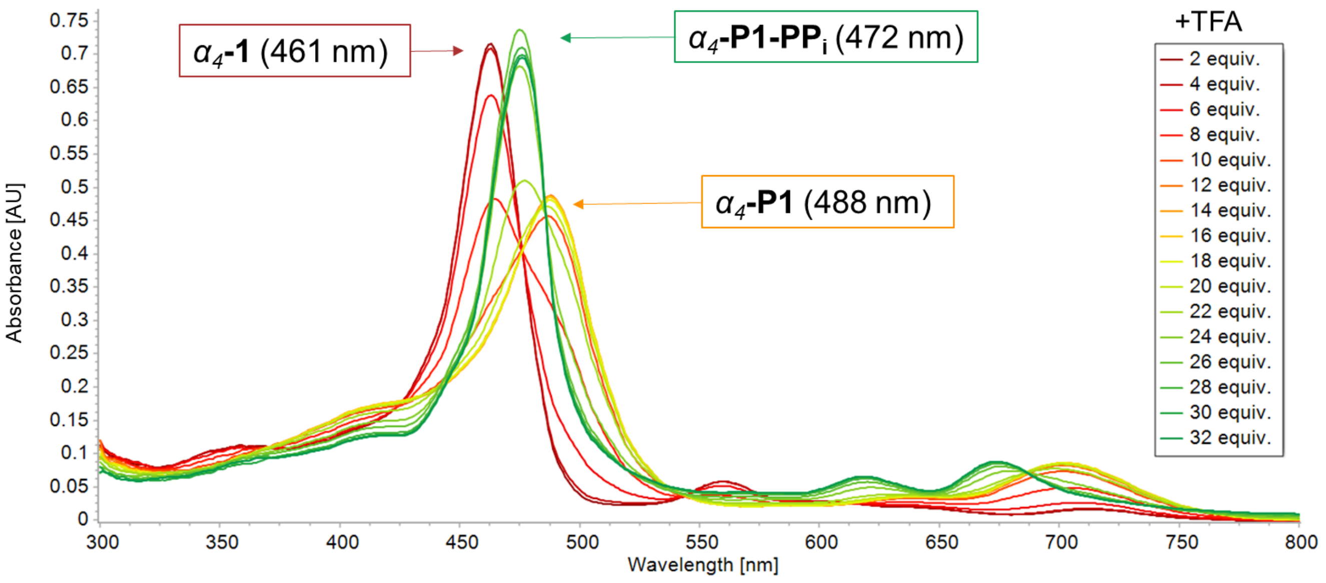


**Figure S46.** UV-vis titration of neutral porphyrin *α4*-**P1** (5.56 × $10^{-6}$ M) with TFA in the presence of 12 eq. of pyrophosphate salt **PP**$_i$.

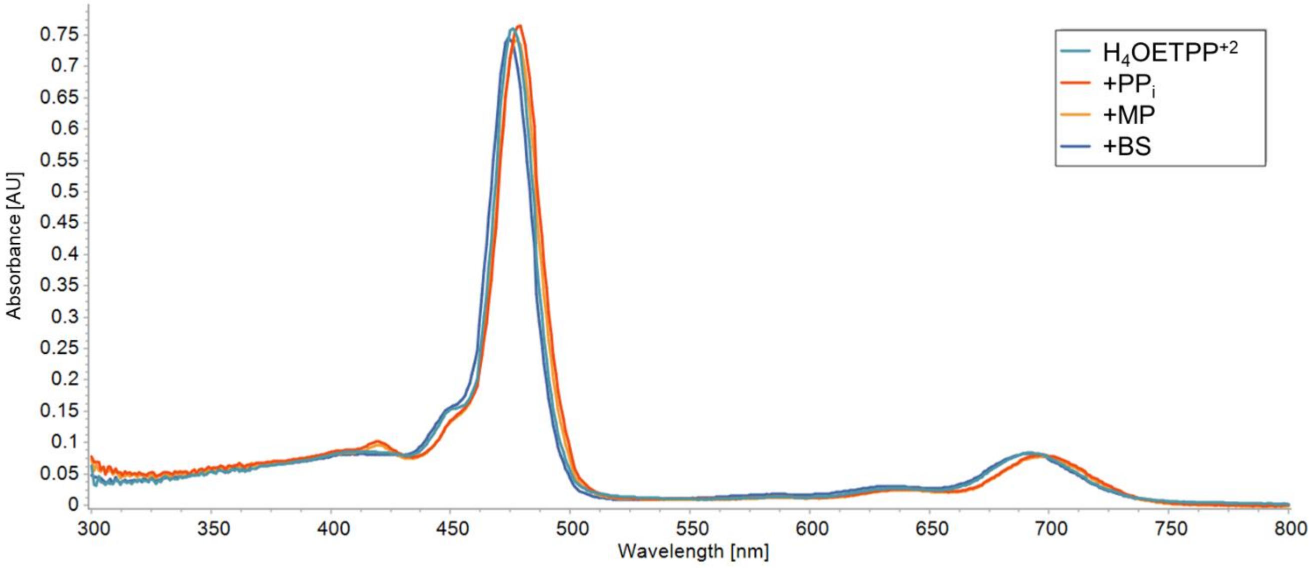


**Figure S47.** Overlay UV-vis spectra of $H_4OETPP^{+2}$ (3 × $10^{-6}$ M) and its interactions with 40 eq. of different analytes: pyrophosphate (**PP**$_i$), bisulphate (**BS**) and phosphate monobasic (**MP**) recorded in $CHCl_3$ in the presence of TFA (100 eq.).

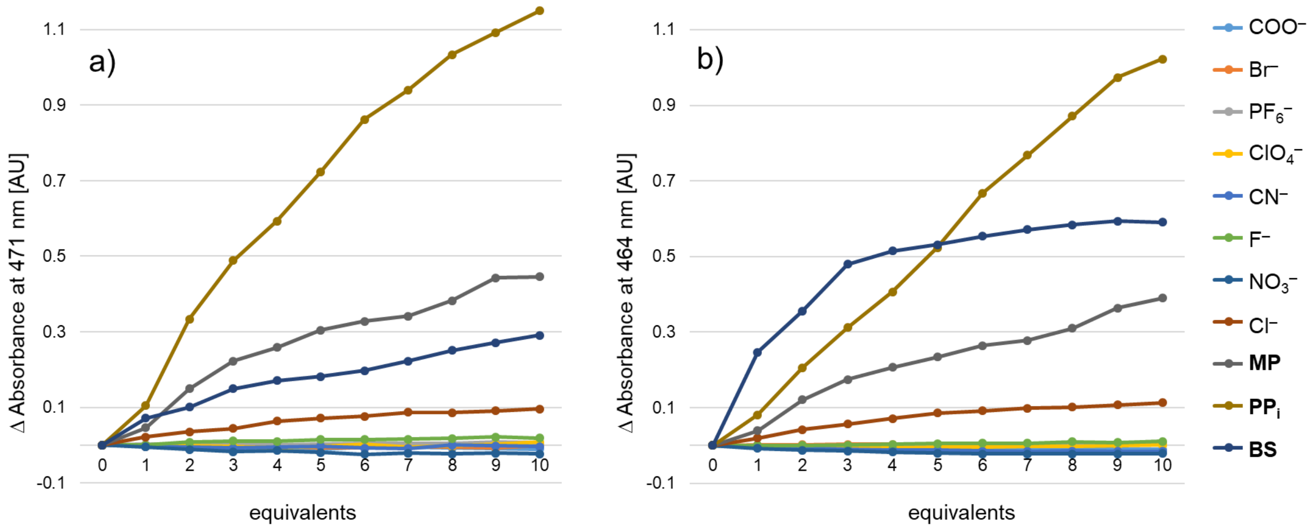


**Figure S48.** Concentration-dependant absorbance changes of *α4*-**1** (1.07 × $10^{-5}$ M) in the presence of TFA (100 eq.) following Soret bands at a) 471 nm [phosphonic compounds] and b) 464 nm [sulfonic compounds] with increasing concentration of various anions (up to 10 eq.): **MP**, **BS**, **PP**ᵢ, Br⁻, ClO4⁻, NO3⁻, PF6⁻, Cl⁻, CN⁻, COO⁻ recorded in $CHCl_3$.

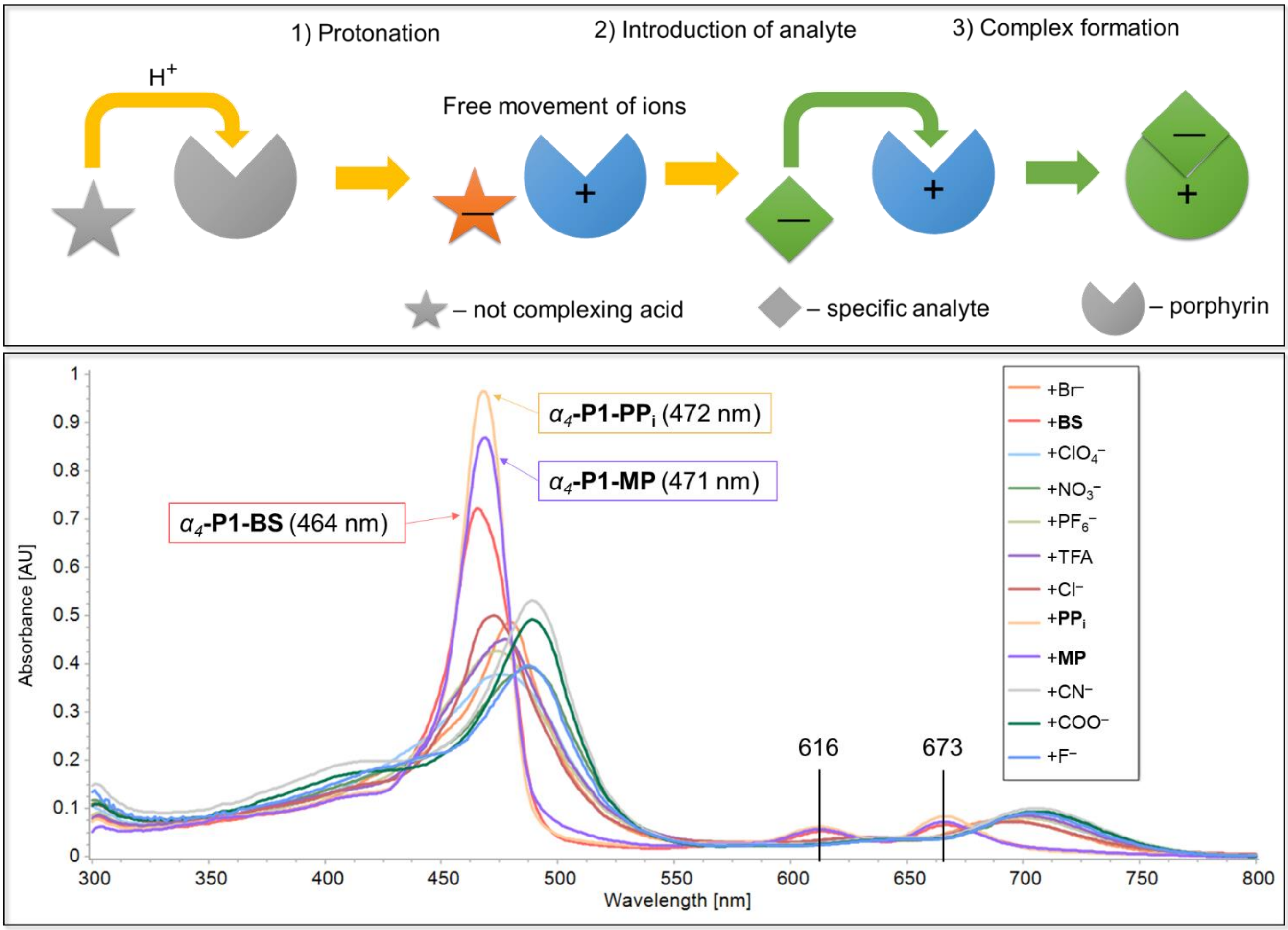


**Figure S49.** Top: schematic representation of complex formation with *α4*-**1**; bottom: overlay UV-vis spectra of *α4*-**1** (5.56 × $10^{-6}$ M) with TFA (500 eq.) and its interactions with strong excess (200 eq.) of different analytes: **MP**, **BS**, **PP**ᵢ, Br⁻, ClO4⁻, NO3⁻, PF6⁻, Cl⁻, CN⁻, COO⁻ recorded in $CHCl_3$.

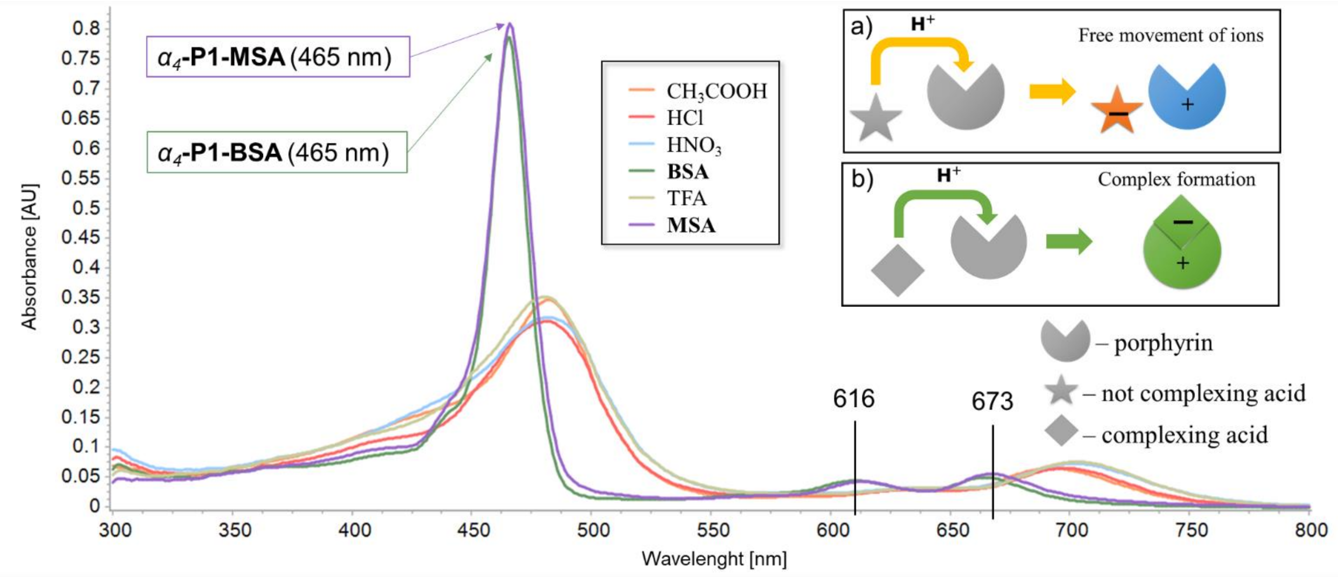


**Figure S50.** UV-vis studies of $\alpha_4$-**1** ($5.56 \times 10^{-6}$ M) protonation performed with different acids in $CHCl_3$ and graphical representation of protonation with: a) non-complexing acids; b) complexing acids.

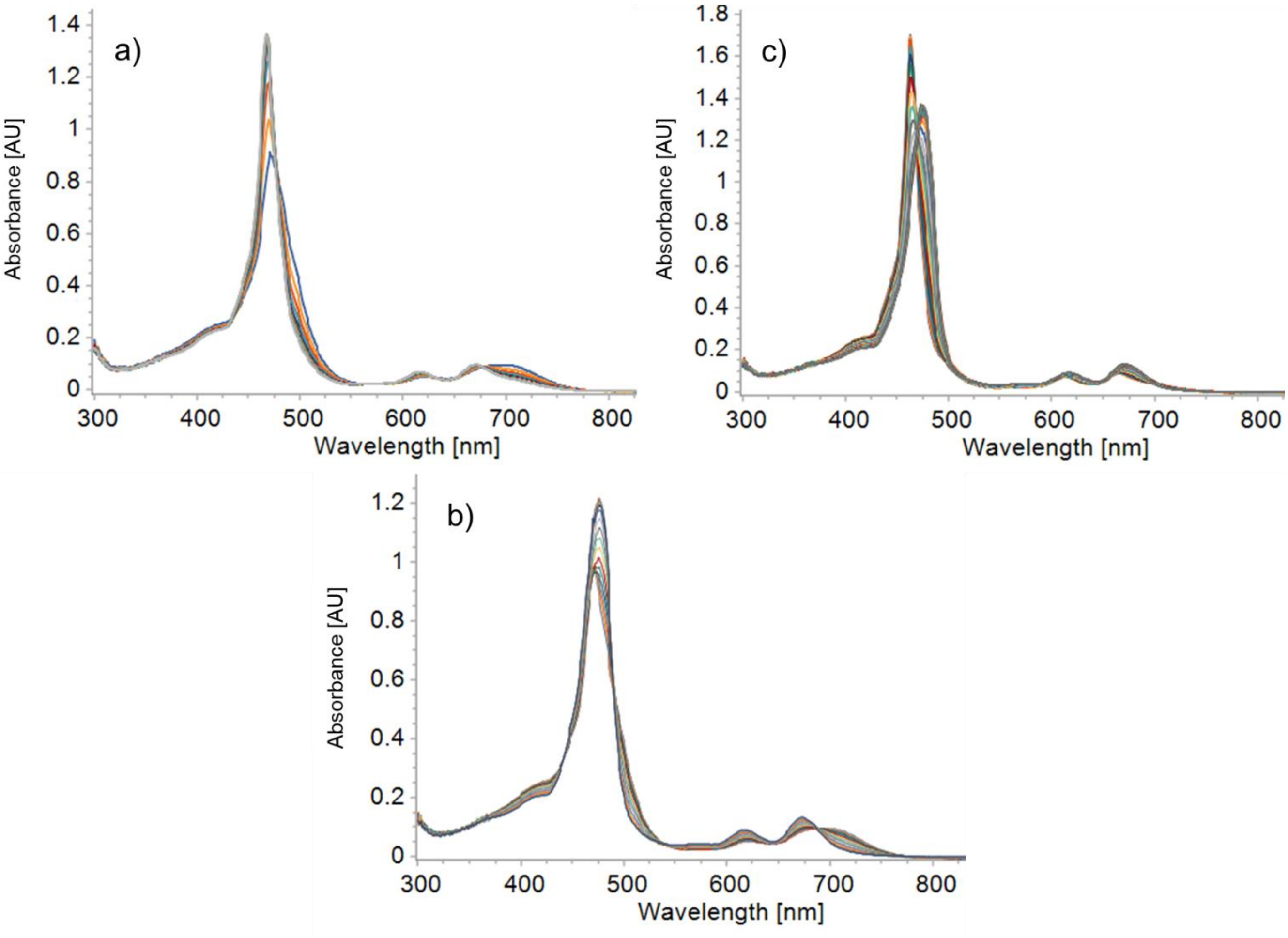


**Figure S51.** Displacement studies carried out with **BS**, **MP** and **PP$_i$** with $\alpha_4$-**1** ($1.07 \times 10^{-5}$ M) in the presence of TFA (100 eq.). UV-vis spectra is showing a) **BS** had displaced **MP** in the $\alpha_4$-**P1**-**MP** complex; b) **PP$_i$** displaced **MP** (in $\alpha_4$-**P1**-**MP**) and c) **PP$_i$** displaced **BS** (in $\alpha_4$-**P1**-**BS**). Note, **MP** had not displaced $\alpha_4$-**P1**-**BS** complex, moreover, neither **MP** nor **BS** displaced **PP$_i$** moiety in the $\alpha_4$-**P1**-**PP$_i$**.

**Binding and Competitive Studies**

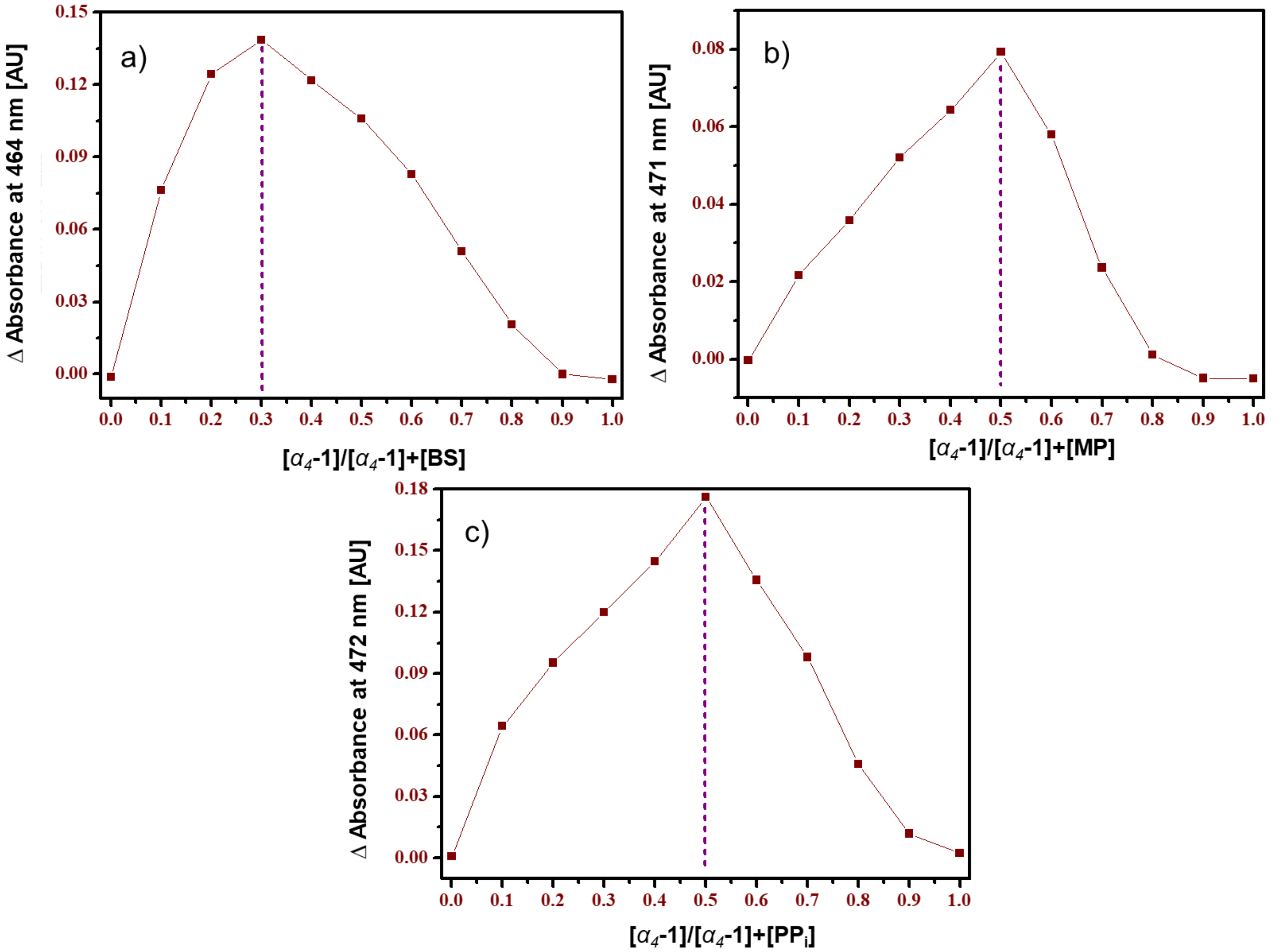


**Figure S52.** Job plots for the interaction between host $\alpha_4$-**1** and guests: a) **BS** (1:2 guest-to-host), b) **MP**(1:1 guest-to-host), c) **PP**$_i$, (1:1 guest-to-host) in $CHCl_3$ and 100 eq TFA with [host + guest] = 5.56 × $10^{-6}$ M.

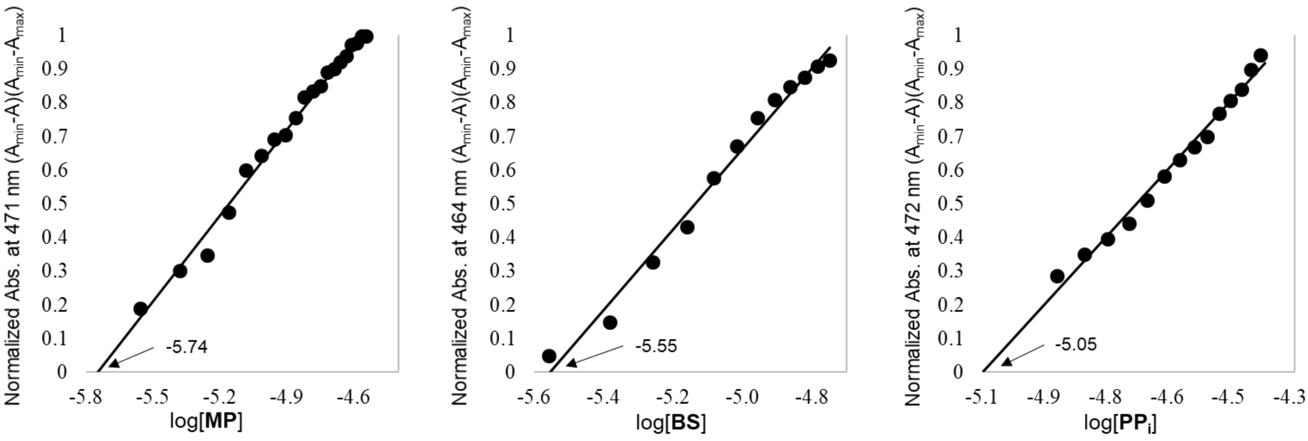


**Figure S53.** An analytical calibration curve using a simple liner curve fit to determine the limit of detection (LOD) for **MP**, **BS,** and **PP**$_i$. The porphyrin $\alpha_4$-**P1** is able to detect as low as ~8.84 µM for **PP**$_i$, ~2.83 µM for **BS**, and ~1.81 µM for **MP**.

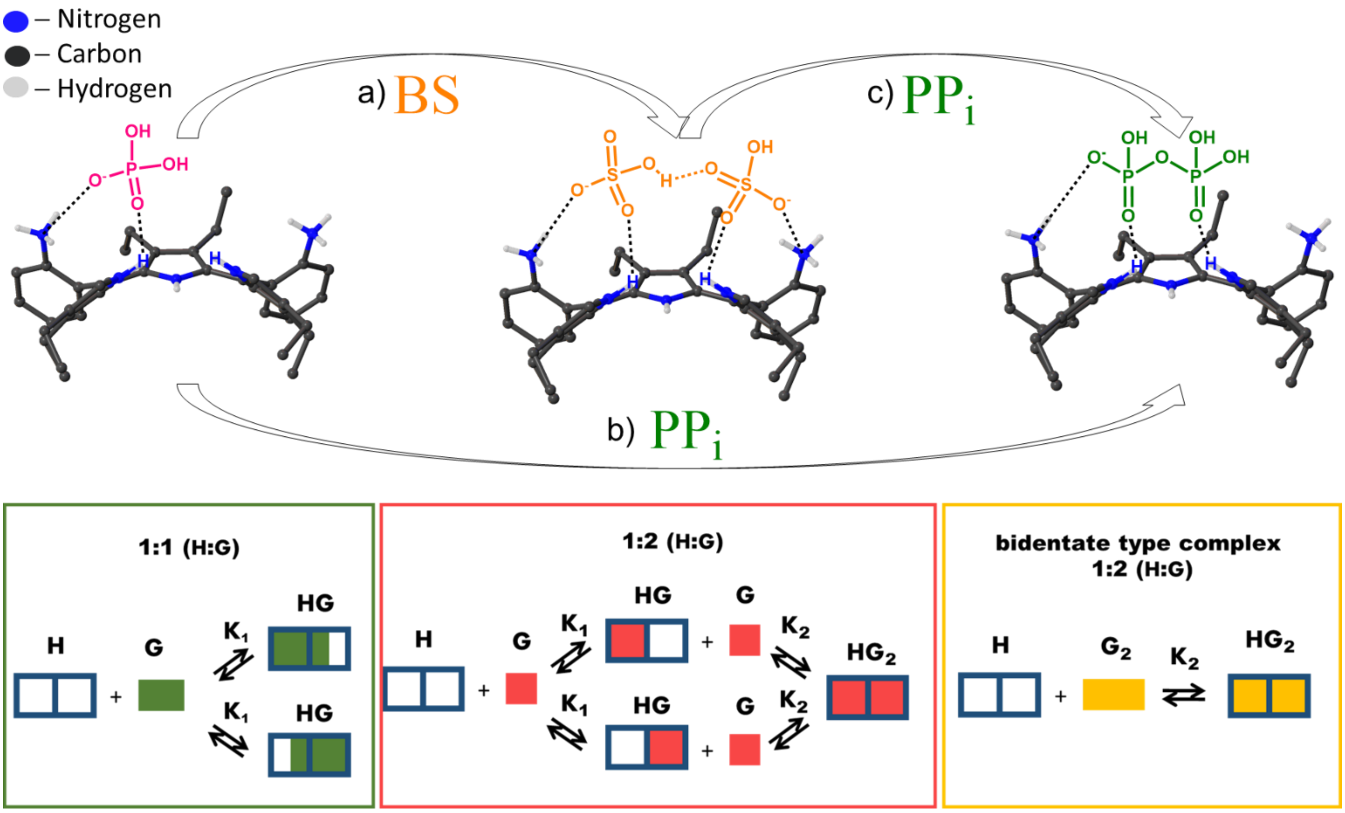


**Figure S54.** Graphical representation of the displacement studies (see also Figure S51).and likely binding motifs of analytes

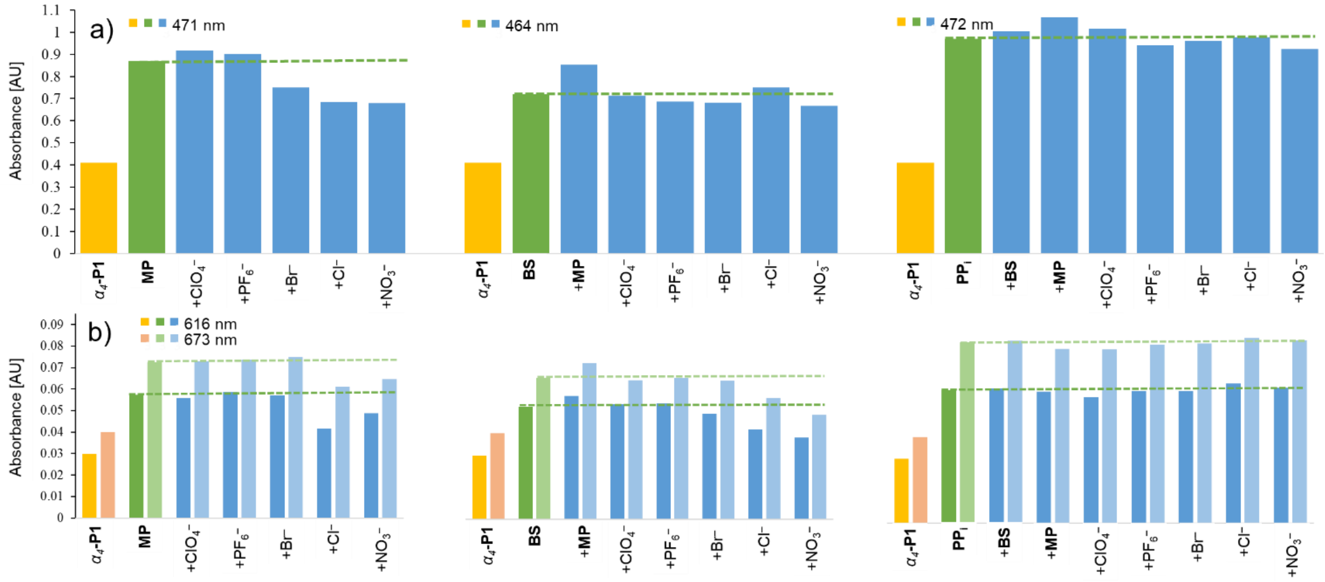


**Figure S55.** Ratiometric absorbance changes of *α4*-**1** (5.56 × $10^{-6}$ M) in $CHCl_3$, 500 eq. of TFA and 40 eq. of complexing substrates **MP**, **BS**, **PPi**, a) Soret bands: *α4*-**P1**-**MP** (471 nm), *α4*-**P1**-**BS** (464 nm), and *α4*-**P1**-**PPi** (472 nm), b) Q-bands (616 and 673 nm) in the presence of 200 eq. of other anions. It is worth mention, commonly used interfering anions: fluoride, cyanide, and acetate were not used in the competition experiments due to high basicity, thus, leading to the turn off sensor system.

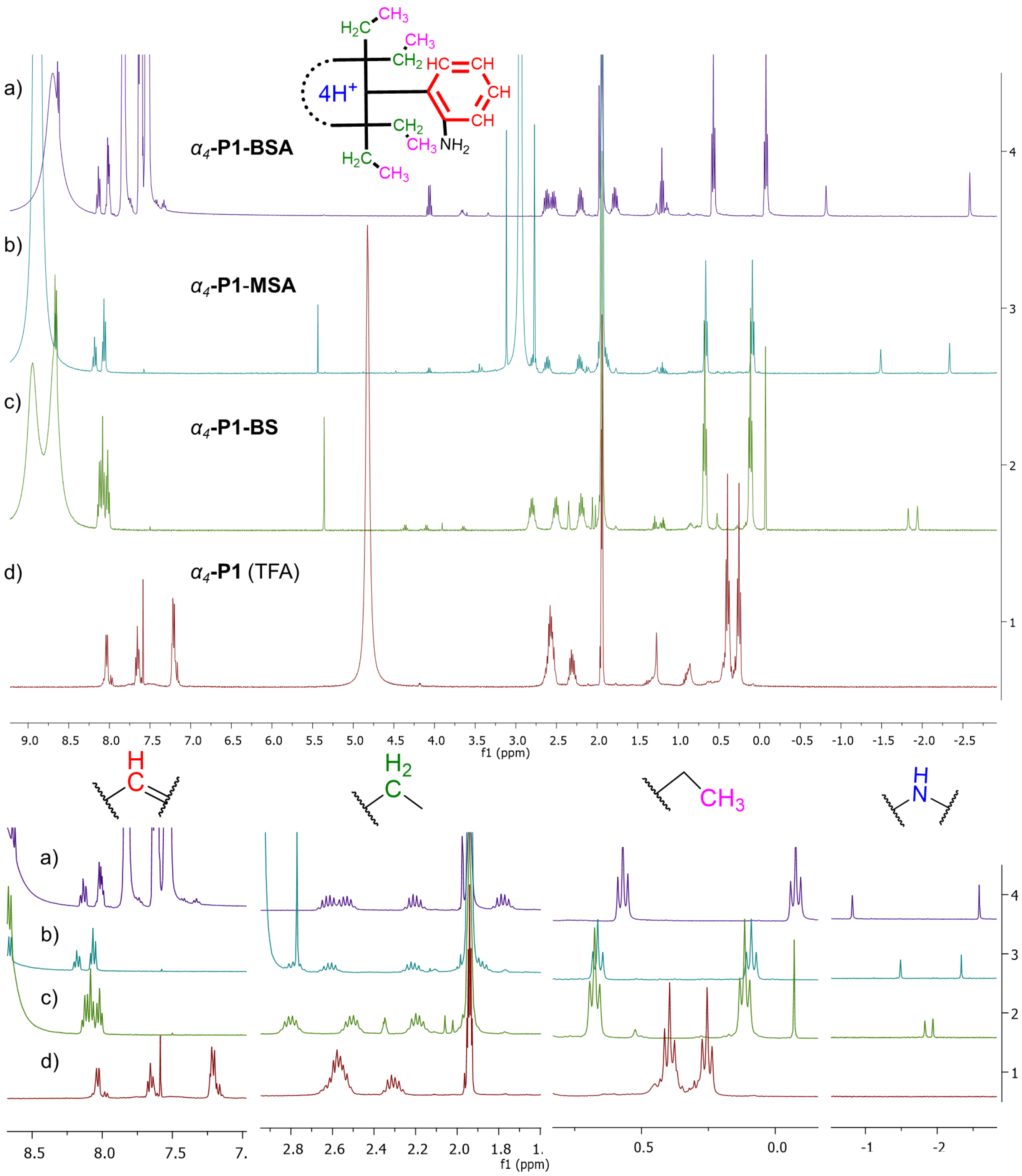


**Figure S56.** Top: $^1$H NMR spectra (400 MHz, *d*-acetonitrile, 25 °C) of d) $\alpha_4$-**P1** and different complexes: a) $\alpha_4$-**P1-BSA**, b) $\alpha_4$-**P1-MSA**, c) $\alpha_4$-**P1- BSA**. Bottom: expansion of $^1$H NMR areas of interest.

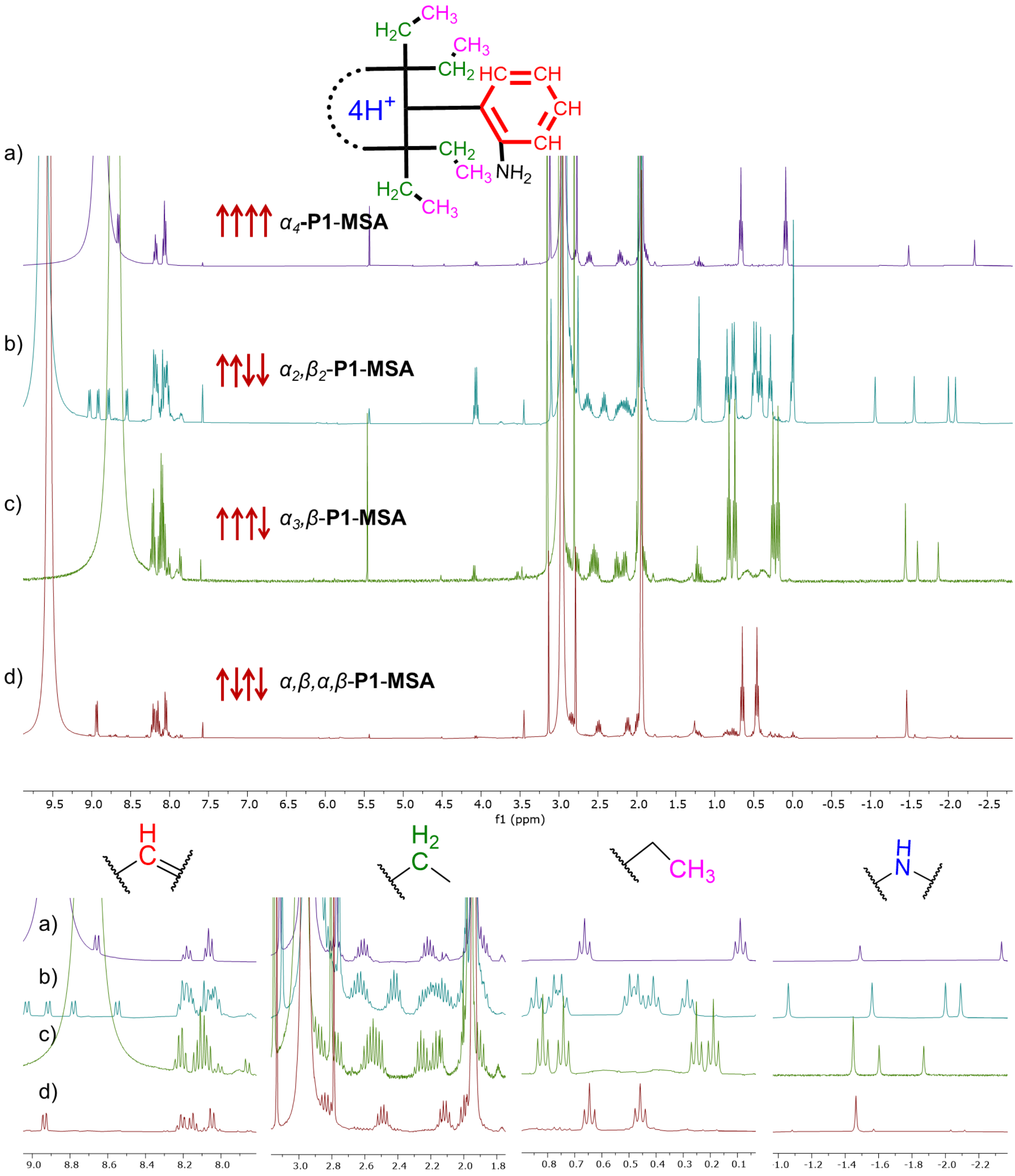


**Figure S57.** Top: $^{1}H$ NMR spectra (400 MHz, *d*-acetonitrile, 25 °C) of individual atropisomer **1** complexes with **MSA**: a) *α₄*-**P1**-**MSA**, b) *α₂,β₂*- **P1**-**MSA**, c) *α₃,β*-**P1**-**MSA,** and d) *α,β,α,β*-**P1**-**MSA**. Bottom: expansion of $^{1}H$ NMR areas of interest.

## References

[1] APEX3, Version 2016.9-0, Bruker AXS, Inc., Madison, WI, **2016**.
[2] SADABS, Version 2016/2, Bruker AXS, Inc., Madison, WI, **2014**.
[3] W. Jentzen, X.-Z. Song, J. A. Shelnutt, *J. Phys. Chem. B* **1997**, *101*, 1684–1699.
[4] L. Sun and J. A. Shelnutt, NSDGUI (Version 1.3 Alpha version), Sandia National Laboratory, Albuquerque, USA., **2000–2001**.
[5] B. Zimmer, V. Bulach, C. Drexler, S. Erhardt, M. W. Hosseini, A. De Cian, *New J. Chem.* **2002**, *26*, 43–57.
[6] C. R. Groom, I. J. Bruno, M. P. Lightfoot, S. C. Ward, *Acta Crystallogr.* **2016**, *B72*, 171–179.
[7] M. P. Byrn, C. J. Curtis, I. Goldberg, Y. Hsiou, S. I. Khan, P. A. Sawin, S. K. Tendick, C. E. Strouse, *J. Am. Chem. Soc.* **1991**, *113*, 6549–6557.
[8] H. Hope, *Prog. Inorg. Chem.* **2007**, *41*, 1–19.
[9] a) O. V. Dolomanov, L. J. Bourhis, R. J. Gildea, J. A. K. Howard, H. Puschmann, *J. Appl. Cryst.* **2009**, *42*, 339–341; b) G. Sheldrick, *Acta Crystallogr.* **2015**, *A71*, 3–8.
